# Selection of optimised ligands by fluorescence-activated bead sorting†

**DOI:** 10.1039/d3sc03581f

**Published:** 2023-08-11

**Authors:** Alexandra R. Paul, Mario Falsaperna, Helen Lavender, Michelle D. Garrett, Christopher J. Serpell

**Affiliations:** a School of Chemistry and Forensic Sciences, Division of Natural Sciences, University of Kent Canterbury CT2 7NH UK; b Avvinity Therapeutics 66 Prescot Street London E1 8NN UK; c School of Biosciences, Division of Natural Sciences, University of Kent Canterbury CT2 7NJ UK m.d.garrett@kent.ac.uk; d School of Pharmacy, University College London London WC1N 1AX UK chris.serpell@ucl.ac.uk

## Abstract

The chemistry of aptamers is largely limited to natural nucleotides, and although modifications of nucleic acids can enhance target aptamer affinity, there has not yet been a technology for selecting the right modifications in the right locations out of the vast number of possibilities, because enzymatic amplification does not transmit sequence-specific modification information. Here we show the first method for the selection of specific nucleoside modifications that increase aptamer binding efficacy, using the oncoprotein EGFR as a model target. Using fluorescence-activated bead sorting (FABS), we have successfully selected optimized aptamers from a library of >65 000 variations. Hits were identified by tandem mass spectrometry and validated by using an EGFR binding assay and computational docking studies. Our results provide proof of concept for this novel strategy for the selection of chemically optimised aptamers and offer a new method for rapidly synthesising and screening large aptamer libraries to accelerate diagnostic and drug discovery.

## Abstract 2

Chemical modifications improve aptamer properties, but it has been challenging to work out the best modifications out of all the possibilities. We present an automated bead-based system for selecting the best locations and types of chemical modifications to improve the affinity of aptamers.

## Graphical abstract

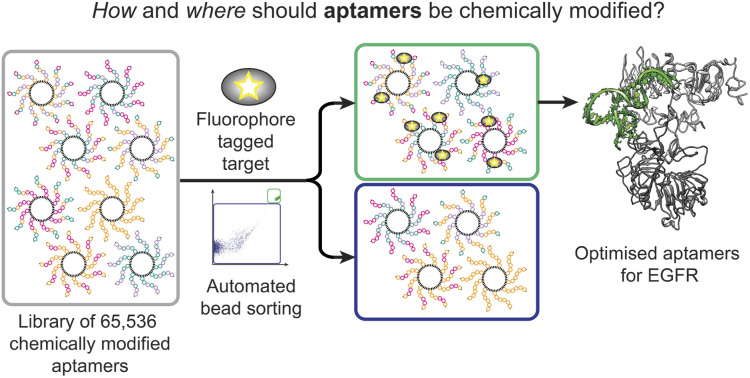

## Introduction

Aptamers are single-stranded oligonucleotides that mimic antibodies by folding into complex 3D shapes that non-covalently bind with high affinity and specificity to diverse targets.^1^ Aptamers have been selected against viruses, proteins,^2^ polysaccharides,^3^ bacteria,^4^ toxins,^5^ peptides,^6^ small molecules,^7^ amino acids^8^ and whole cells.^9^ Like antibodies, the recognition capacity of aptamers can be harnessed for the development of diagnostics and therapeutic agents. Aptamers have advantages over antibodies in that they are selected in a chemically controlled environment, which does not rely on eliciting an immune response, their lower molecular weight improves pharmacokinetics, their precise synthesis^10–12^ is low cost and provides possibility for comprehensive chemical modification,^1^ and they have a longer shelf life.^13^ Theoretically, aptamers can be used as therapeutics in any disease, particularly those in which extracellular blockade of proteins is needed.^14^ The global aptamer market size is estimated to value USD242 million in 2022 and is expected to grow to USD524 million by 2027.^15–17^

Systematic Evolution of Ligands by EXponential Enrichment (SELEX)^18,19^ is the standard procedure used for selecting aptamers for a particular target. SELEX starts with a large library of oligonucleotides (up to 10^14^ sequences),^20^ followed by rounds of selection consisting on separating binders from non-binders, followed by PCR-based amplification,^1^ and ending with sequencing.^21^ Although thousands of aptamers targeting a broad range of targets have been generated by SELEX, outstanding aptamers that can be reliably used in biomedical and analytical applications are still limited because there are gaps in their performance including their specificity and affinity.^22^ To overcome these hurdles, researchers have been investigating methods to create aptamers using larger library sizes and with more complex structures.^23^ Non-natural modifications of nucleic acids are known to enhance aptamer affinity,^24^ and can be discovered by using enzymes which will accept modified nucleotide triphosphates (N*TPs), however this results in uniform modification – for example, with all uridines displaying a cubane appendage^25^ – since information on variations of modification at the same type of base would be lost in PCR amplification. Alternatively, a codon system has been introduced,^26^ but again this does not allow the modifications to evolve independently of the base sequence. Systematic manual alterations of pre-selected aptamers has been reported, but is laborious.^27^ Selecting both the correct type and sequence of modifications amongst masses of possibilities, independent of the base sequence has not previously been achieved. We herein report a method for identification of the location and type of chemical modifications to an existing aptamer to improve its affinity – selection of optimised ligands by fluorescence-activated bead sorting (SOLFABS).

The method achieves this by combining high-precision automated flow cytometry-based selection with enzyme-free sequence readout, retaining all the information concerning modification locations. This will further boost the future success of aptamers as therapeutics, targeting agents, and in diagnostics.

## Results

**Fig. 1:**
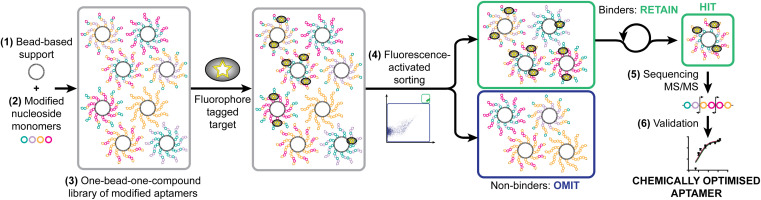
Strategy for the synthetic selection of enhanced therapeutic aptamers illustrating necessary steps: identification of (1) appropriate solid support; (2) modified nucleoside monomers; (3) synthesis of one-bead-one-compound library; (4) fluorescence-activated sorting to achieve selection; (5) identification of hit sequences by MS/MS; and (6) validation through EGFR binding assays and modelling.

As a model system, we have taken a therapeutically relevant aptamer (MinE07)^28,29^ which binds to the epidermal growth factor receptor (EGFR), a transmembrane receptor tyrosine kinase which is a validated extracellular target in the treatment of cancer.^30^ Our approach was to create a one-bead-one-compound library of modified aptamers which could then be sorted using a flow cytometer which would provide accurate control of selection gating and in-line analytical data, followed by sequencing by mass spectrometry, and validation. We identified six key steps necessary for the SOLFABS process: (1) choice of a solid support that is homogeneous in size, suitable for nucleic acid synthesis, and compatible with the flow cytometer; (2) design, synthesis and purification of a range of modified nucleosides; (3) synthesis of the one-bead-one-compound library; (4) development of FABS methods for identification and isolation of high affinity modified aptamers; (5) tandem mass spectrometry to identify the position of type of the selected modifications; and (6) an EGFR binding assay (producing a *K*_d_) to validate the affinity of the nucleoside modified aptamers for EGFR *versus*MinE07 (Fig. 1).

### Identification of solid support

A key requirement for FABS is use of uniform beads that can pass through the cytometer tubing and in front of the lasers as single beads. TentaGel® M NH_2_ Monosized Amino TentaGel Microspheres (TG-beads) have a polystyrene backbone with a PEG spacer attached *via* alkyl linkage,^31^ which is resistant to acids and bases and so suitable for phosphoramidite chemistry.

**Fig. 2:**
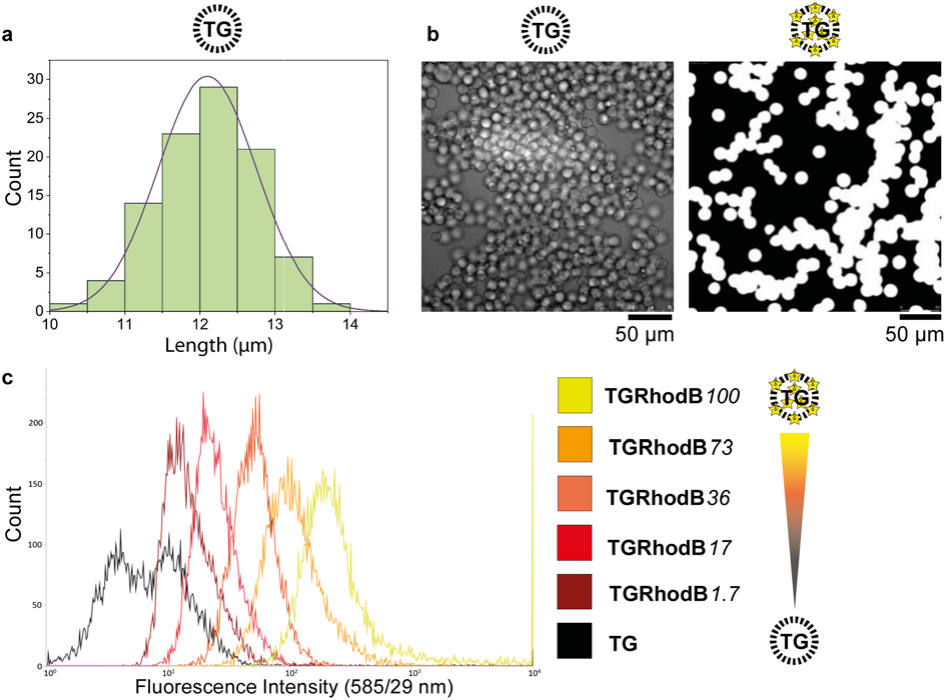
(a) A Gaussian distribution curve demonstrating the range of TG diameters as measured by SEM. The sample gave a mean of 12.1 μm and a standard deviation of 0.7 μm. (b) Unlabelled (left) and fluorophore-labelled TG beads imaged by SEM and fluorescence microscopy respectively. Intensity scales have been normalised. (c) Detection of differing levels of fluorophore labelling by flow cytometry. The rhodamine B tagged microspheres were detected by flow cytometry using the laser with excitation 561 nm and emission 585/29 nm.

To be certain of microsphere size and uniformity, the diameters of a sample of TG-beads were measured using scanning electron microscopy (SEM), giving a mean bead diameter of 12.1 ± 0.7 μm (Fig. 2a). This was suitable for flow cytometry, as the tubing allows beads of up to 20 μm diameter to pass. Light and fluorescence microscopy also confirmed bead uniformity (Fig. 2b). The linker 10-hydroxydecanoic acid was then attached to the amines of the TG-beads to provide an alcohol terminus for coupling of phosphoramidites. The unmodified MinE07 aptamer was synthesised on the beads to check the linker attachment and optimise the synthesis method. To ensure the compatibility of the TG-beads with the flow cytometer and to analyse the sensitivity of its fluorescent detector, a range of fluorescently labelled TG-beads were prepared. TG-beads were labelled with rhodamine B at 100% (TGRhodB100), 73% (TGRhodB73), 36% (TGRhodB36), 17% (TGRhodB17) and 1.7% (TGRhodB1.7) loading. The eight fluorescently labelled TG-bead samples were passed through the flow cytometer to detect the level of fluorescence compared with the unlabelled TG-beads. The histograms show that the flow cytometer can distinguish between different intensities of fluorescence, that the TG-beads could be detected and counted by the flow cytometer as beads, and that in this setup bead autofluorescence is negligible compared with genuine dye emission (Fig. 2c). In summary, we have shown that TG-beads that are suitable for aptamer synthesis are also compatible for fluorescent-based TG-bead sorting by flow cytometry.

### Synthesis of modified monomers

We chose to modify the uridine (U) monomers at the C5 position since this is synthetically accessible,^32^ and gives modifications of medical relevance^33–35^ which would sufficiently validate our method. The modifications were designed to cover aryl (–Ph) and aliphatic hydrophobic (–Vi), halogenated (–I), and natural uridine (*i.e.* H) substituents, potentially enhancing or disrupting interactions through steric hindrance, hydrophobic effects, π-stacking, or halogen bonding.^36,37^MinE07 uses nucleosides in which the 2′-hydroxyl has been replaced by fluorine on the pyrimidine nucleotides (fU and fC); this modification was retained in our monomers. All modified monomers (fU-Ph, fU-Vi, and fU-I) were synthesised through iodination and carbon–carbon cross-coupling reactions as needed, with appropriate protecting groups to give dimethoxytrityl-protected activated phosphoramidites suitable for automated oligonucleotide synthesis (S3, Fig. 1–45†). Combined with fU from the original aptamer, this gave four variations on U, and taking into account the eight U positions on MinE07, it gives 4^8^ = 65 536 unique sequences of modifications.

### Aptamer library synthesis

**Fig. 3:**
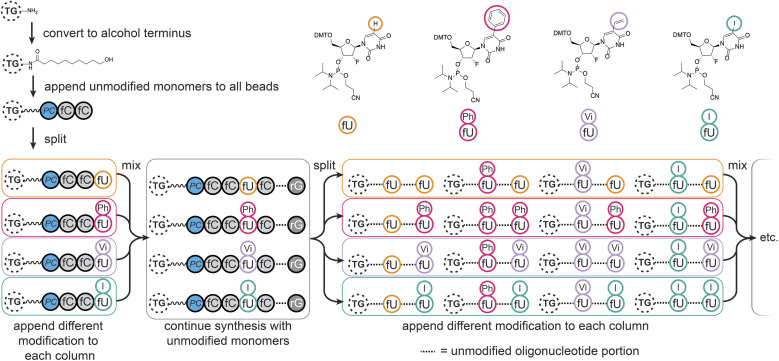
Synthesis of the MinE07Library on TentaGel (TG) beads, starting with conversion of the amine to alcohol functionality, appendage of the photocleavable linker (PC), followed by oligonucleotide synthesis with split-and-mix steps at each uridine using the fU, fU-Ph, fU-Vi, and fU-I phosphoramidite monomers as shown.

Automated synthesis of MinE07Library was conducted by combining our monomers (fU-Ph, fU-Vi, and fU-I) with commercially made phosphoramidites (rA, rG, fC, fU) and using the hydroxy-modified TG-beads (Fig. 3). After swelling in dichloromethane, a photocleavable linker^38^ was added to all beads so that after selection the aptamers can be liberated for analysis using UV light. Following this step, cycles of automated phosphoramidite synthesis were performed, with split-and-mix procedures at each instance of U. The final library contained on average 67 beads of every possible set of modifications, each displaying 1.17 × 10^11^ copies of the strands. The trityl monitor was used as a semiquantitive check on synthetic efficiency (S4, Table 1†).

### Fluorescence-activated bead sorting

**Fig. 4:**
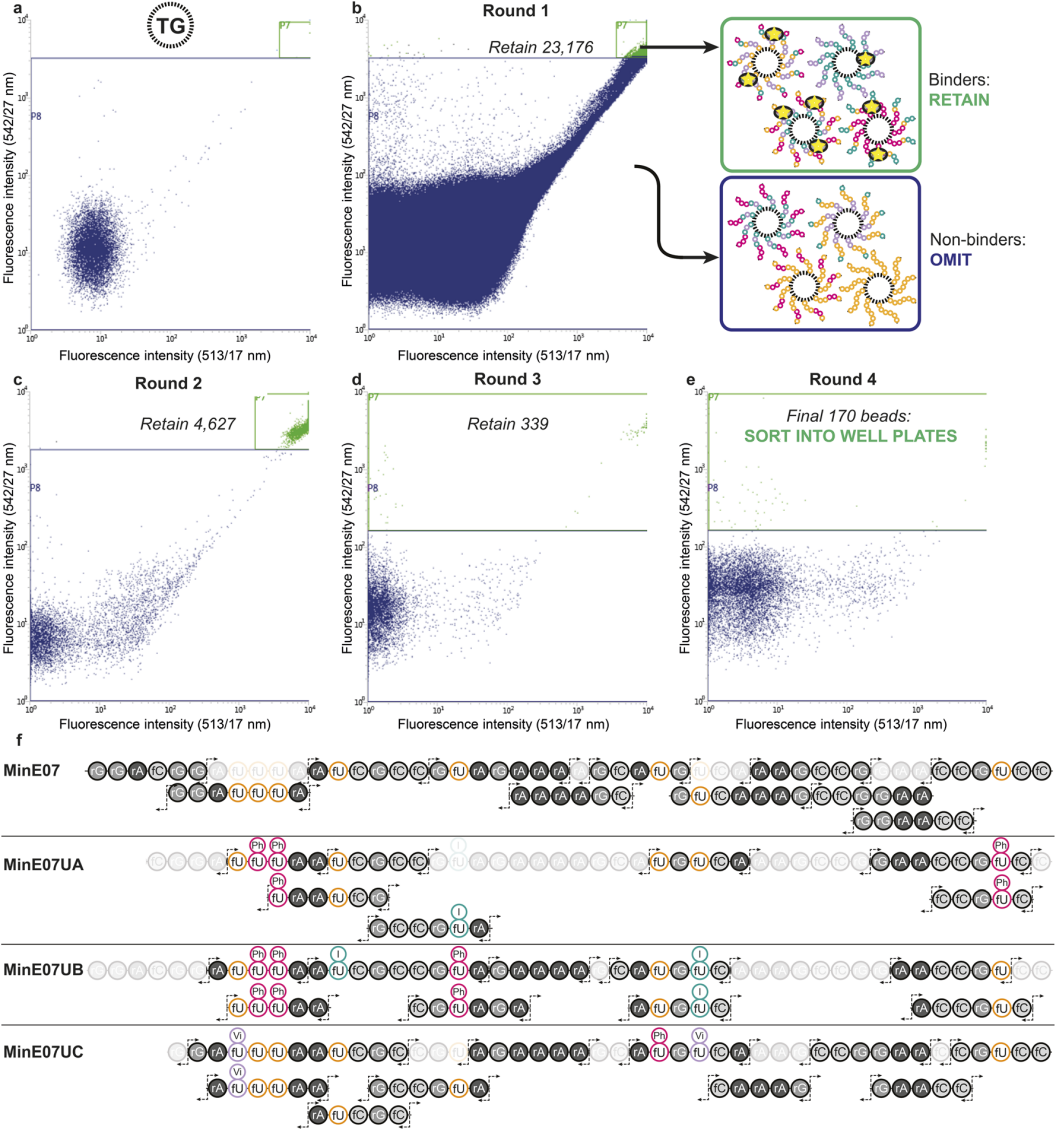
(a) Flow cytometry data of the non-labelled TG-beads to select gating for sorting. (b) Flow cytometry data from round 1 of fluorescence activated bead sorting (FABS) of the 5 648 248 MinE07Lib beads incubated with EGFR-Fc and protein A-FITC. Two-way sort into binders and non-binders of EGFR under a competitive regime. (c) and (d) Flow cytometry data for rounds 2 and 3 of FABS (e) flow cytometry data for round 4 of FABS in which final hits were dispensed into individual wells. Note that large number of low-intensity events are water background, *i.e.* drops with no bead. (f) RNA oligomer fragmentation products produced by collision induced dissociation for the parent MinE07 aptamer and the three hits, MinE07UA, MinE07UB, and MinE07UC. The most abundant fragment ions (c- and y-fragments as well as w- and a–b-ions) are indicated by the fragmentation arrows. Fragments not found, or unable to assign with certainty due to short length (<5 bases), are shaded out. Their presence in the product is confirmed by the primary ion mass spectrum.

The flow cytometer is able to measure fluorescence on a particle-by-particle basis. This provides an analytical readout which can be used to refine appropriate gates to determine at what level of signal the particle will be sorted into a specific container – this is known as fluorescence-activated bead sorting (FABS). FABS has previously been used in combinatorial chemistry,^39,40^ including for aptamer discovery,^41–44^ but despite offering automation, real-time analytical output, and controllable multichannel gating, its adoption has lagged behind other methods.^45^ We adapted FABS for selection of aptamer modifications by incubating MinE07Library with recombinant EGFR protein (extracellular domain only, amino acids 1–620) displaying an Fc-tag (the Fc-tag does not affect the interaction between MinE07 and EGFR, as proven by binding studies, *vide infra*), and equimolar protein A conjugated to a FITC fluorophore. Protein A-FITC binds to the Fc-tag to provide fluorescence to any bead-based aptamer to which EGFR binds. Beads with the highest affinity for EGFR would therefore be the most fluorescent. The non-labelled TG beads were first run through the FACS machine to optimise the conditions and gating (Fig. 4a). As there are no cell populations to gate for, it is best practice to gate on both 513/17 nm and 542/27 nm emissions (two closest wavelengths for FITC) for the bead sorting to reduce false positives. The aptamer library was then incubated with enough EGFR-Fc and protein A-FITC to completely cover just 96 beads – *i.e.*, a high stringency regime in which only the beads with the highest affinity aptamers for EGFR would acquire fluorescence. The mixture was then subjected to the first round of FABS, which selected 23 176 beads out of a total of 5 648 248 (Fig. 4b). Subsequent rounds of selection increased stringency, meaning that there was less protein to ‘go around’, and therefore some beads which may have been strong binders in prior rounds could no longer compete for the protein, and no longer displayed fluorescence. The second round of FABS resulted in 4627 sorted beads, which then underwent a third round, to give 339 beads. In a final round, the top 170 beads were then sorted individually in well plates containing pure water (Fig. 4c–e). The aptamers were then photocleaved from the TG-beads in their wells.

### Sequencing by mass spectrometry

**Fig. 5:**
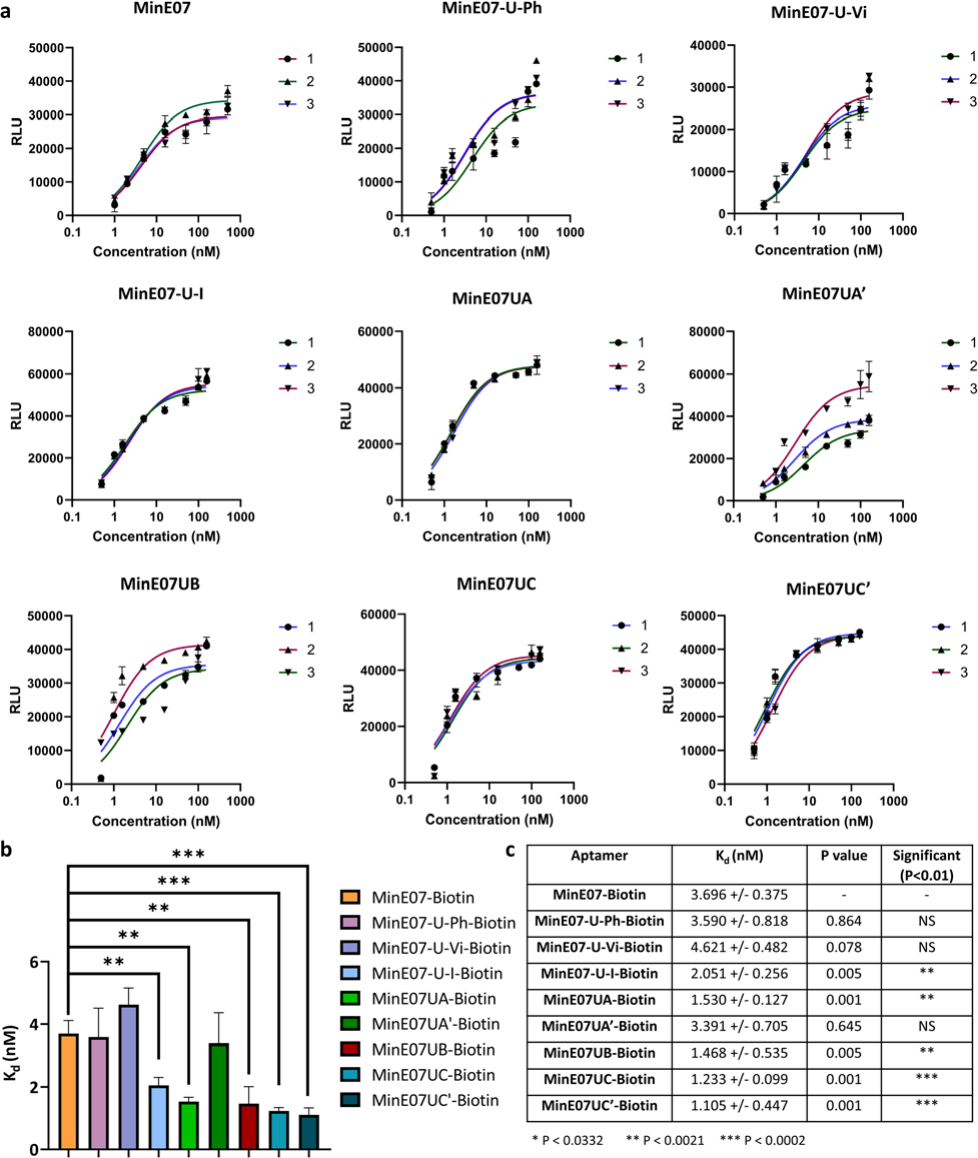
(a) Graphs of EGFR affinity assay results for MinE07–Biotin, MinE07-U-Ph–Biotin, MinE07-U-Vi–Biotin, MinE07-U-I–Biotin, MinE07UA–Biotin, MinE07UA′–Biotin, MinE07UB–Biotin, MinE07UC–Biotin, and MinE07UC′–Biotin. Numbers 1–3 indicate the 3 assay repeats for each aptamer (*n* = 3) (b) graph of all modified aptamers experimental *K*_d_ values (*n* = 3) (error bars: standard deviation). (c) Table of all modified aptamers experimental *K*_d_ values (*n* = 3, ±SD) and level of significance measured by *t*-test analysis. Confidence intervals given in S8 Table 2.†

Identification of sequences, including location and type of modification, was achieved by reverse-phase liquid chromatography coupled with secondary ion mass spectrometry (MS/MS, Fig. 4f). In MS/MS, fragments are produced by breaks in the oligonucleotide chain caused by the collision-induced dissociation.^46^ In the 5′ direction the c-notation is the nucleotide molecular weight and the ab(*n*) notation is c(*n* − 1) + (B(*n*) − base MW). In the 3′ direction y notation is the nucleotide molecular weight and w(*n*) = y(*n*) + PO_3_H molecular weight (80 Da).^47^ The most abundant ions observed were c- and y- and w- fragments, with ab- also observed.^48^ Fifteen sorted MinE07Library aptamers were chosen at random from the 170 sorted aptamers, of which three gave high quality MS/MS data and were named MinE07UA, MinE07UB and MinE07UC (the 12 remaining beads gave data below the limit of detection for our MS/MS instrument). The mass spectrum of MinE07UA showed a mass of 15 136.2 Da which indicates that we had isolated a bead on which the synthesis was incomplete since the MinE07Library mass range is 15 677–16 693.2 Da (S6, Fig. 70†). It is inevitable that even in the best optimised synthesis, some instances of incomplete strands will occur, and should they prove to be improved binders, they will end up enriched in the selected sequences; this ‘bug’ can be viewed as a ‘feature’ since it has the benefit of further increasing library diversity. Beads displaying a uniform truncation of their strands could occur due to imperfect distributions in the flow of reagents over the immobile beads during synthesis. Upon analysis of the MS/MS fragment data using RoboOligo,^49^ it was found that the aptamer had been capped at base 45 (fC) meaning 5′-rGrGrA was missing. Uridines 2, 3, and 8 (U2, U3, U8) were fU-Ph and U5 was fU-I (S6, Fig. 71–84†). The mass spectrum of MinE07UB showed a mass of 16 150.9 Da (S6, Fig. 85†), a full-length aptamer, with U2, U3, and U5 being fU-Ph and U4 being fU-I (S6, Fig. 86–103†). MinE07UC displayed a mass of 14 536.0 Da, which again indicates a truncation; in this case the 5′-rGrGrAfC sequencing was missing (S6, Fig. 104†). The fragment data shows that U1 and U7 were fU-Vi and U6 was fU-Ph (S6, Fig. 105–126†). The sequences MinE07UA and MinE07UB are similar in that they both have three fU-Ph modified uridines, two of which are in the same location, and both have at least one fU-I modification (Fig. 5). Potentially the two same fU-Ph modified uridines locations are important in increasing affinity. MinE07UC also has a fU-Ph modified uridine, however not in the same locations as the others. These results confirm that aptamer modifications can be selected for their targets using FABS and then identified using LC-MS/MS.

### Validation of affinity optimisation

The original MinE07 aptamer was synthesised, along with the three hit modified aptamers, each with an additional 5′-biotin, at the experimentally identified truncated length (MinE07UA–Biotin, MinE07UB–Biotin and MinE07UC–Biotin), and also the expected full-length versions of the truncated hits (MinE07UA′–Biotin, MinE07UC′–Biotin) for validation (S7, Fig. 127–141†). Control aptamers MinE07-U-Ph–Biotin, MinE07-U-Vi–Biotin and MinE07-U-I–Biotin, which have uniform modification of all their uridines (comparable with the state of the art for modified aptamers)^50,51^ were also synthesised (S7, Fig. 142–148†). The strands were purified by polyacrylamide gel electrophoresis, and confirmation of the full-length product was obtained by performing a 4′-hydroxyazobenzene-2-carboxylic acid (HABA) assay (S7, Fig. 149†). This reports the presence of biotin, which can only occur in full length strands as it is the final (5′) monomer, and the capping step in the synthesis would prevent its addition to truncated strands. HABA can be displaced from the binding site of streptavidin by biotin.^52^ Protein A plates (96-well) were incubated with EGFR-Fc, after which aptamers were added followed by streptavidin–alkaline phosphatase (AP) and an AP chemiluminescent substrate. In the absence of biotin, HABA quenches chemiluminescence *via* FRET.^53^ All the newly synthesised aptamers gave a positive signal and were carried forward for *K*_d_ determination in an ELISA-based EGFR binding assay. The parent aptamer, MinE07–Biotin, produced a *K*_d_ of 3.70 ± 0.38 nM consistent with the literature (Fig. 5 and S8 Fig. 150†).^28^MinE07-U-Ph–Biotin, MinE07-U-Vi–Biotin, and MinE07-U-I–Biotin gave *K*_d_ values of 3.59 ± 0.82 nM, 4.62 ± 0.48 nM, and 2.05 ± 0.26 nM respectively (Fig. 5 and S8 Fig. 151–153†), showing that modifying all uridines with a phenyl has little overall change on affinity, while uniform modification with a vinyl group weakens the binding, and iodo modification on all uridines gives a modest increase in affinity. This shows that uniform modification cannot reliably increase aptamer affinity of a previously selected nucleobase sequence. Looking at the selected sequences, MinE07UA–Biotin gave a *K*_d_ of 1.53 ± 0.13 nM, while the un-truncated version resulted in a *K*_d_ of 3.39 ± 0.71 nM (Fig. 5, S8 Fig. 154 and 155†). The selected series of modifications, including truncation, is a significant improvement, whereas attempting to repair the truncation actually impaired affinity – this is an unexpected but useful result which has arisen from the imperfections in synthesis being highlighted by the precision of the selection methodology – although the synthesis “failed”, the selection was still successful. MinE07UB–Biotin displayed a *K*_d_ of 1.47 ± 0.54 nM (Fig. 5 and S8 Fig. 156†), again, a significantly higher affinity for EGFR compared to MinE07. MinE07UC–Biotin gave a *K*_d_ of 1.23 ± 0.10 nM, again illustrating that selection consistently identified improved sequences of modifications. Here, however, the full-length version MinE07UC′–Biotin resulted in a *K*_d_ of 1.11 ± 0.45 nM (Fig. 5 and S8 Fig. 157–159). Overall, we can see that FABS was essential to the identification of aptamers with a higher affinity for EGFR than MinE07; the exact number, type, and position of modifications was critical in creating a more successful aptamer, with the most promising aptamers (MinE07UA and MinE07UC) having binding affinities up to 3.3-fold stronger than that of the parent aptamer.

### Computational docking studies of aptamers with EGFR

**Fig. 6:**
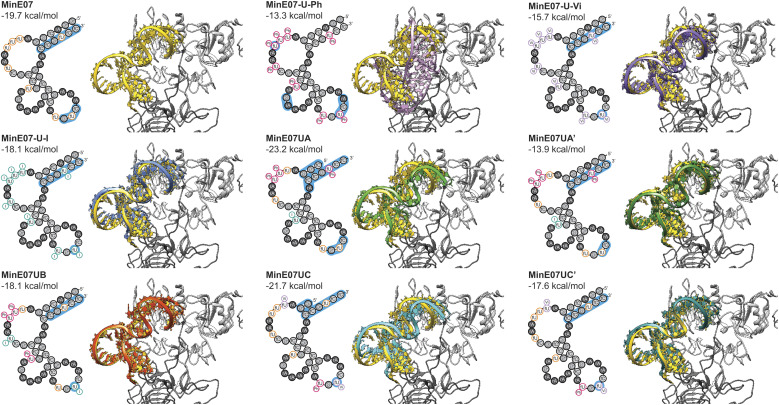
Structures for the selected set of aptamer modifications, and the control aptamers. For each aptamer, the left part of the figure shows the secondary structure including modifications, and the portions which interact with EGFR are highlighted in blue. The right panels show the 3D docked structure, with the aptamers overlaid on the parent EGFR structure (in yellow).

Computational docking studies were performed to provide structural insight into the effect of the selected modifications. The secondary structure of the aptamer was calculated using ViennaRNA 2.0,^54^ and a 3D structure was generated using RNA Composer.^55,56^ Modifications were then manually added using Avogadro, and each strand was energy minimised. Docking was then performed using AutoDockTools.^57,58^ The docking results (Fig. 6) confirm that the FABS selection procedure is essential to this method, and chemically modifying aptamers can improve their affinity for their desired target. Specifically, aptamers MinE07UA and MinE07UC which gave the best *K*_d_ values of all those tested experimentally, also produced the best computational EGFR docking scores, −23.2 and −21.7 kcal mol^−1^. MinE07-U-Ph, MinE07-U-Vi, MinE07-U-I, MinE07UA′ and MinE07UC′ which were not selected from the MinE07Library by FABS, all give worse EGFR docking scores than MinE07.

Our modelling (Fig. 6) shows that the general structure of MinE07 consists of the 5′ and 3′ ends rG(1)rGrAfCrGrG(4)/fC(9)fCrGfUfCfC(12) base pairing to create a duplex. This duplex points into the cavity which binds EGF (the natural ligand) in the crystal structure used as the basis for the modelling. The aptamer sits such that Pro7, Tyr13, Cys20, Tyr22, Asp7, Tyr29, Glu40, Arg41, Glu90, Asn91, Tyr93, Asn151 and Lys28 all hydrogen bond with the phosphate backbone. The rest of the aptamer forms three loops, which do not interact strongly with the protein, until in the third loop rG(9)fUfC(5) associates with the second EGFR in the dimer, interacting with Ser222, Glu233, Glu258 and Lys269. MinE07-U-Ph which has one of the worst *K*_d_ values and docking scores (−13.3 kcal mol^−1^) sits in the binding pocket in a very different orientation, showing that the complete modification hinders the overall binding. MinE07-U-Vi binds in a similar fashion to MinE07 itself, with the vinyl group of fU-Vi(8) slotting between Glu90 and Asn91, while fU-Vi(7) sits between Glu258, Glu221, and Thr235. MinE07-U-I is also similar overall, but with the iodine of fU-I(7) and fU-I(8) both pointing towards Glu residues which could indicate formation of halogen bonds.^36,37^MinE07UA sits further into the binding pocket than MinE07 at the 5′ and 3′ ends that interacts *via* base pairing. This is because of the truncation of three nucleotides and the fU-Ph modification at position U2, U3 and U8 distorting the original base pairing, pulling the stands apart allowing them to go deeper into the binding pocket. Due to shape changes in other areas of MinE07UA caused by chemical modifications, the fU-Ph(7) sits deeper into the binding pocket, with the phenyl ring located in empty space between the side chains of Glu90 and Asn91. The full length analogue MinE07UA′ binds more weakly since the untruncated duplex cannot protrude as deeply into the EGF binding pocket; it overlays very closely with the unmodified aptamer. MinE07UB is very similar to the parent aptamer, but with the additional possibility of a halogen bond being formed between fU-I(7) and Glu258; the docking model does not handle halogen bonds, so this may explain the difference between the calculated docking energy and the measured *K*_d_. Like MinE07UA, MinE07UC also sits further into the binding pocket than MinE07 at the 5′ and 3′ end due to the separation of the original base pairing caused by the truncation of four nucleotides and the modification fU-Vi in U1 position. The fU-Ph at U6 position rotates the molecule slightly allowing the fU-Vi at U7 position to be further into the same binding pocket as MinE07UA, the fU-Vi(7) is predicted to be interacting with Ser222 and Glu258. The phenyl modification at U6 also stops the base pairing that usually occurs in MinE07 between U6 and A12, causing the fU-Ph(6) to sit closer to Ser146, Asp147, Cys195 and Ser196. These extra interactions are lost when the aptamer is in its full-length version.

## Discussion

We have combined a series of techniques which have previously been used separately – phosphoramidite synthesis, one-bead-one-compound libraries, fluorescence-activated sorting, and secondary ion mass spectrometry of oligonucleotides – to create a new method which has successfully identified the exact number, identity, and sequence position of chemical modifications which result in EGFR affinity-optimised aptamers. By comparison with the parent MinE07 aptamer and uniformly modified controls, we have shown that performing this selection makes a valuable difference to the aptamer affinity for EGFR. An improvement factor of 3.3 is enough to make a difference in industrial applications – for example, it could reduce the effective dose of a therapeutic aptamer threefold, which would decrease production costs of an expensive active pharmaceutical ingredient, and potentially circumnavigate side effects. Since our method does not use enzymatic amplification, all the chemical information about the modifications is retained throughout the selection and sequencing workflow, which means that chemical optimization can now be performed orthogonally to the aptamer nucleobase sequence. In doing so, the aptamer can be made more fully complementary to the chemical features of the target, bringing them closer to the versatility of antibodies. This has the potential to convert known, but poorly performing aptamers for important targets into systems which could now be applied in drug discovery.

Current research shows that DNA and RNA aptamers have median *K*_d_ values of 32.8 nM and 19.7 nM respectively.^23^ The method of modifying nucleic acids reported here has produced aptamers in the range of 1–3 nM, and gave an improvement in affinity of up to 3.3-fold, outperforming even recent *de novo*-selected uniformly modified aptamers.^59–62^ The experimental validation using the EGFR binding assay has been backed up with computational modelling, which provides a structural basis for the changes in affinity and could be used to further refine the aptamers. A typical SELEX process takes 2–3 months for selection and sequence identification.^21^ After the development of the methods described herein, it is possible to synthesise an aptamer library in one week, perform FABS in one day, followed by four weeks of sequence analysis, (for which RoboOligo is invaluable). This novel method could improve the success of aptamer discovery processes and general drug discovery process, making them more cost and time effective. Outside of the aptamer field, this method has the potential to impact drug discovery processes with this new method of synthesising and screening large drug candidate libraries rapidly. Nonetheless, there is room for fine-tuning of SOLFABS. Currently, the bottleneck is the sequencing step; in future iterations better MS/MS data could be obtained by optimising synthetic conditions or solid support, or by use of more advanced mass spectrometry.

## Conclusion

In summary, we herein report a new method for the identification of chemical modifications at specific locations on an aptamer which improve its target binding affinity, using a high-precision automated selection process. This has resulted in improvement in affinity of up to 3.3-fold, boosting these aptamers further above the median binding strength of RNA aptamers. We anticipate that this process could be a step-change for use of aptamers in therapeutic and analytical applications by making them competitive with antibodies not just in shelf-life, synthesis, and pharmacokinetics, but also in affinity, their single most defining feature.

## Experimental

Full experimental details and additional supporting data can be found in the ESI.† These data concern bead suitability, monomer synthesis, library synthesis, FABS, MS/MS, aptamer resynthesis, validation, and computational aspects.

## Data availability

Raw data for all experiments is available upon request from the authors.

## Author contributions

A. R. P. performed all practical experiments, data analysis and interpretation. M. F. performed all computation modelling and docking studies. The project was conceived by M. D. G. and C. J. S. H. L. helped design the protein binding affinity assay experiments. A. R. P. wrote the manuscript. C. J. S. and M. D. G. edited the manuscript. All experiments were conducted under the supervision of C. J. S. and M. D. G. All authors read and approved the final manuscript.

## Conflicts of interest

There are no conflicts to declare.

## Supplementary Material

SC-014-D3SC03581F-s001
