# Supplementary material for: Selection of optimised ligands by fluorescence-activated bead sorting
Source: Chem Sci. 2023 Aug 11;14(35):9517–25. doi: 10.1039/d3sc03581f (PMC10498682; doi:10.1039/d3sc03581f)
Supplement: SC-014-D3SC03581F-s001 [file SC-014-D3SC03581F-s001.pdf]

Electronic Supplementary Material (ESI) for Chemical Science.
This journal is © The Royal Society of Chemistry 2023

Selection of Optimised Ligands by Fluorescence-Activated Bead Sorting

Alexandra R. Paul,[a†] Mario Falsaperna,[a‡] Helen Lavender,[b] Michelle D. Garrett,[c*] Christopher J. Serpell[a,d*]

[a] School of Chemistry and Forensic Sciences, Division of Natural Sciences, University of Kent, Canterbury, CT2 7NH, UK. [b] Avvinity Therapeutics, 66 Prescot Street, London, E1 8NN. [c] School of Biosciences, Division of Natural Sciences, University of Kent, Canterbury, CT2 7NJ. [d] School of Pharmacy, University College London, London, WC1N 1AX, UK. * to whom correspondence should be addressed.

Corresponding Authors

* M.D.Garrett@kent.ac.uk; chris.serpell@ucl.ac.uk

Present Addresses

† Institute of Pharmaceutical Science, Faculty of Life Sciences & Medicine, King's College London, London SE19NH, UK.
‡ Department of Chemistry, University of Bath, Bath, BA2 7AY, UK.

Supplementary Information

Contents

S1. General considerations 2

S2. Bead suitability 4

S3. Synthesis of monomers 6

S4. Aptamer library synthesis 30

S5. Fluorescence-activated bead sorting 32

S6. Sequencing by tandem mass spectrometry 33

S7. Aptamer resynthesis 75

S8. Aptamer validation 84

S9. Computational docking studies 88

S10. References 89

S1. General considerations

Reagents. TentaGel® M NH_2 Monosized Amino TentaGel Microspheres (TG-beads) were purchased from Rapp Polymere.[1] Sheath Fluid, 8 peak Rainbow calibration beads and Accudrop beads was purchased from BDBiosciences. 2'-fluoro-2'-deoxyuridine, 4,5-dicyanoimadazole, acetic anhydride, ammonia methanol, ammonium cerium (IV)nitrate, caesium carbonate, dioxane, nitrosonium tetrafluoroborate, tetrakistriphenylphophine palladium (0) and tetravinyl tin were purchased from Acros Organic. 4-(aminomethyl)phenylboronic acid pinacol ester, 4-carboxyphenylboronic acid pinacol ester, ammonia solution, sodium bicarbonate and sodium sulfate were purchased from Alfa Aesar. [3-(2-aminoethylamino)propyl]trimethoxysilane], 6-carboxyfluroescein, 10-hydroxydecanoic acid, boric acid, dichloromethane, diethyl ether, dimethylformide, EDTA, ethanol, ethyl acetate, KCN, nbutanol, N,N'-dissopropylcarbodiimide, magnesium sulfate, methanol, ninhydrin, rhodamine B, phenol, pyridine, sodium chloride, triethylamine , tris base were purchased from Thermo Fisher Scientific. 1,1'bis(diphenylphosphineo)ferrocenepalladium(II) dichloride, 4,4'-dimethoxytrityl chloride, diisoproplethylamine, (4-(4,4,5,5-tetramethyl-1,3,2-dioxaborolan-2-yl)phenyl)methanamine hydrochloride, 4-dimethylaminopyridine and palladium acetate were purchased from Fluorochem. 2-cyanoethyl-diisopropl-chlorophosphoramidite was purchased from Link Technologies. Bovine Serum Albumin, fluorenylmethoxycarbonyl, iodine, nuclease free water, phenylboric acid pinacol ester, triphenylphosphine, salmon sperm DNA (ssDNA), PBS with calcium and magnesium ($PBS^{(+/+)}$), PBS without calcium and magnesium ($PBS^{(-/-)}$), tween-20 and streptavidin–alkaline phosphatase (streptavidin–AP) from *streptomyces avidinii,* methanol hypergrade for LC-MS LiChrosolv®, Water for chromatography (LC-MS Grade) LiChrosolv® and MicroSpin™ G-50 columns were purchased from Sigma Aldrich/MERCK. All NMR samples were run in DMSO-d_6 purchased from Goss Scientific. Recombinant Human EGFR Fc Chimera Protein, CF was purchased from R&D Systems. Native *Staphylococcus aureus* Protein A (FITC) (ab7455) was purchased from Abcam. Tropix CDP-Star Ready to use with Sapphire II and White PS – Protein A coated –level 200 µL 96 well plates were purchased from Biomat. Costar assay 96 well plates (with lid, white, flat bottom, tissue culture treated) were purchased from Thermo Fisher Scientific. Mass spectrometry columns nanoE MZ Sym C18 Trap Column 5 µm and nanoE MZ HSS T3 Column 1.8 µm 75 µm x 150 mm were purchased from Waters. MinE07- Biotin was purchased from Integrated DNA Technologies.

Synthesiser reagents. Oxidizer (0.02M iodine, 20% pyridine), Cap A Mix (THF/Pyridine/acetic anhydride 8:1:1), Cap B Mix (10% methylimidazole in THF), deblock (3% trimethylamine in DCM) and ETT activator solution (0.25 M, 5-ethylthio-1H-tetrazole in acetonitrile) were purchased from Link Technologies. N2-acetyl-2'-O-tert-butyldimethylsilyl-5'-O-DMT-guanosine 3'-CE phosphoramidite, N6-benzoyl-2'-O-tert-butyldimethylsilyl-5'-O-DMT-adenosine 3'-CE phosphoramidite, N4-acetyl-2'-deoxy-5'-O-DMT-2'-fluorocytidine 3'-CE phosphoramidite and 2'-deoxy-5'-O-DMT-2'-fluorouridine 3'-CE phosphoramidite were purchased from CarboSynth.

Buffers. 10 x tris-borate buffer (TBE): tris Base (108 g), boric Acid (55 g), EDTA (7.45 g), dH_2O to 1 L at 8.3 pH. 10 x TAMg buffer (TAMg): tris base (54.51 g), $Mg(OAc)_2.4H_2O$ (26.8 g) dH_2O to 1 L at 7.8 pH.
Wash Buffer: PBS + 0.05% Tween 20, to 500 mL of PBS $^{(+/+)}$, add 250 µL of Tween-20. Protein Binding Buffer: 48.5 mL of PBS $^{(+/+)}$, 1 mL 50 mg/mL BSA and 0.5 mL 10 mg/mL ssDNA were added.

Instrumentation

Fluorescent Activated Cell Sorter: Flow cytometry data was collected on a BD FACSJazz™ Cell Sorter by Becton Dickinson. All flow cytometry data was processed using BD FACS™ Sortware sorter software. 8 peak Rainbow Calibration beads were already run at the start of all analysis or sorting experiments. For sorting experiments Accudrop beads were used for calibration. The drop-delay value was adjusted while viewing BD FACS Accudrop beads in the centre and side sort streams that are illuminated by a red laser. Sort monitoring was undertaken with live video feed of breakoff point, waste collection, and side streams. The Accudrop beads were sorted into two gates.

Fluorescence Microscopy: Leica DMR microscope equipped with a Leica DFC9000 GT digital camera. The illumination source was a CoolLED pE-300^{ultra} fluorescence microscopy Illumination System. Images were

acquired using Leica Application Suite X software. Excitation 495nm and emission 525/50 nm. Excitation 515-560 nm and emission long pass 560-590 nm.

Oligonucleotide Synthesiser: All library components were synthesised on an Expedite™ 8909 Nucleic Acid Synthesiser system provided by Biolytic. Phosphoramidites were dissolved in dry acetonitrile to the concentrations as suggested by the supplier, solvents were used as provided. Oligomers were synthesised on a 1 µM scale.

Mass Spectrometer (Small Molecule): Electrospray mass spectra were recorded on a Bruker micrOTOF-Q II mass spectrometer. The samples were analysed by injecting 2 µL of 0.1 mg/mL solutions into a flowing stream of 95% methanol, 10 mM ammonium acetate at a flow rate of 20 µL/min. The samples were analysed in both positive and negative ion modes. All the masses are mono isotopic and lock mass corrected and therefore should be within 5 ppm of the calculated mass.

Mass Spectrometer (Oligonucleotides): Mass spectra were recorded on Waters QToF ESI-LC/MS/MS. Using columns nanoE MZ Sym C18 Trap Column 5 µm and nanoE MZ HSS T3 Column 1.8 µm 75 µm x 150 mm. The samples were analysed by injecting 2 µL of 0.1 mg/mL solutions into a flowing stream of 95% methanol, 10 mM ammonium acetate at a flow rate of 20 µL/min. The samples were analysed in both positive and negative ion modes. Mobile phase A was 8 mM tetraethyl ammonium bromide (TEAB) in LC-MS grade water adjusted to the pH 7.5-7.8. Mobile phase B was 8 mM TEAB in a 1:1 ratio of LC-MS grade methanol and water. Initially A was held at 92% and B at 8%. At 30 mins B was increased to 65% and up to 95% at 31 mins. At 33 mins B was dropped back to 8% and then held until 60 mins. The column is maintained at 50˚C.[2,3]

NMR: NMR spectra were obtained on a Bruker AVII 400 MHz spectrometer and were calibrated to the centre of the set solvent peak and chemical shifts were reported in parts per million (ppm). All NMR samples were run in DMSO-d_6.

Scanning Election Microscope: SEM images were obtained on a Hitachi S-3400N, using a 10 kV electron beam and secondary electron detector.

S2 Bead suitability

Imaging of TentaGel® M NH_2 Monosized Amino TentaGel Microspheres using SEM. SEM images were obtained on a Hitachi S3400N, using a 10 kV electron beam and secondary electron detector. The sample was deposited on carbon tabs placed on aluminium sample holders. The images were visualised on ImageJ and the data analysis was done using Origin Lab software.

Amidation of 10-hydroxydecanoic acid with TentaGel® M NH_2 Monosized Amino TentaGel Microspheres. *N*,*N′*-Dissopropylcarbodiimide (0.2 g) was dissolved in dimethylformamide (10 mL). 10-Hydroxydecanoic Acid (0.4 g) was added to the solution. TG-beads (0.1 g) were swelled in dimethylformamide (10 mL) and then added to the solution. This solution was stirred for three hours at room temperature. It was spun down and cleaned with dimethylformamide (10 mL x 3) and then with ethanol (10 mL x 2). A white power was produced (0.95 g, 95%). A Kaiser test was conducted producing a yellow colour (successful).

Fluorescent tagging of TentaGel® M NH_2 Monosized Amino TentaGel Microspheres. 6-Carboxyfluorescein. *N*,*N*′-Disopropylcarbodiimide (0.32 g) was dissolved in dimethylformamide (10 mL). The amine-modified TG-beads (0.05 g) were swelled in dimethylformamide (10 mL), this was added to the solution along with 6-carboxyfluroescein (0.05 g). The solution was stirred for 3 hours at room temperature. It was spun down and cleaned with dimethylformamide (10 mL x 3) and then with ethanol (10 mL x 2). The product was then air dried and an orange powered was collected (TGCFluor100) (0.074 g, 74%). Samples TGCFluor100, TGCFluor73 and TGCFluor36 are made per amounts in table.

Table 1. Amount of 6-Carboxyfluorscein for samples TGCFluor100/73/36

Experiment	Percentage of 6-Carboxyfluorescein	Amount of 6-Carboxyfluorscein
TGCFluor100	100%	0.050 g
TGCFluor73	73%	0.025 g
TGCFluor36	36%	0.013 g

Rhodamine B. *N,N′*-Dissopropylcarbodiimide (0.15 g) was dissolved in dimethylformamide (10 mL). The amine modified TG-beads (0.05 g) were swelled in dimethylformamide (10 mL), this was added to the solution along with Rhodamine B (0.05 g). The solution was stirred for 3 hours at room temperature. It was spun down and cleaned with dimethylformamide (10 mL x 3) and then with ethanol (10 mL x 2). The product was then air dried and a pink powered was collected (TGRhodB100) (0.03 g, 60%). Samples TGRhodB100, TGRhodB73, TGRhodB36, TGRhodB17 an TGRhodB1.7 are made per amounts in table.

Table 2. Amount of Rhodamine B for samples TGRhod100/73/36/17/1.7

Experiment	Percentage of Rhodamine B	Amount of Rhodamine B
TGRhodB100	100%	0.050 g
TGRhodB73	73%	0.025 g
TGRhodB36	36%	0.013 g
TGRhodB17	17%	0.005 g
TGRhodB1.7	1.7%	0.0005 g

6-carboxyfluorescein tagged TentaGel® M NH_2 Monosized Amino TentaGel Microspheres on the fluorescent microscope. Leica DMR microscope equipped with a Leica DFC9000 GT digital camera. The illumination source was a CoolLED pE-300^{ultra} fluorescence microscopy Illumination System. Images were

acquired using Leica Application Suite X software. Excitation 495nm and emission 525/50 nm. Excitation 515-560 nm and emission long pass 560-590 nm. A small sample of 6-carboxyfluorescein tagged TG-beads in water were smeared onto a glass slide. This was placed on the microscope platform and visualised using a fluorescent lamp.

Flow Cytometry analysis of tagged TentaGel® M NH_2 Monosized Amino TentaGel Microspheres. 6-Carboxyfluorescein. Flow cytometry was performed on the tagged TentaGel® beads (TG-beads) using a BD FACSJazz™ Cell Sorter. The FACSJazz lasers were calibrated before the samples were run with the Rainbow Calibration Particles, 8 peaks (3.0-3.4 µm). Non-labelled TG-beads were dispersed in 5 mL sheath fluid and put through the machine to check the calibration. Then samples TGCFluor100, TGCFluor73 and TGCFluor36 were run through the FACS dispersed in 5 mL sheath fluid, being observed on laser 488 nm with filter 513/17 nm. All flow cytometry data was processed using BD FACS™ Sortware program.

Rhodamine B. The FACSJazz lasers were calibrated before the samples were run with the BD Rainbow Calibration Particles (8 peaks). Non-labelled TG-beads were dispersed in 5 mL sheath fluid and put through the machine to check the calibration. Then samples TGRhodB100, TGRhodB73, TGRhodB36, TGRhodB17 and TGRhodB1.7 were run through the FACS dispersed in 5 mL sheath fluid, being observed on laser 488 nm with filter 585/29 nm.

96-well plate sort of 100% 6-carboxyfluorescein tagged microspheres. The FACS was calibrated using the 96-well plate set up program along with BD Accudrop beads. 6-carboxyfluorescein tagged TG-beads (2 mL) were put into a FACS sized falcon tube. 1 gate was created for the population of TG-beads. The TG-beads were sorted into wells B1-9, C1-9, D1-9 in a ½ log digression starting at 50,000 microspheres down to 5 microspheres. The 488 nm laser was used with filter 513/17 nm. All data was collected and analysed using the BD FACSJazz Sortware sorter program. The 96 well plate samples were then read on the Victor X4 plate reader (Fluorescein (485 nm/535 nm, 1.0 s).

S3 Synthesis of monomers

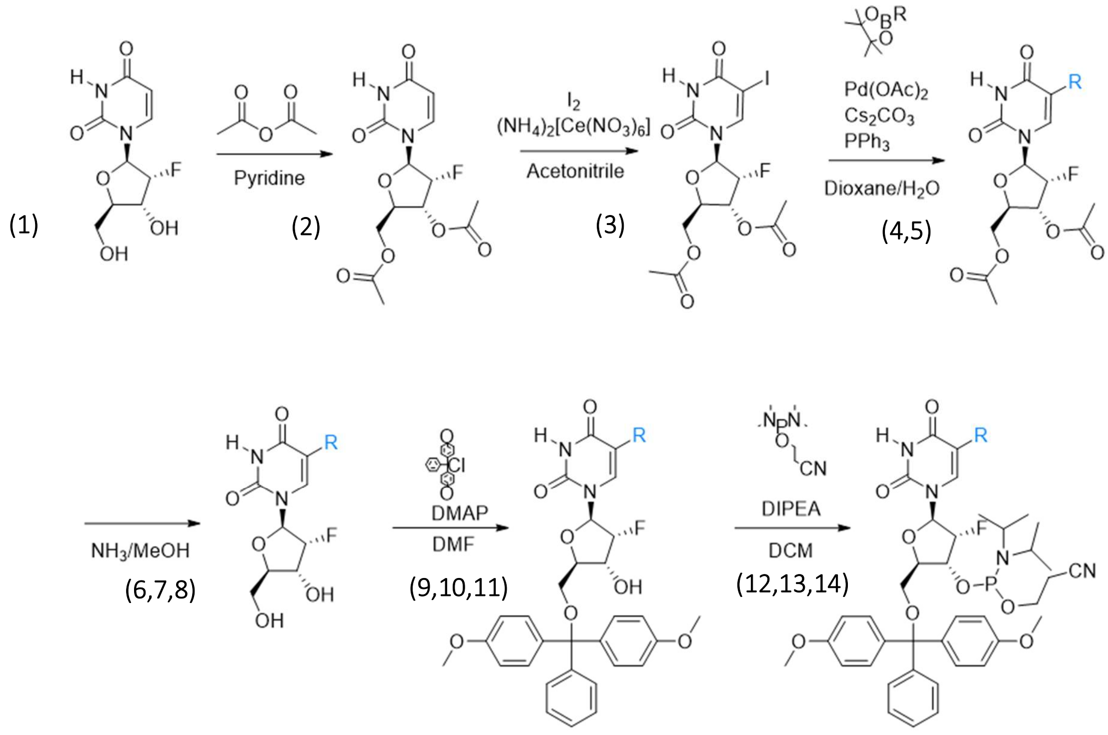

Figure 1. General reaction scheme for the synthesis of modified uridine.

Step 1: Acetyl protection of 2'-fluoro-2'-deoxyuridine.[4]

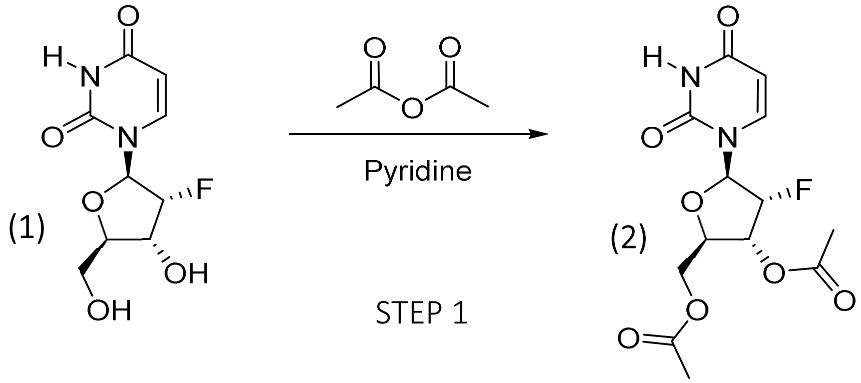

Figure 2. Reaction scheme for acetyl protection of 2'-Fluoro-2'-deoxyuridine.

Acetyl protection of 2'-fluoro-2'-deoxyuridine.[4] 2'-fluoro-2'-deoxyuridine (**1**) (3 g, 12.185 mmol) was dissolved in pyridine (150 mL) and then cooled to 0°C. Acetic anhydride (12.39 g, 121.364 mmol) was added, and the reaction was stirred for 1 hour at 0°C, then for an additional hour at room temperature. The reaction was monitored by TLC (Ethyl Acetate/MeOH 20:1). The solvent was then removed in vacuo. The residue was then dissolved in methanol (100 mL) and the solvent was removed *in vacuo* again. A white powdered solid was collected, 2'-Fluoro-3',5'-di-O-acetyl-2'-deoxyuridine (**2**) (3.82 g, 98.7%). NMR spectra were obtained on a Bruker AVII 400 MHz spectrometer and were calibrated to the centre of the set solvent peak and chemical shifts were reported in parts per million (ppm).^{1}H NMR (DMSO-d$_6$): δ 11.47 (s, 1H); 7.71-7.73 (d, 1H); 5.90 (dd, 1H); 5.68-79 (dd, 1H); 5.62 (ddd, 1H); 5.24-5.48 (ddd, 1H); 4.33 (dd, 1H); 4.24 (ddd, 1H); 4.12 (dd,1H); 2.12 (s, 3H); 2.04 (s, 3H). MS: m/z: calcd for $C_{13}H_{15}FN_2O_7$ ([M + Na]$^+$): 353.09, found 352.70

Step 2: Iodination of 2'-fluoro-3',5'-di-O-acetyl-2'-deoxyuridine.[4]

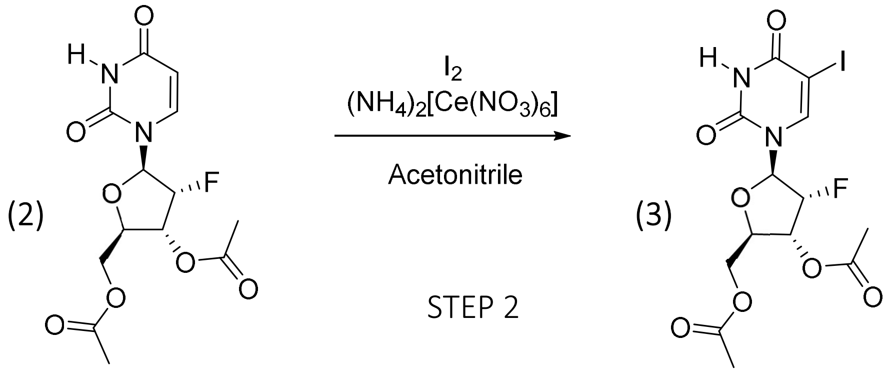

Figure 3. Reaction scheme for iodination of 2'-fluoro-3',5'-di-o-acetyl-2'-deoxyuridine.

Iodination of 2'-fluoro-3',5'-di-O-acetyl-2'-deoxyuridine.[4] **2** (1.5 g) was dissolved in acetonitrile (100 mL). After addition of iodine (1.14 g, 8.98 mmol) and $(NH_4)_2[Ce(NO_3)_6]$ (2.49 g, 5.32 mmol) the reaction was heated to 95°C and stirred for 3hours under reflux. The reaction was monitored by TLC (Ethyl Acetate/MeOH 20:1). The solvent was removed *in vacuo* and a silica column was run (Ethyl Acetate/MeOH 20:1) to product an orange oil 3',5'-di-O-acetyl-2'-desoxy-2'-fluoro-5-iodo-uridine (**3**) (1.512 g, 73.04%). ^{1}H NMR (DMSO-d$_6$): δ 11.87 (s, 1H); 8.20 (s, 1H); 5.84-90 (dd, 1H); 5.46-61 (ddd, 1H); 5.23-31 (ddd, 1H); 4.33-37 (dd, 1H); 4.27-31 (ddd, 1H); 4.17-21 (dd,1H); 2.11 (s, 3H); 2.08 (s, 3H). MS: m/z: calcd for $C_{13} H_{14} FIN_2 O_7$ ($[M + H]^+$): 456.98, found 456.9 ($[M + Na]^+$): 479.98, found 479.8.

Step 3U-Ph: Addition of functional group Ph to 3',5'-di-O-acetyl-2'-desoxy-2'-fluoro-5-iodo-uridine using Suzuki-Miyaura cross coupling reaction.[4]

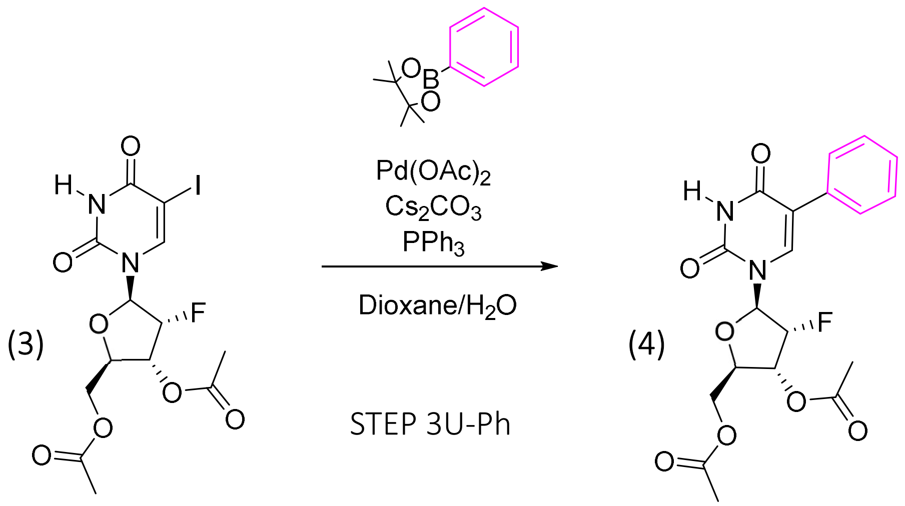

Figure 4. Addition of functional group a to 3',5'-di-O-acetyl-2'-desoxy-2'-fluoro-5-iodo-uridine using Suzuki-Miyaura cross coupling reaction.

Addition of functional group U-Ph to 3',5'-di-O-acetyl-2'-desoxy-2'-fluoro-5-iodo-uridine using Suzuki-Miyaura cross coupling reaction.[5] To a solution of **3** (1.51 g), Cs_2CO_3 (2.39 g, 7.35 mmol), phenylboronic acid pinacol ester (0.63 g, 3.11 mmol) and PPh_3 (0.30 g, 1.15 mmol) in dioxane (50 mL) and water (4.5 mL) was added to $Pd(OAc)_2$ (0.63 g, 2.82 mmol) at 25°C under N_2 current. The mixture was refluxed at 90°C and stirred for 16 hours. The reaction was monitored by TLC (ethyl acetate/MeOH = 20:1). The black palladium precipitate was filtered off. The reaction mixture was diluted with water (10 mL) and extracted with ethyl acetate (20 mL x 2). The organic layers were then washed with saturated aqueous NaCl (10 mL), dried over $MgSO_4$ and filtered. This was then concentrated under reduced pressure to give a residue. 3',5'-di-O-acetyl-

2'-desoxy-2'-fluoro-5-phenyl-uridine was produced as a sticky yellow oil (**4**) (1.53 g, 87.529%). ^{1}H NMR (DMSO-d$_6$): δ 11.71 (s, 1H); 7.94 (s, 1H); 7.32-7.74 (m, 5H); 5.86-6.01 (dd, 1H); 5.48-69 (ddd, 1H); 5.28-36 (ddd, 1H); 4.33-38 (dd, 1H); 4.26-31 (ddd, 1H); 4.16-21 (dd,1H); 2.13 (s, 3H); 2.08 (s, 3H). MS: m/z: calcd for $C_{19}H_{19}FN_2O_7$ ([M + Na]$^+$): 429.12, found 429.0

Step 3U-Vi: Addition of functional group Vi to 3',5'-di-O-acetyl-2'-desoxy-2'-fluoro-5-iodo-uridine using Stille cross coupling reaction.[6]

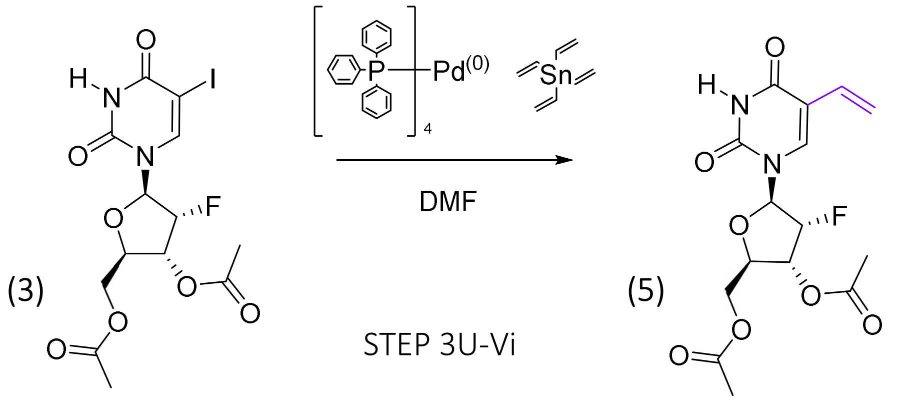

Figure 5. Addition of Functional Group U-Vi to 3',5'-di-O-acetyl-2'-desoxy-2'-fluoro-5-iodo-uridine using Stille Cross Coupling Reaction.

Addition of functional group U-Vi to 3',5'-di-O-acetyl-2'-desoxy-2'-fluoro-5-iodo-uridine using Stille cross coupling reaction.[6] **3** (1 g), tetravinyltin (0.49 mL, 1.15 mmol) and tetrakistriphenylphosphinepalladium(0) (0.19 g, 0.16 mmol) in 15 mL of DMF was stirred under nitrogen at 75°C for 17hours under N_2 current. The reaction was monitored by TLC (DCM/MeOH = 10:1). The black palladium precipitate was filtered off. The reaction mixture was diluted with water (25 mL x 2) and extracted with diethyl ether (25 mL x 2). The diethyl layers were combined and dried over $MgSO_4$, filtered, and concentrated under reduced pressure to give a residue. 3',5'-di-O-acetyl-2'-desoxy-2'-fluoro-5-vinyl-uridine was produced as a yellow oil (**5**) (0.37 g, 35.02%). ^{1}H NMR (DMSO-d$_6$): δ 11.62 (s, 1H); 7.88 (s, 1H); 6.33-41 (dd,1H); 5.85-99 (q, 1H); 5.50-65 (dd, 1H) 5.25-5.33 (m, 2H); 5.17-5.19 (dd, 1H); 4.33-37 (dd, 1H); 4.28-31 (ddd, 1H); 4.17-22 (dd,1H); 2.11 (s, 3H); 2.03 (s, 3H). MS: m/z: calcd for $C_{15}H_{17}FN_2O_7$ ([M + Na]$^+$): 379.10, found 379.0

Step 4U-Ph/Vi/I: Removal of acetyl protecting groups from 3',5'-di-O-acetyl-2'-desoxy-2'-fluoro-5-phenyl-uridine, 3',5'-di-O-acetyl-2'-desoxy-2'-fluoro-5-vinyl-uridine and 3',5'-di-O-acetyl-2'-desoxy-2'-fluoro-5-iodo-uridine.

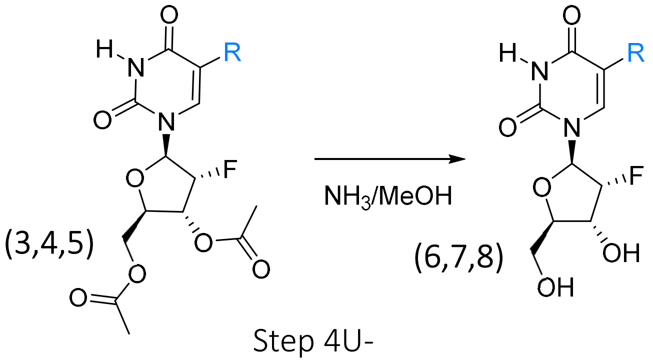

Figure 6. Step 4U-Ph/Vi/I: Removal of acetyl protecting groups from 3',5'-di-O-acetyl-2'-desoxy-2'-fluoro-5-phenyl-uridine, 3',5'-di-O-acetyl-2'-desoxy-2'-fluoro-5-vinyl-uridine and 3',5'-di-O-acetyl-2'-desoxy-2'-fluoro-5-iodo-uridine.

Removal of acetyl protecting groups from 3',5'-di-O-acetyl-2'-desoxy-2'-fluoro-5-phenyl-uridine. 4 (1.53 g) was dissolved in an NH_3/MeOH solution (7N, 15 mL) and stirred at room temperature for 20 hours. The reaction was monitored by TLC (Ethyl Acetate/MeOH 20:1). The solvent was removed in vacuo and a solid of 2'-desoxy-2'-fluoro-5-phenyl-uridine was collected (**6**) (0.98 g, 81.13%). ^{1}H NMR (DMSO-d_6): δ 11.71 (s, 1H); 7.94 (s, 1H); 7.32-7.74 (m, 5H); 5.86-6.01 (dd, 1H); 5.48-69 (ddd, 1H); 5.28-36 (ddd, 1H); 4.33-38 (dd, 1H); 4.26-31 (ddd, 1H); 4.16-21 (dd,1H). MS: m/z: calcd for $C_{19}H_{19}FN_2O_7$ ([M + Na]$^+$): 345.10, found 345.0

Removal of acetyl protecting groups from 3',5'-di-O-acetyl-2'-desoxy-2'-fluoro-5-vinyl-uridine. 5 (0.37 g) was dissolved in an NH_3/MeOH solution (7N, 15 mL) and stirred at room temperature for 20 hours. The reaction was monitored by TLC (Ethyl Acetate/MeOH 20:1). The solvent was removed in vacuo and a solid of 2'-desoxy-2'-fluoro-5-vinyl-uridine was collected (**7**) (0.25 g, 92.59%). ^{1}H NMR (DMSO-d_6): δ 11.53 (s, 1H); 8.28 (s, 1H); 6.36-39 (d, 1H); 6.30-32 (d, 1H); 5.92-94 (t, 1H); 5.28-36 (t, 1H); 5.42-65 (dd, 1H); 5.11-14 (q, 1H); 4.99 (s,1H); 4.20-30 (dd,1H); 3.82-89 (t,1H); 3.64-74 (t,1H); 3.61 (s,1H). MS: m/z: calcd for $C_{11}H_{13}FN_2O_5$ ([M + H]$^+$): 272.08, found 272.91.

Removal of acetyl protecting groups from 3',5'-di-O-acetyl-2'-desoxy-2'-fluoro-5-iodo-uridine. 3 (1.53 g) was dissolved in an NH_3/MeOH solution (7N, 15 mL) and stirred at room temperature for 20 hours. The reaction was monitored by TLC (Ethyl Acetate/MeOH 20:1). The solvent was removed in vacuo and a solid of 2'-desoxy-2'-fluoro-5-iodo-uridine was collected (**8**) (1.21 g, 96.80%). ^{1}H NMR (DMSO-d_6): δ 11.75 (s, 1H); 8.55 (s, 1H); 5.83-5.87 (d, 1H); 5.63-5.64 (t, 1H); 5.43 (s, 1H); 4.97-5.11 (dd, 1H); 4.14-4.18 (dd, 1H); 3.88-3.90 (t, 1H); 3.81-3.83 (t,1H); 3.58 (s,1H). MS: m/z: calcd for $C_9H_{10}FN_2O_5$ ([M + H]$^+$): 372.96, found 373.90. ([M + Na]$^+$): 395.10, found 394.85.

Step 5U-Ph/Vi/I: Addition of 4,4'-dimethoxytrityl (DMT) protecting group to 2'-desoxy-2'-fluoro-5-phenyl-uridine, 2'-desoxy-2'-fluoro-5-vinyl-uridine[7] and 2'-desoxy-2'-fluoro-5-iodo-uridine.

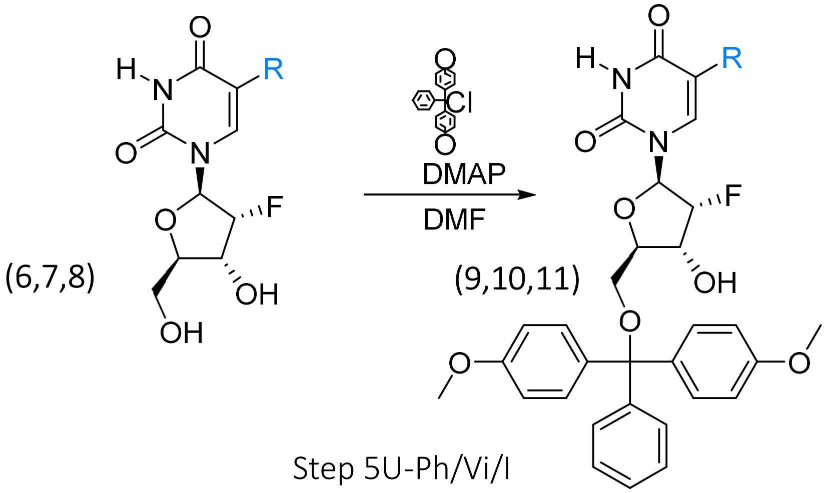

Figure 7. Step 5U-Ph/Vi/I: Addition of 4,4'-dimethoxytrityl (DMT) protecting group to 2'-desoxy-2'-fluoro-5-phenyl-uridine, 2'-desoxy-2'-fluoro-5-vinyl-uridine and 2'-desoxy-2'-fluoro-5-iodo-uridine.

Addition of 4,4'-dimethoxytrityl protecting group to 2'-desoxy-2'-fluoro-5-phenyl-uridine. 6 (0.064 g) and DMAP (0.0013 g, 0.011 mmol) were dissolved in pyridine (10 mL). 4,4'-Dimethoxytrityl chloride (0.077 g, 0.23 mmol) was added in portions over 1 hour dropwise at 0°C. After complete consumption of the starting material (18 hours), MeOH (2 mL) was added, and the resulting mixture was concentrated under vacuum. The product was purified by column chromatography using a basic alumina stationary phase and eluted with

DCM/MeOH (100:1) to produce a yellow oil (**9**) (0.14 g, 185.95%). ^{1}H NMR (DMSO-d$_6$): δ 9.89 (s, 1H); 8.19 (s, 1H); 7.27-7.31 (m, 3H); 7.19-7.23 (m, 9H); 7.06-7.10 (m, 2H) 6.84-6.89 (m, 4H); 6.22 (s, 1H); 3.91 (q, 1H); 3.87 (t, 1H); 43.66 (s, 1H); 3.55 (d, 1H); 3.07-3.07 (dd,2H). MS: m/z: calcd for $C_{36}H_{33}FN_2O_7$ ([M + Na]$^+$): 647.23, found 647.20

Addition of 4,4'-dimethoxytrityl protecting group to 2'-desoxy-2'-fluoro-5-vinyl-uridine. 7 (0.067 g) and DMAP (0.0013 g, 0.011 mmol) were dissolved in pyridine (10 mL). DMTCl (0.13 g, 0.40 mmol) was added in portions over 1 hour dropwise at 0°c. After complete consumption of the starting material (18 hours), MeOH (2 mL) was added, and the resulting mixture was concentrated under vacuum. The product was purified by column chromatography using a basic alumina stationary phase and eluted with DCM/MeOH (100:1) to produce a yellow oil (**10**) (0.197 g). ^{1}H NMR (DMSO-d$_6$): δ 11.56 (s, 1H); 8.31 (s, 1H); 7.25-7.36 (m, 11H); 6.86-6.88 (m, 4H); 6.36-6.41 (d, 1H); 6.30-6.33 (t, 1H); 5.72-5.96 (dd, 2H); 5.49-5.50 (q, 1H); 5.12-5.13 (t,1H); 5.01 (s,1H); 4.12-4.22 (dd,1H); 3.86-3.92 (dd, 2H); 3.72 (s, 6H). MS: m/z: calcd for $C_{32}H_{31}FN_2O_7$ ([M + H]$^+$): 574.21 , found 574.00.

Addition of 4,4'-dimethoxytrityl protecting group to 2'-desoxy-2'-fluoro-5-iodo-uridine. 8 (0.12 g) and DMAP (0.024 g, 0.019 mmol) were dissolved in pyridine (10 mL). 4,4'-Dimethoxytrityl chloride (0.2359 g, 0.696 mmol) was added in portions over 1 hour dropwise at 0°C. After complete consumption of the starting material (18 hours), MeOH (2 mL) was added and the resulting mixture was concentrated under vacuum. The product was purified by column chromatography using a basic alumina stationary phase and eluted with DCM/MeOH (100:1) to produce a yellow oil was (**11**) (0.333 g). ^{1}H NMR (DMSO-d$_6$): δ 11.79 (s, 1H); 8.58 (d, 1H); 7.08-7.37 (m, 11H); 6.84-6.91 (m,4H); 5.83-5.90 (d, 1H); 5.45-5.67 (dd,1H); 4.99-5.24 (t, 1H); 4.38 (s, 1H); 4.25-4.35 (dd, 1H); 3.93 (t, 1H); 3.74 (s, 6H); 3.64 (d, 1H). MS: m/z: calcd for $C_{30}H_{28}FN_2O_7$ ([M + H]$^+$): 697.09, found 697.00.

Step 6U-Ph/Vi/I: Addition of diisopropylchlorophosphoramidite to DMT protected 2'-desoxy-2'-fluoro-5-phenyl-uridine, 2'-desoxy-2'-fluoro-5-vinyl-uridine[7] and 2'-desoxy-2'-fluoro-5-iodo-uridine.

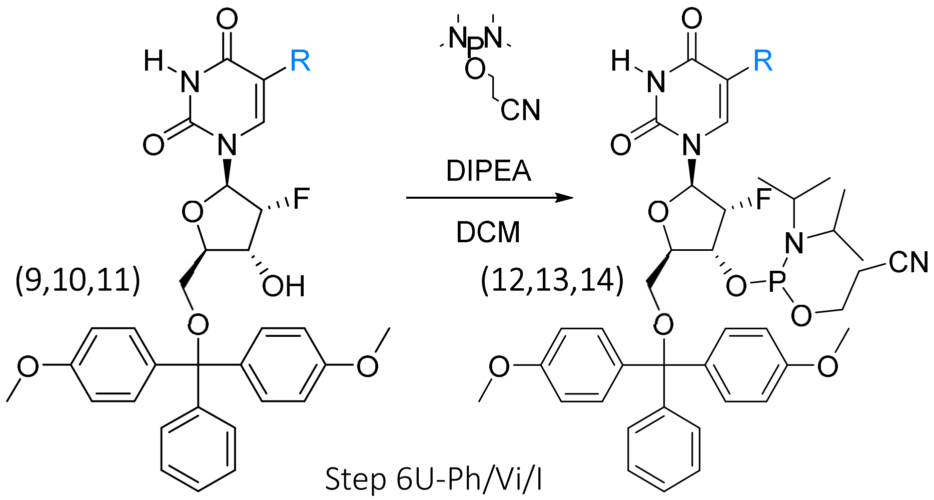

Figure 8. Step 6**U-Ph/Vi/I**: Addition of diisopropylchlorophosphoramidite to DMT protected 2'-desoxy-2'-fluoro-5-phenyl-uridine, 2'-desoxy-2'-fluoro-5-vinyl-uridine and 2'-desoxy-2'-fluoro-5-iodo-uridine.

Addition of 2-cyanoethyl diisopropyl chlorophosphoramidite to DMT protected 2'-desoxy-2'-fluoro-5-phenyl-uridine. 9 (0.31 g), and dimethylaminopyridine (0.05 g, 0.41 mmol) were dissolved in 50 mL of dry DCM, diisopropylethylamine (0.30 mL, 2.33 mmol) and 2-cyanoethyl-diisopropyl-chlorophosphoramidite (0.30 mL, 1.25 mmol) were added. Under an atmosphere of nitrogen, the mixture was then stirred at room temperature for 2 hours. After the DCM was removed *in vacuo* a thick yellow oil was obtained (**12**) (0.60 g). ^{31}P NMR was run on the product, to prove the addition of the phosphoramidite group. No other analysis was

run on it because of the air sensitive nature of the product. ^{31}P NMR (DMSO-d_6 capillary in DCM): δ 150.00 (s, 1P) (0.77:0.23).

Addition of 2-cyanoethyl diisopropyl chlorophosphoramidite to DMT protected 2'-desoxy-2'-fluoro-5-vinyl-uridine. 10 (0.88 g), and dimethylaminopyridine (0.14 g, 1.14 mmol) were dissolved in 50 mL of dry DCM, diisopropylethylamine (0.84 mL, 6.51 mmol) and 2-cyanoethyl-diisopropyl-chlorophosphoramidite (0.83 mL, 3.50 mmol) were added. Under an atmosphere of nitrogen, the mixture was then stirred at room temperature for 2 hours. After the DCM was removed *in vacuo* a thick yellow oil was obtained (**13**) (1.63 g). ^{31}P NMR was run on the product, to prove the addition of the phosphoramidite group. No other analysis was run on it because of the air sensitive nature of the product. ^{31}P NMR (DMSO-d_6 capillary in DCM): δ 139.00 (s, 1P).

Addition of 2-cyanoethyl diisopropyl chlorophosphoramidite to DMT protected 2'-desoxy-2'-fluoro-5-iodo-uridine. 11 (1.068 g), and dimethylaminopyridine (0.17 g, 1.39 mmol) was dissolved in 50.0 mL of dry DCM, diisopropylethylamine (1.03 mL, 7.95 mmol) and 2-cyanoethyl-diisopropyl-chlorophosphoramidite (1.01 mL, 4.25 mmol) were added. Under an atmosphere of nitrogen, the mixture was then stirred at room temperature for 2 hours. After the DCM was removed *in vacuo* to obtained thick yellow oil (**14**) (1.98 g). ^{31}P NMR was run on the product, to prove the addition of the phosphoramidite group. No other analysis was run on it because of the air sensitive nature of the product. ^{31}P NMR (DMSO-d_6 capillary in DCM): δ 149.00 (s, 1P).

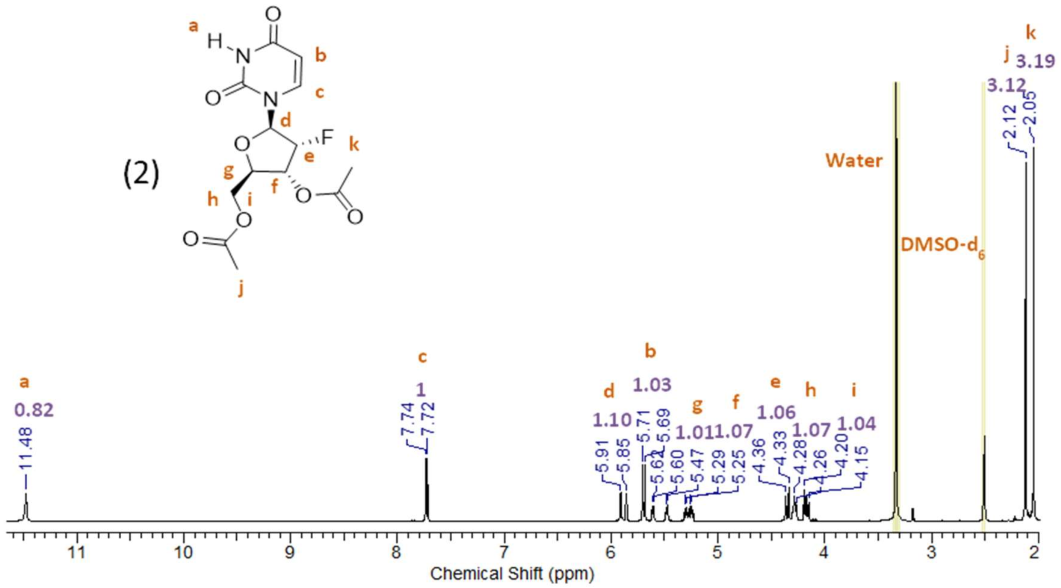

Figure 9. ^{1}H NMR spectrum of 2’-fluoro-2’-deoxyuridine (DMSO-d_6).

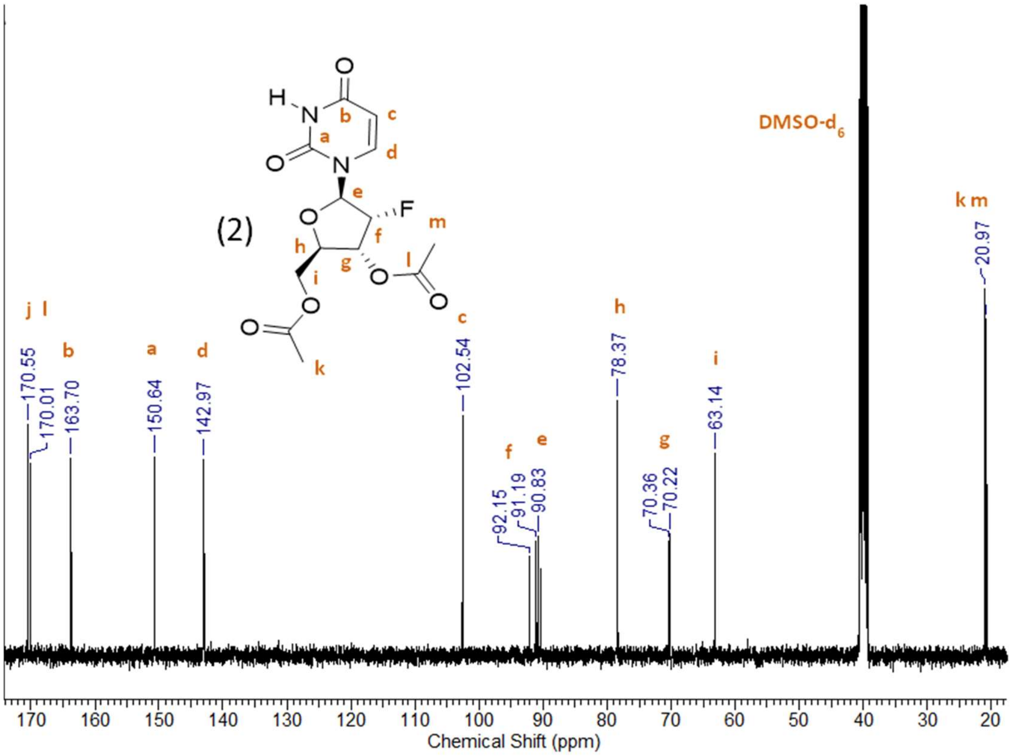

Figure 10. ^{13}C NMR spectrum of 2’-fluoro-2’-deoxyuridine (DMSO-d_6).

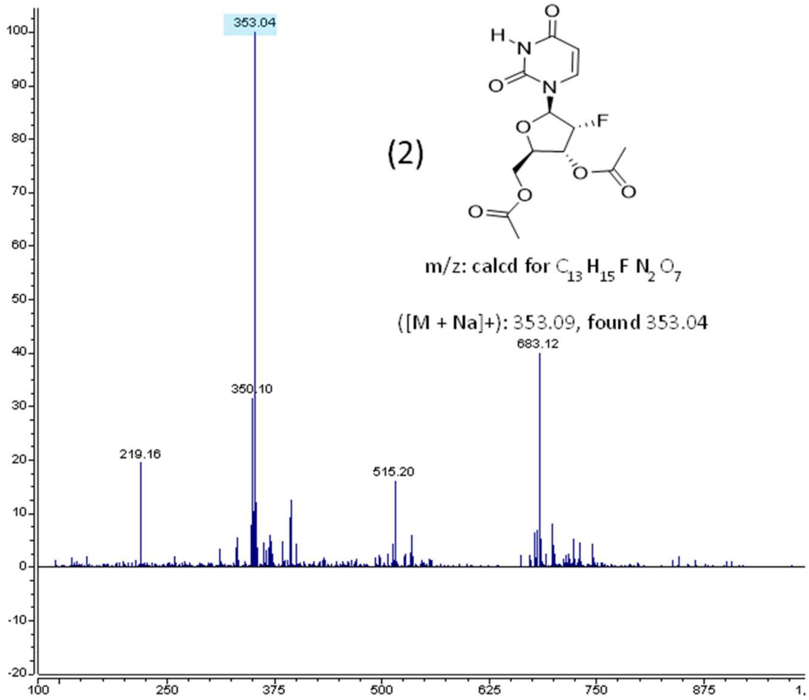

Figure 11. Mass spectrum of 2’-fluoro-2’-deoxyuridine.

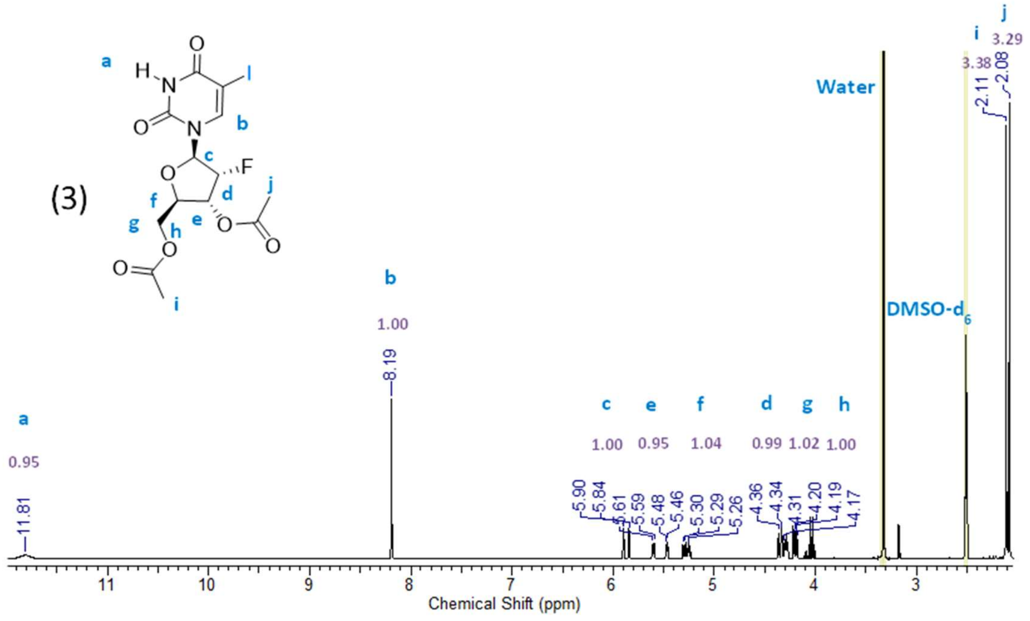

Figure 12. ^{1}H NMR spectrum of 3',5'-di-O-acetyl-2'-desoxy-2'-fluoro-5-iodo-uridine (DMSO-d_6).

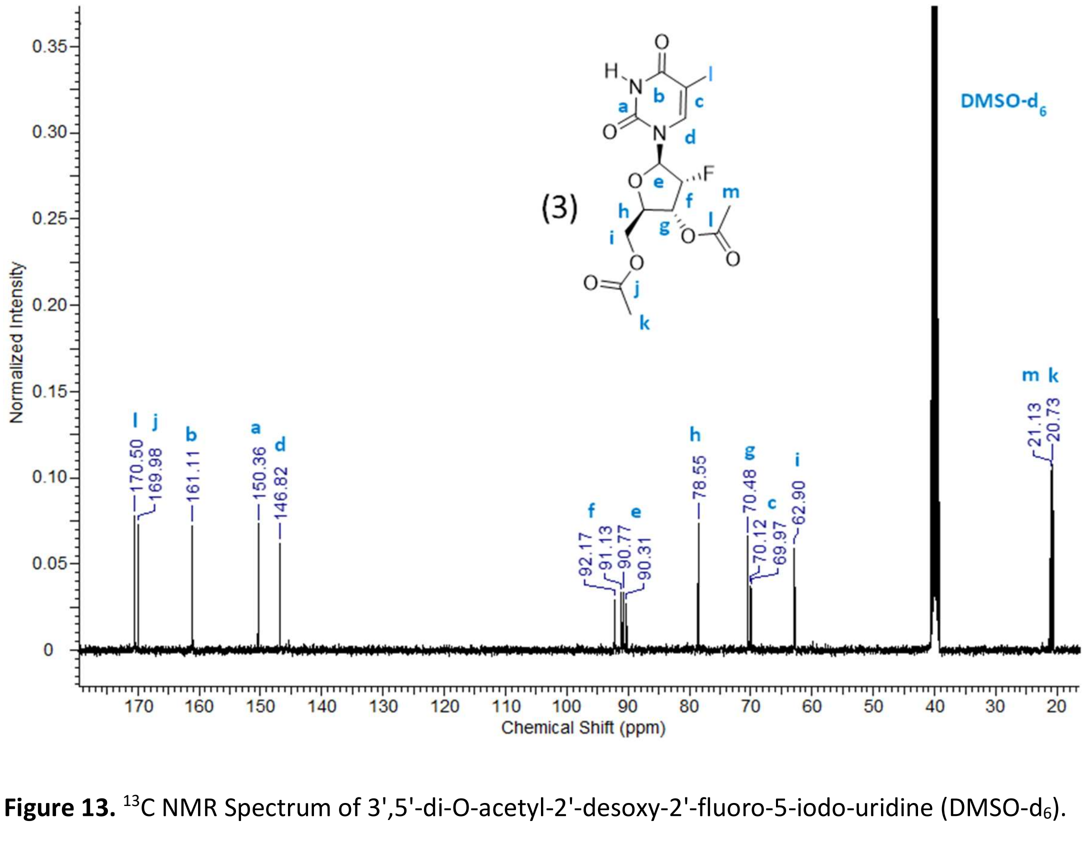

Figure 13. ^{13}C NMR Spectrum of 3',5'-di-O-acetyl-2'-desoxy-2'-fluoro-5-iodo-uridine (DMSO-d_6).

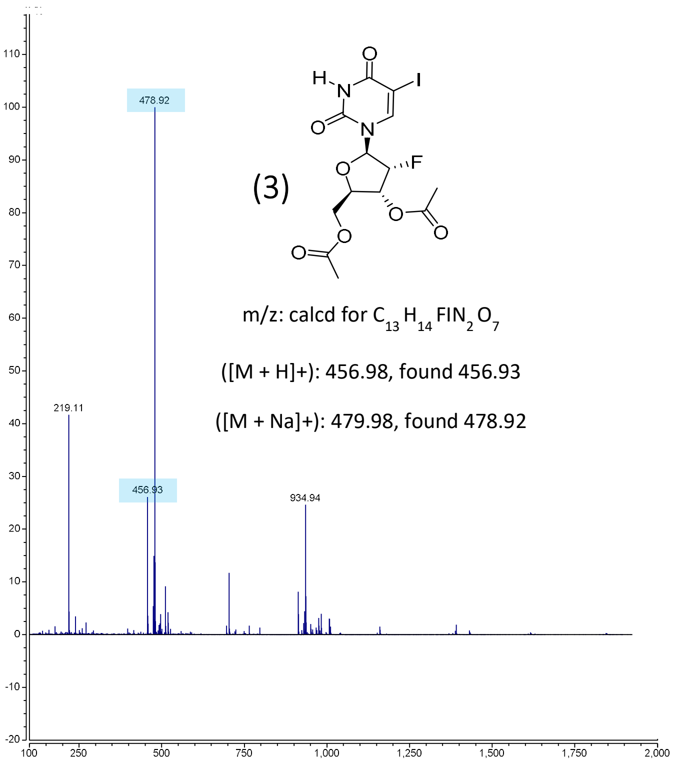

Figure 14. Mass spectrum of 3',5'-di-O-acetyl-2'-desoxy-2'-fluoro-5-iodo-uridine.

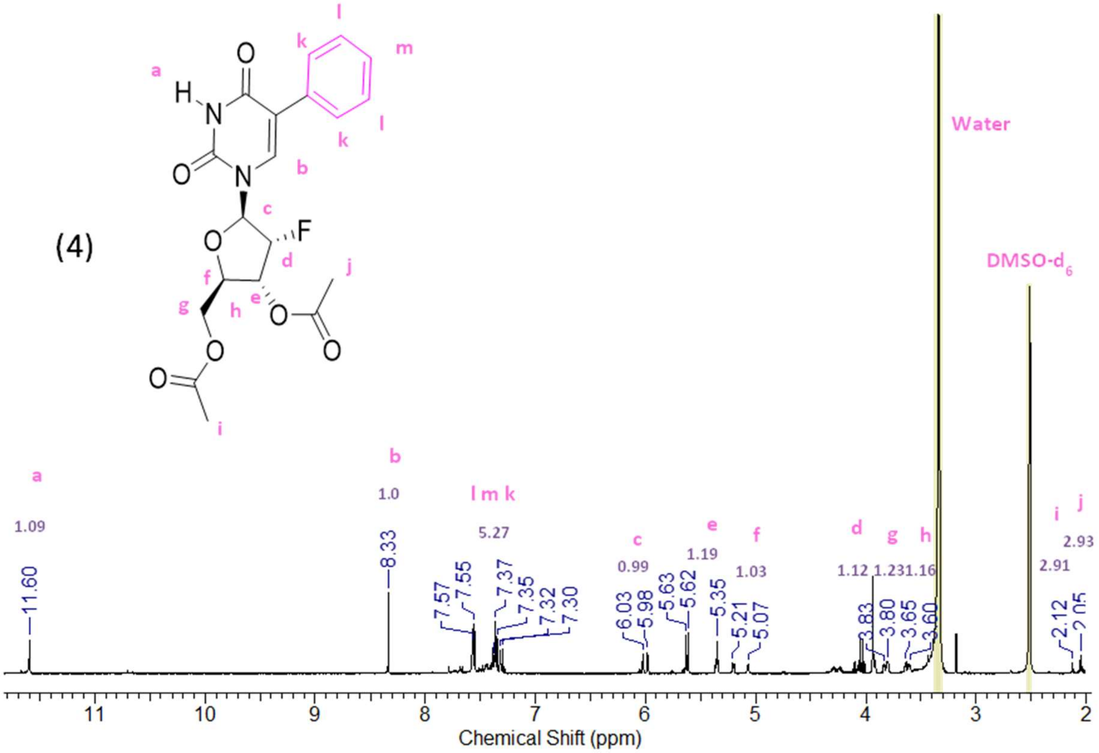

Figure 15. ^{1}H NMR spectrum of 3',5'-di-O-acetyl-2'-desoxy-2'-fluoro-5-phenyl-uridine (DMSO-d_6).

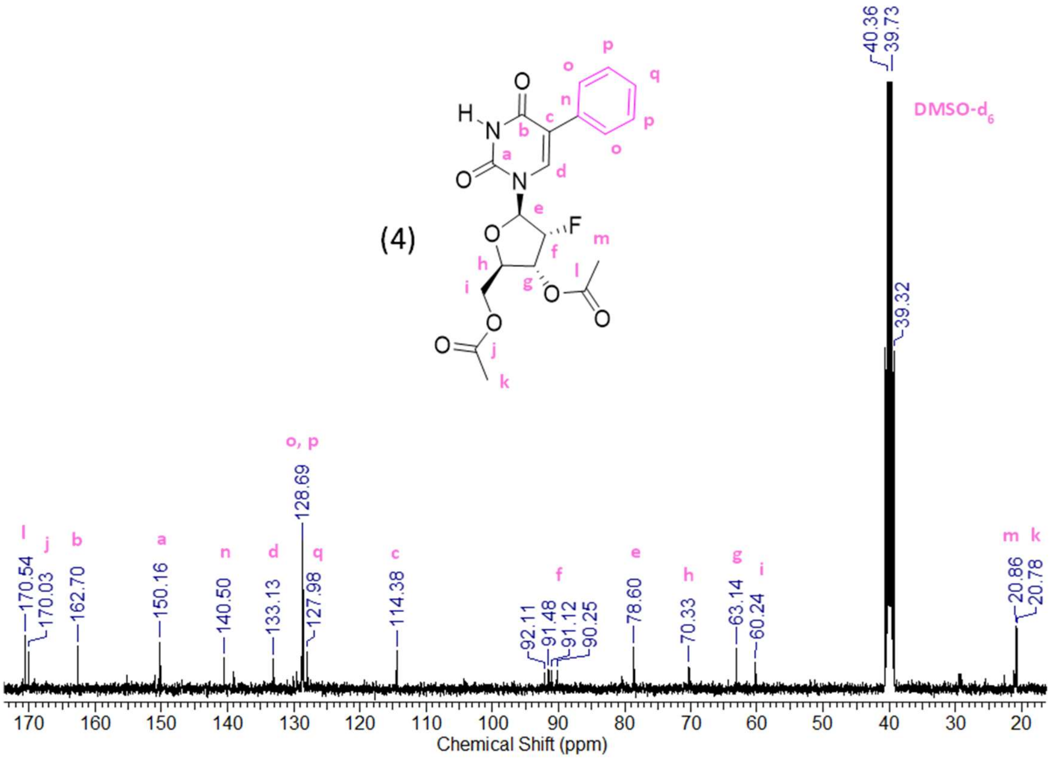

Figure 16. ^{13}C NMR Spectrum of 3',5'-di-O-acetyl-2'-desoxy-2'-fluoro-5-phenyl-uridine (DMSO-d_6).

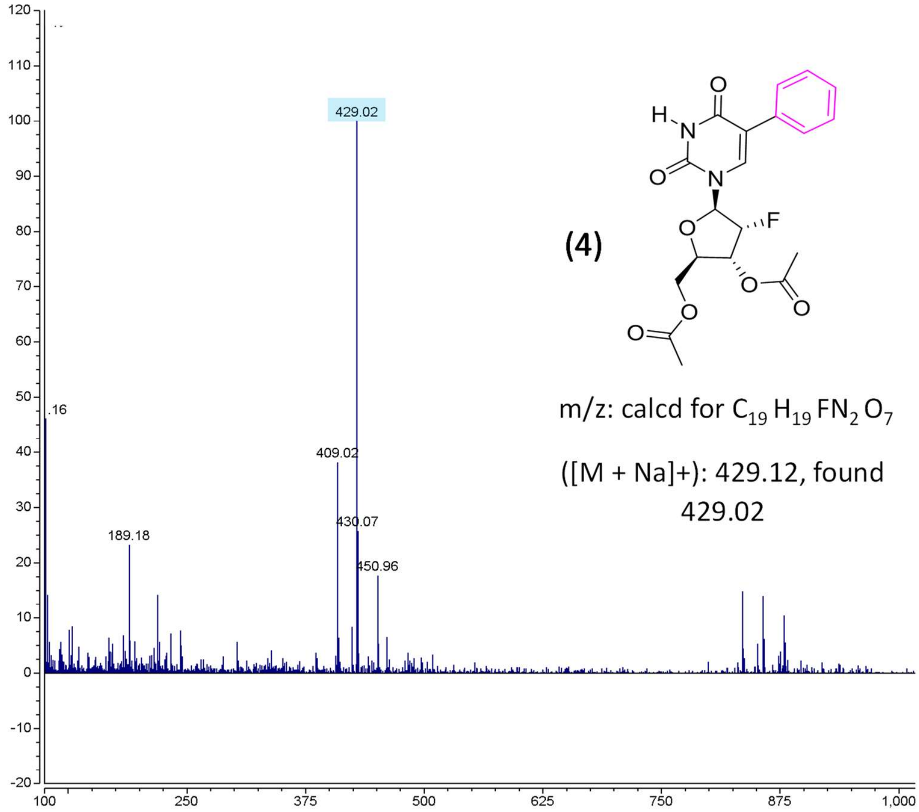

Figure 17. Mass spectrum of 3',5'-di-O-acetyl-2'-desoxy-2'-fluoro-5-phenyl-uridine.

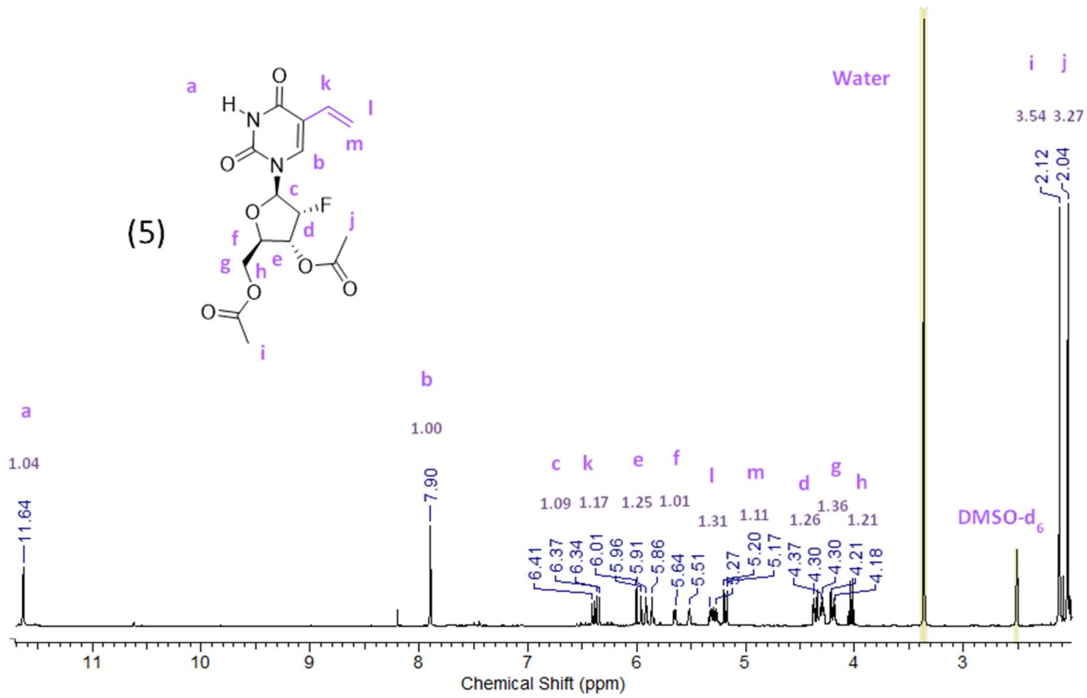

Figure 18. ^{1}H NMR spectrum of 3',5'-di-O-acetyl-2'-desoxy-2'-fluoro-5-vinyl-uridine (DMSO-d_6).

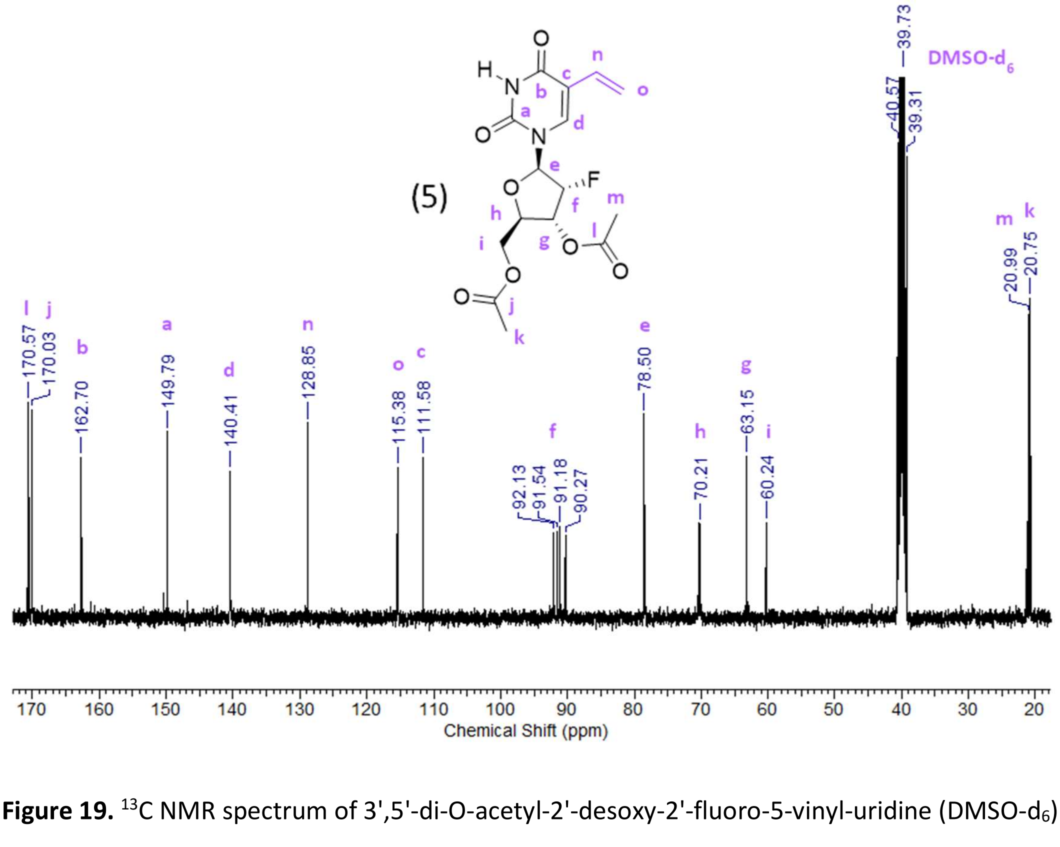

Figure 19. ^{13}C NMR spectrum of 3',5'-di-O-acetyl-2'-desoxy-2'-fluoro-5-vinyl-uridine (DMSO-d_6).

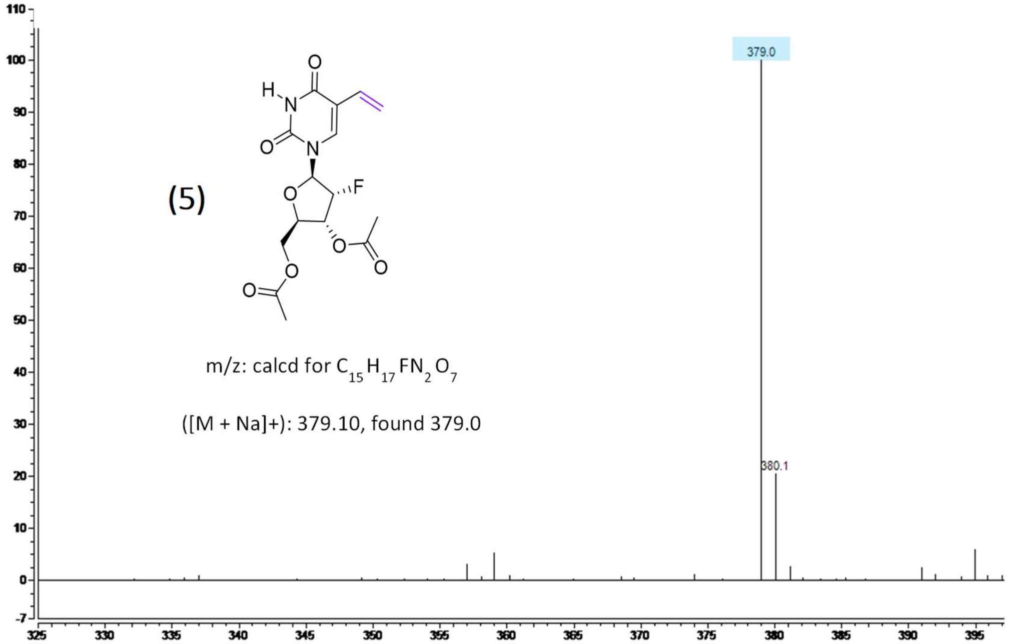

Figure 20. Mass spectrum of 3',5'-di-O-acetyl-2'-desoxy-2'-fluoro-5-vinyl-uridine.

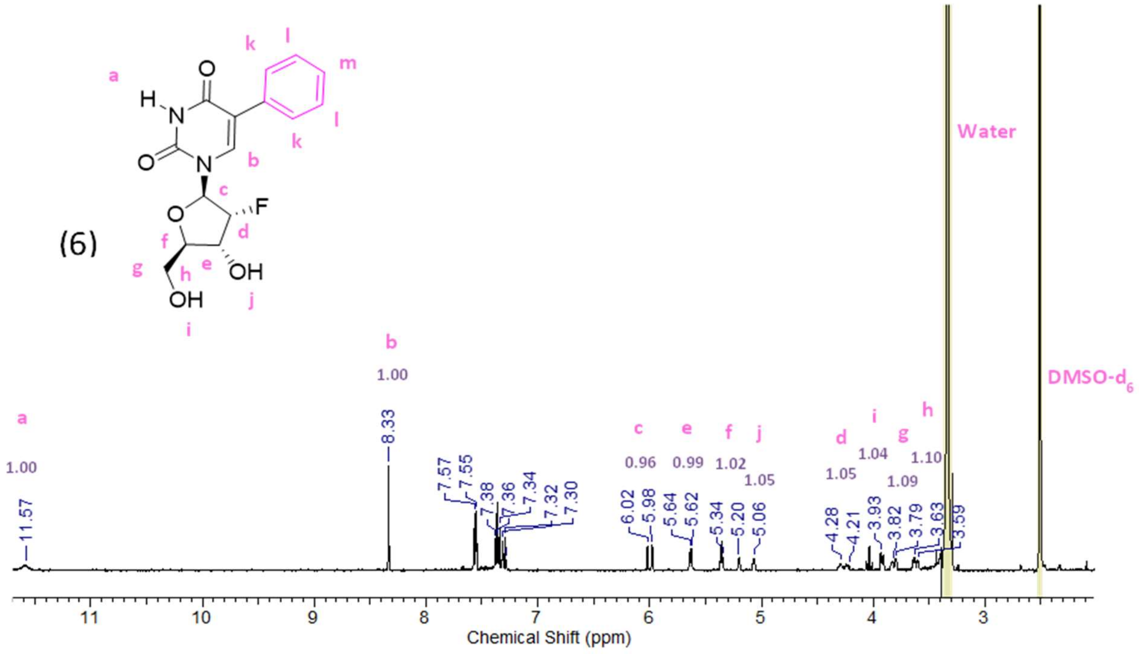

Figure 21. ^{1}H NMR spectrum of 2'-desoxy-2'-fluoro-5-phenyl-uridine (DMSO-d_6).

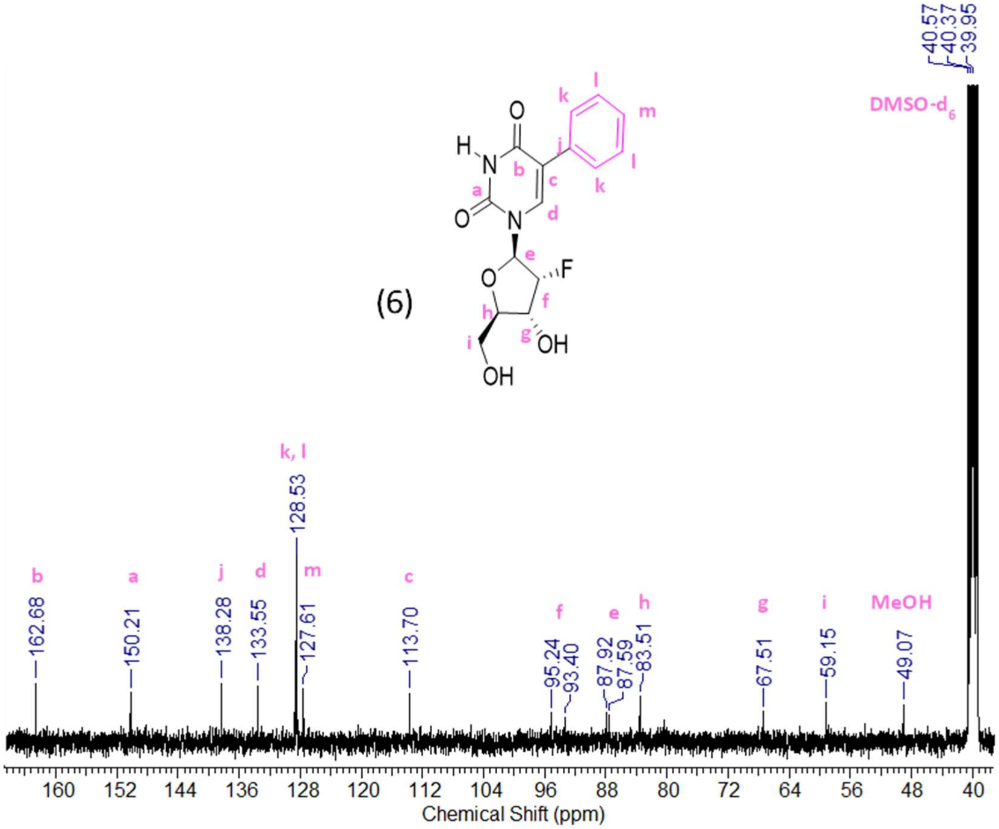

Figure 22. ^{13}C NMR Spectrum of 2'-desoxy-2'-fluoro-5-phenyl-uridine (DMSO-d_6).

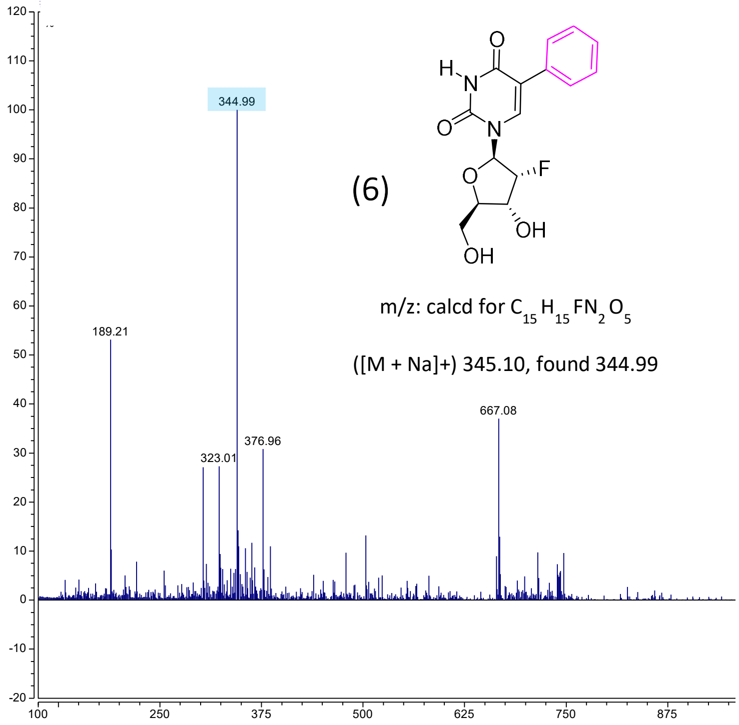

Figure 23. Mass spectrum of 2'-desoxy-2'-fluoro-5-phenyl-uridine.

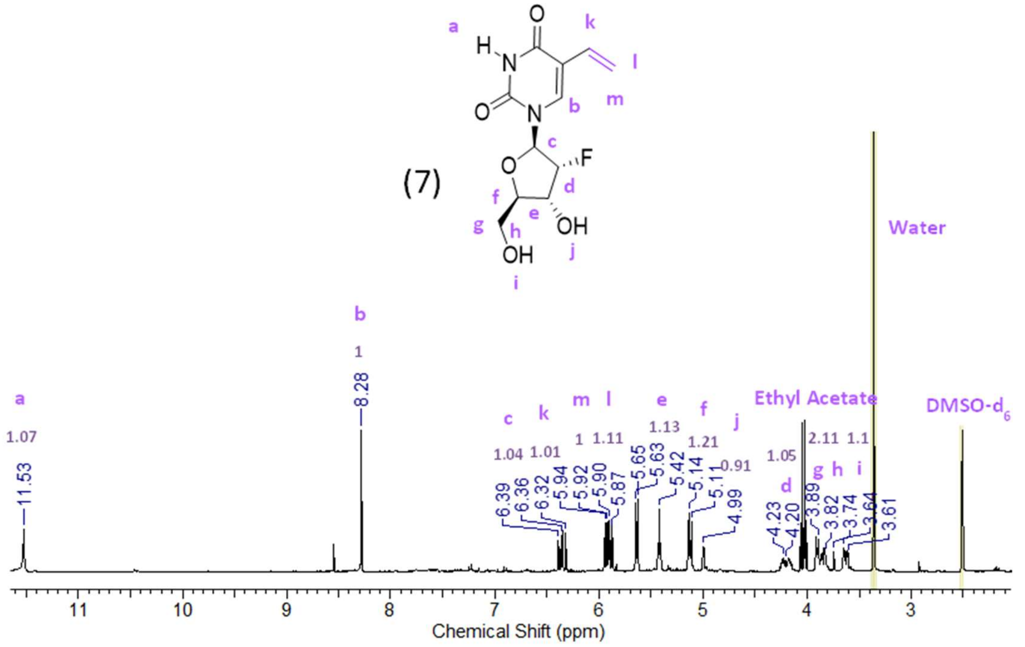

Figure 24. ^{1}H NMR spectrum of 2'-desoxy-2'-fluoro-5-vinyl-uridine (DMSO-d_6).

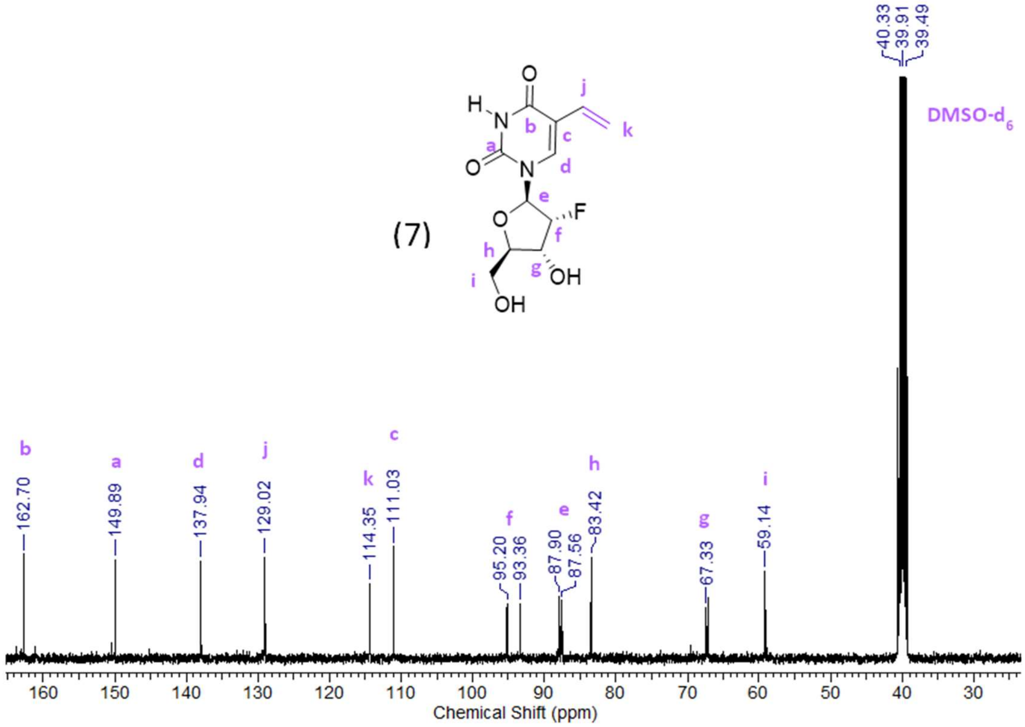

Figure 25. ^{13}C NMR spectrum of 2'-desoxy-2'-fluoro-5-vinyl-uridine (DMSO-d_6).

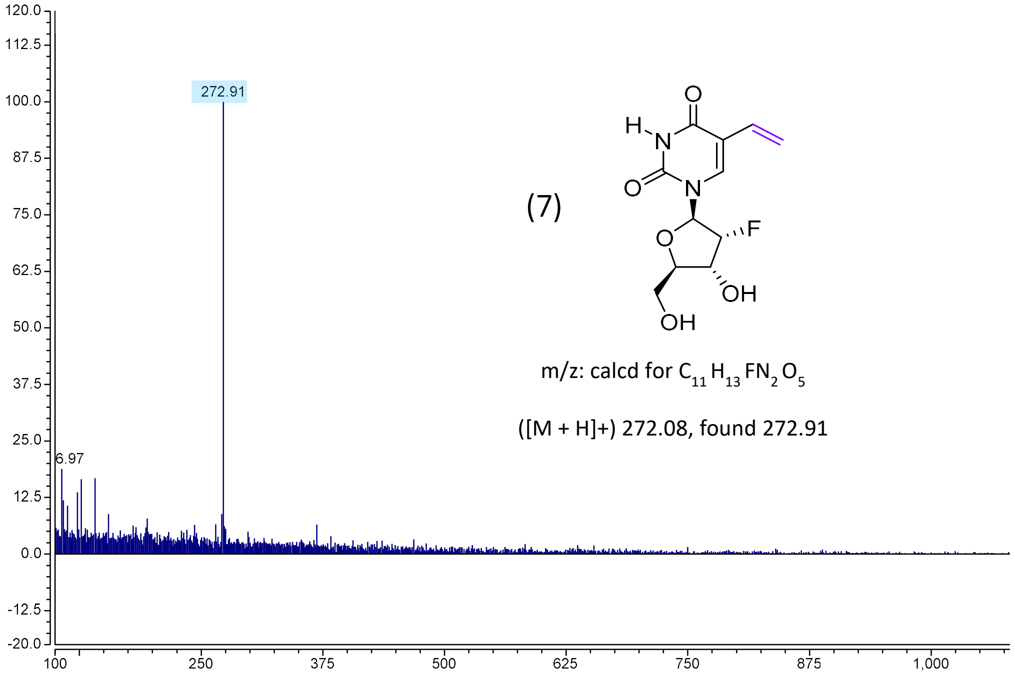

Figure 26. Mass spectrum of 2'-desoxy-2'-fluoro-5-vinyl-uridine.

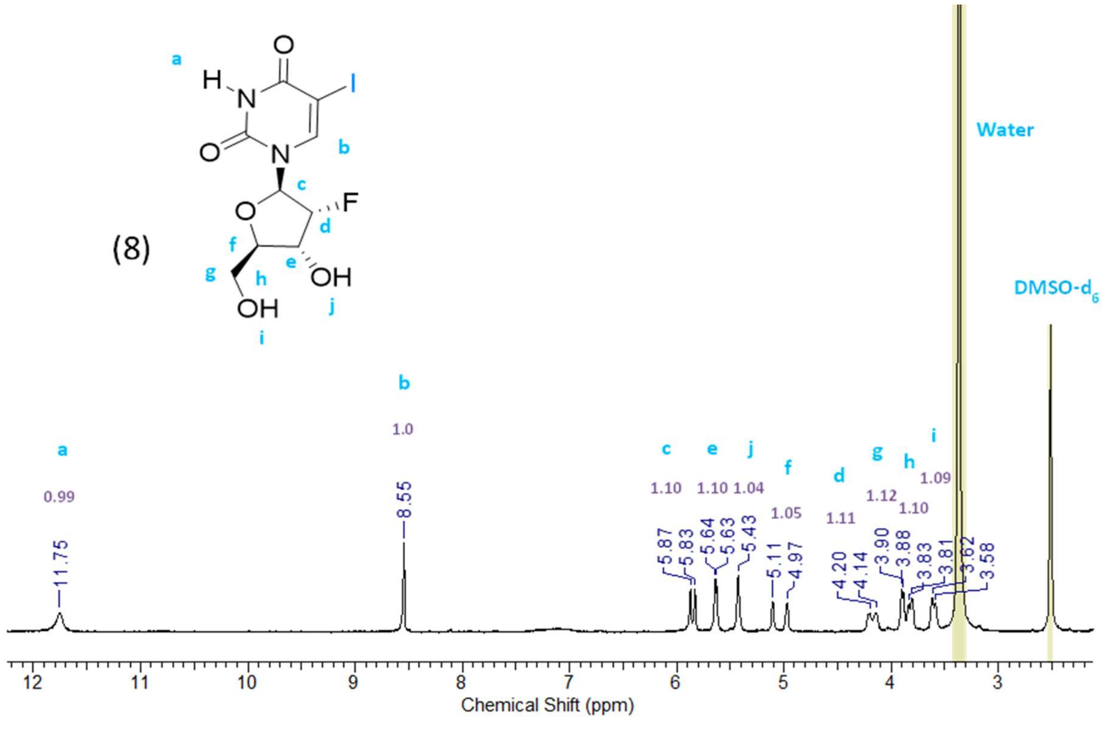

Figure 27. ^{1}H NMR spectrum of 2'-desoxy-2'-fluoro-5-iodo-uridine (DMSO-d_6).

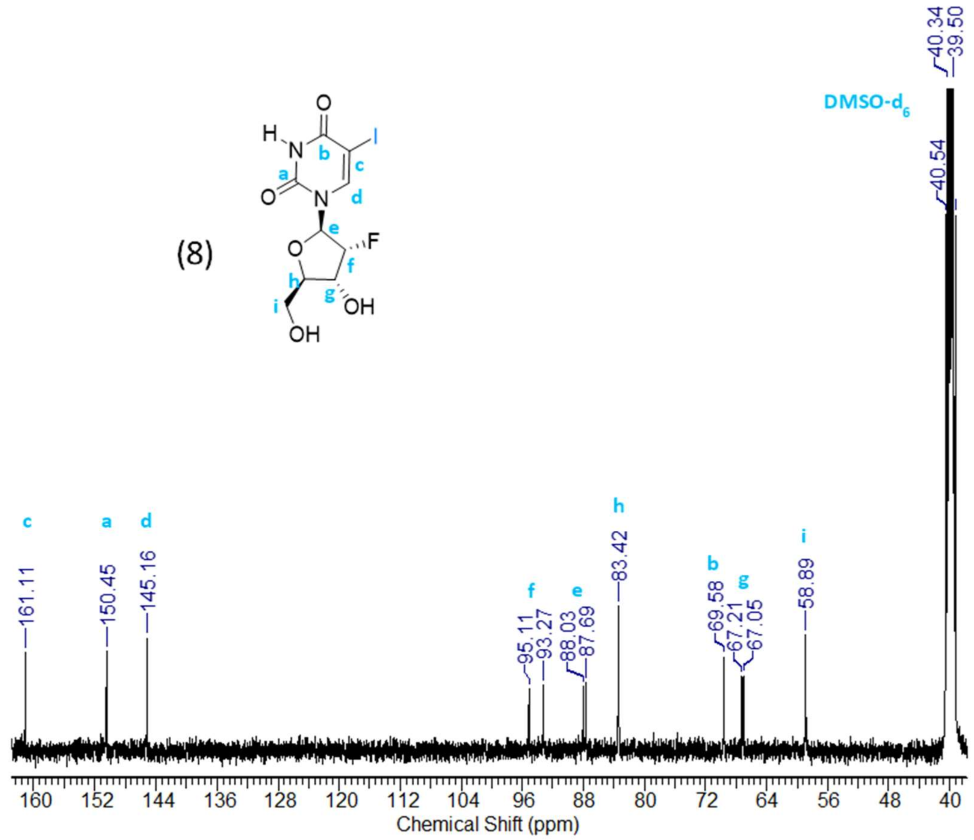

Figure 28. ^{13}C NMR spectrum of 2'-desoxy-2'-fluoro-5-iodo-uridine (DMSO-d_6).

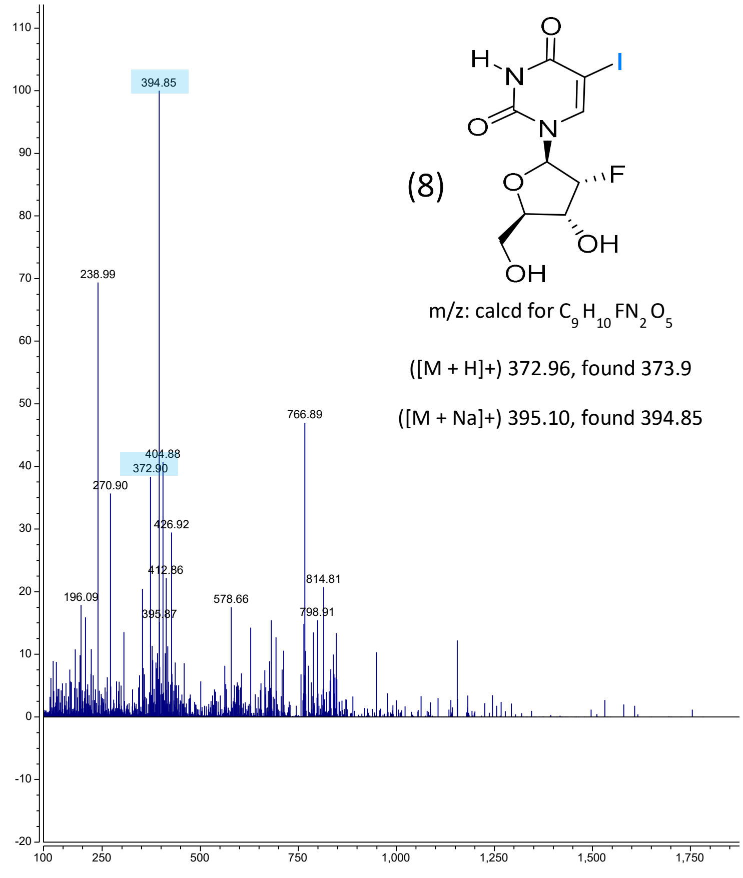

Figure 29. Mass spectrum of 2'-desoxy-2'-fluoro-5-iodo-uridine.

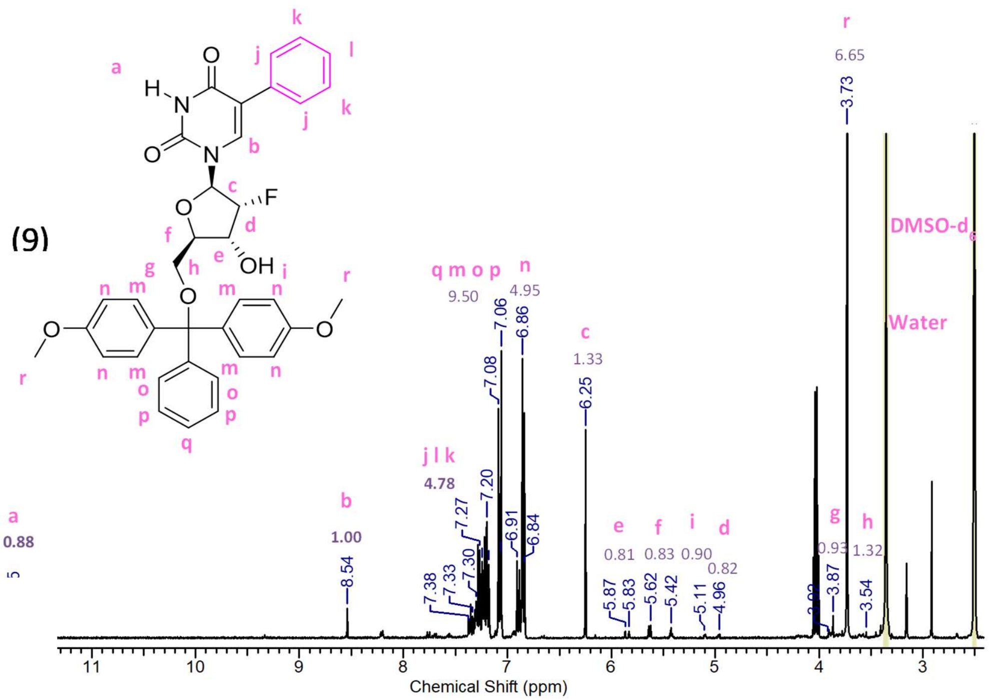

30. ^{1}H NMR spectrum of 4’4-DMT tagged 2'-desoxy-2'-fluoro-5-phenyl-uridine (DMSO-d_6).

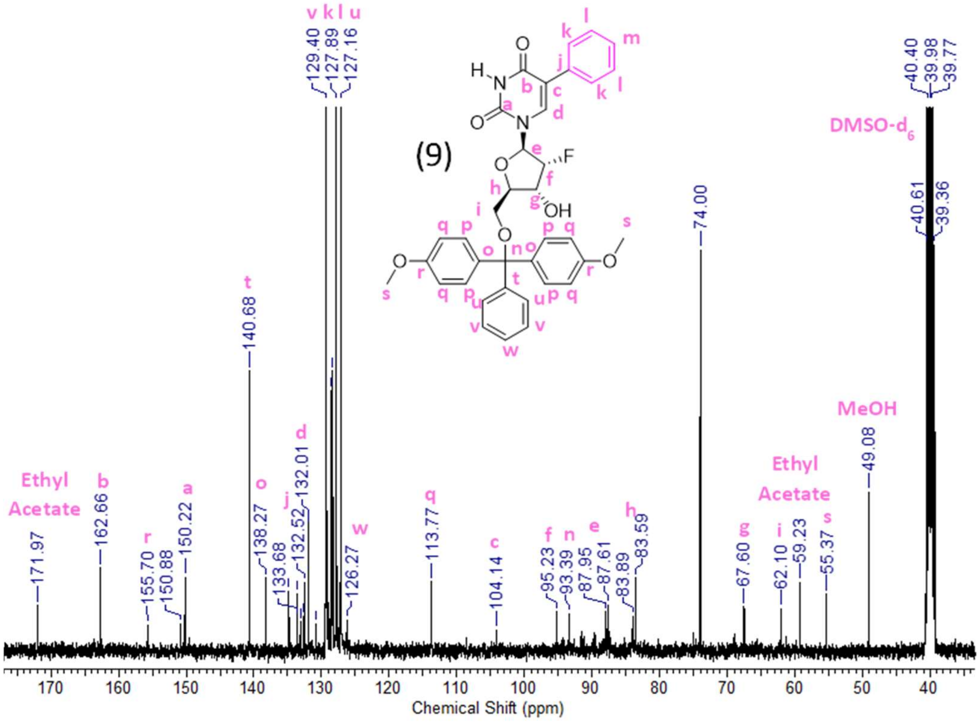

Figure 31. ^{13}C NMR spectrum of 4’4-DMT tagged 2'-desoxy-2'-fluoro-5-phenyl-uridine (DMSO-d_6).

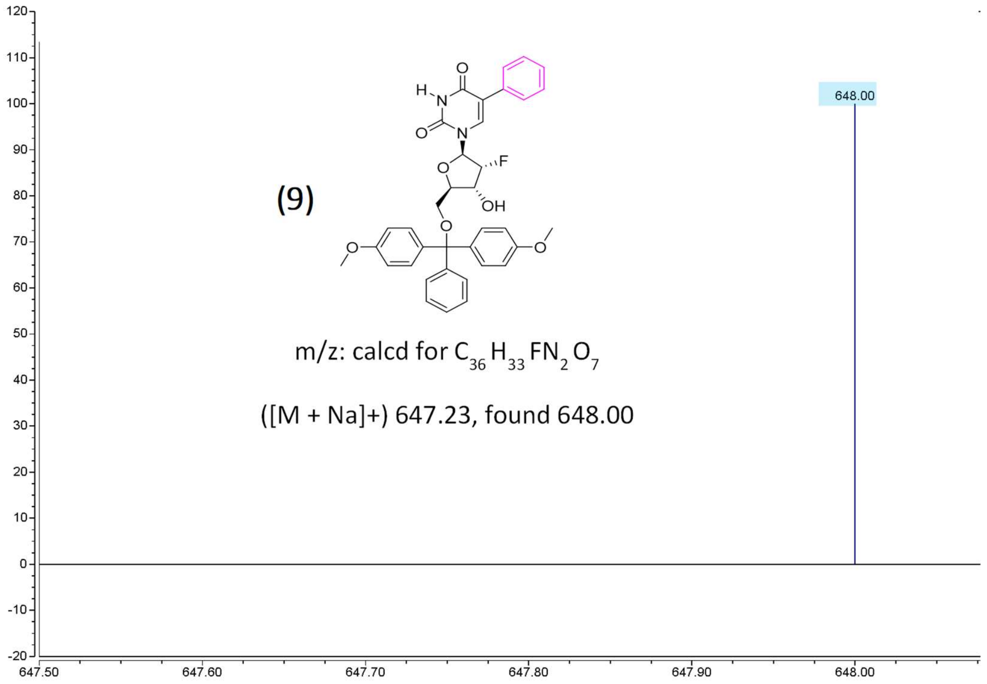

Figure 32. Mass spectrum of 4’4-DMT tagged 2'-desoxy-2'-fluoro-5-phenyl-uridine.

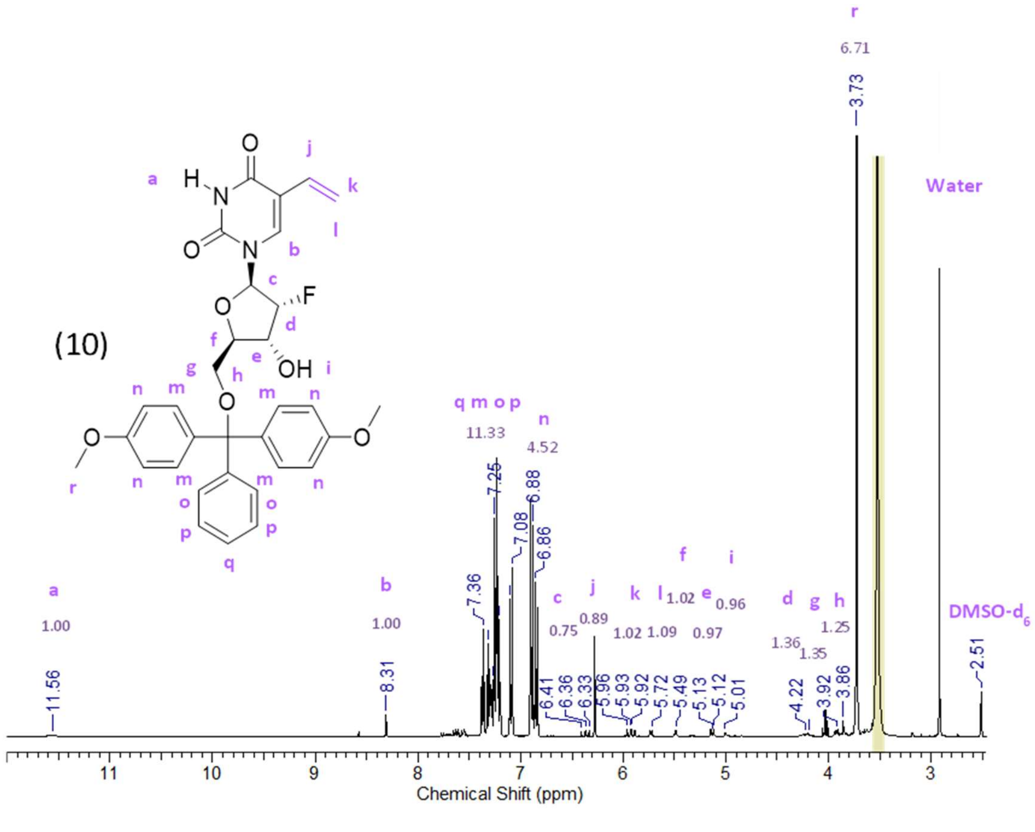

Figure 33. ^{1}H NMR spectrum of 4’4-DMT tagged 2'-desoxy-2'-fluoro-5-vinyl-uridine (DMSO-d_6).

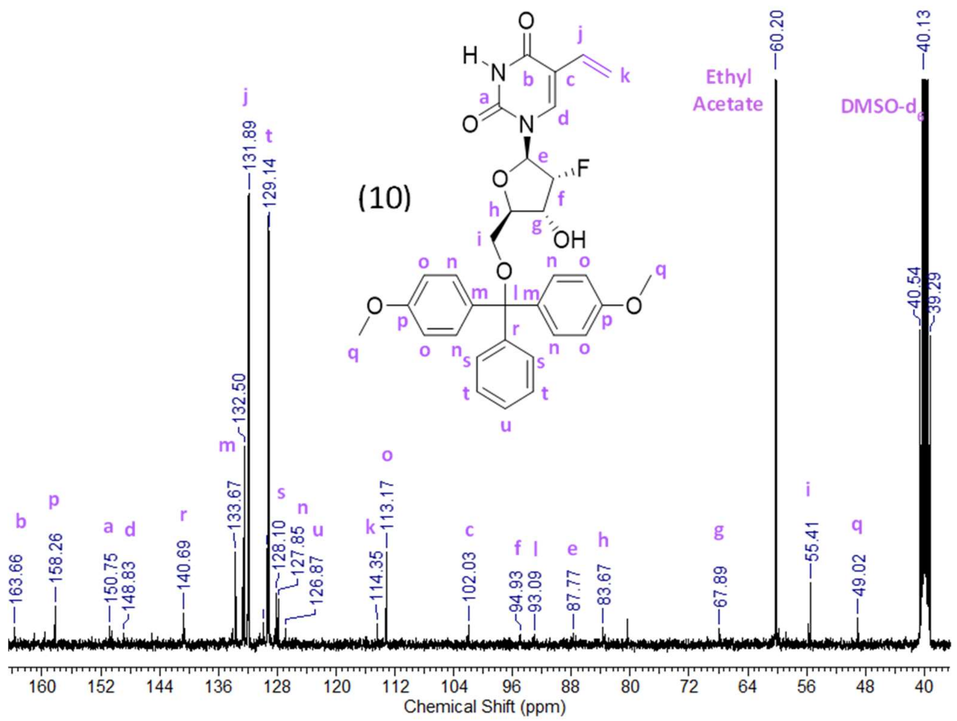

Figure 34. ^{13}C NMR spectrum of 4’4-DMT tagged 2'-desoxy-2'-fluoro-5-vinyl-uridine (DMSO-d_6).

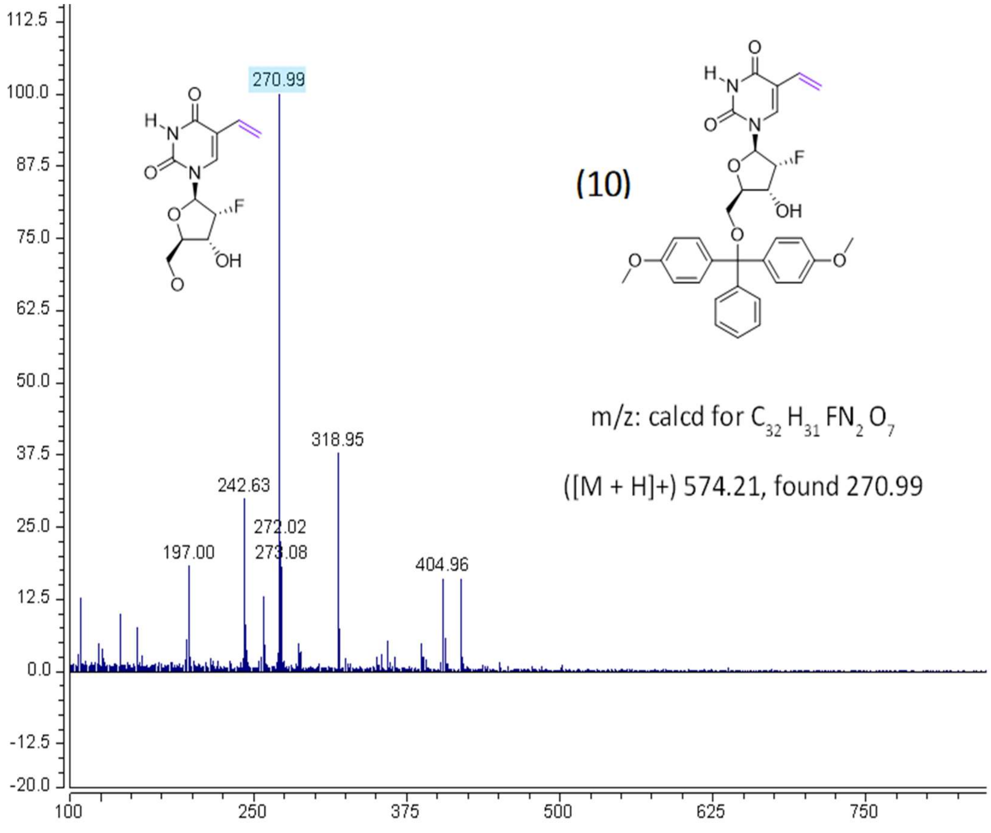

Figure 35. Mass spectrum of 4’4-DMT tagged 2'-desoxy-2'-fluoro-5-vinyl-uridine.

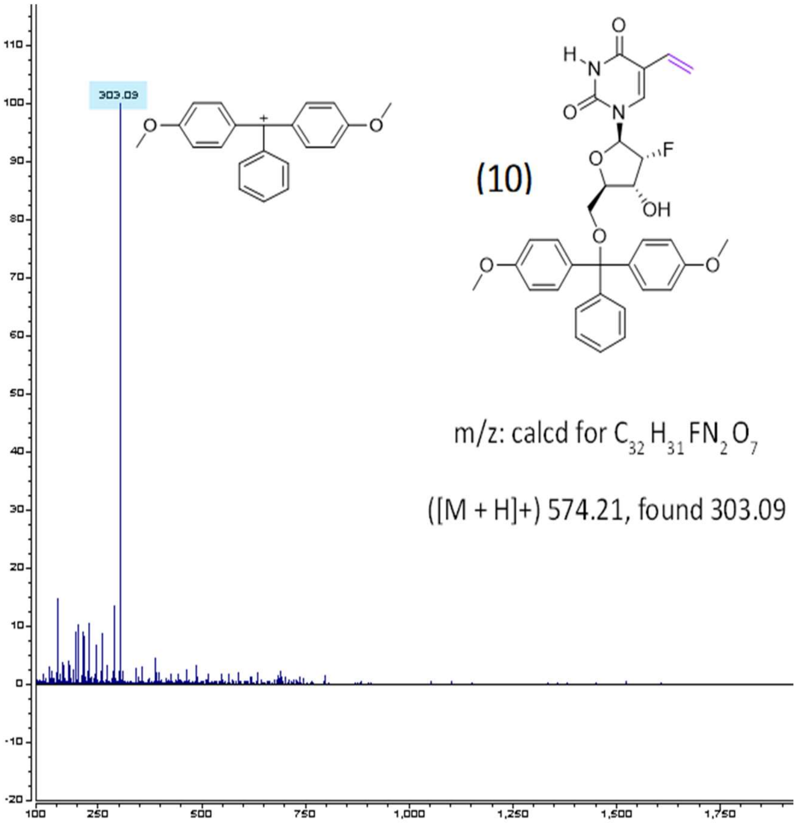

Figure 36. Mass spectrum of 4’4-DMT tagged 2'-desoxy-2'-fluoro-5-vinyl-uridine.

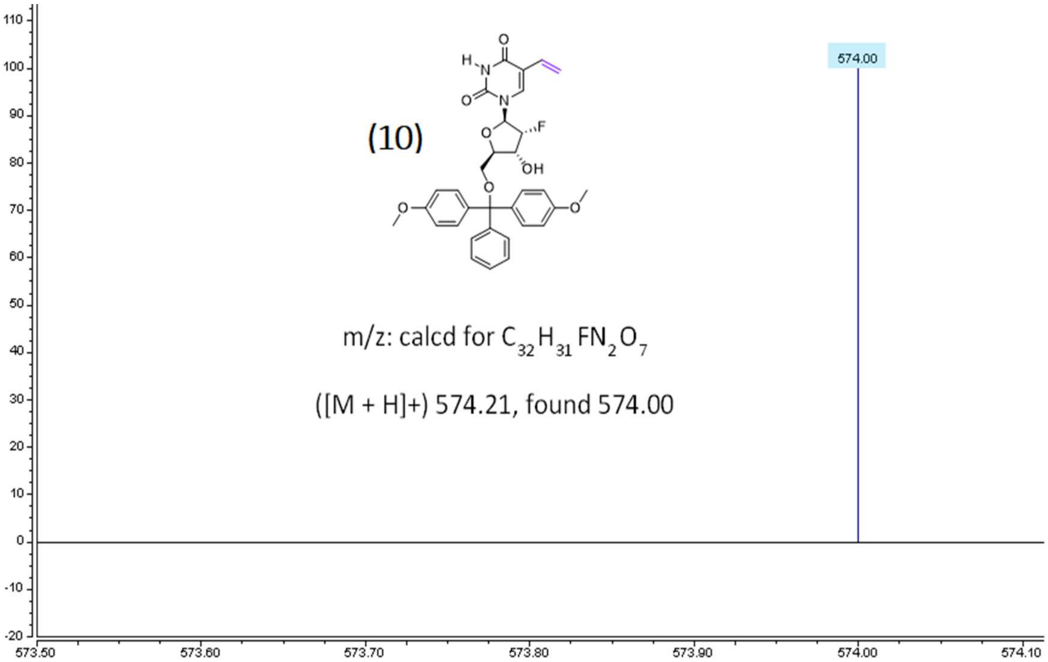

Figure 37. Mass spectrum of 4’4-DMT tagged 2'-desoxy-2'-fluoro-5-vinyl-uridine.

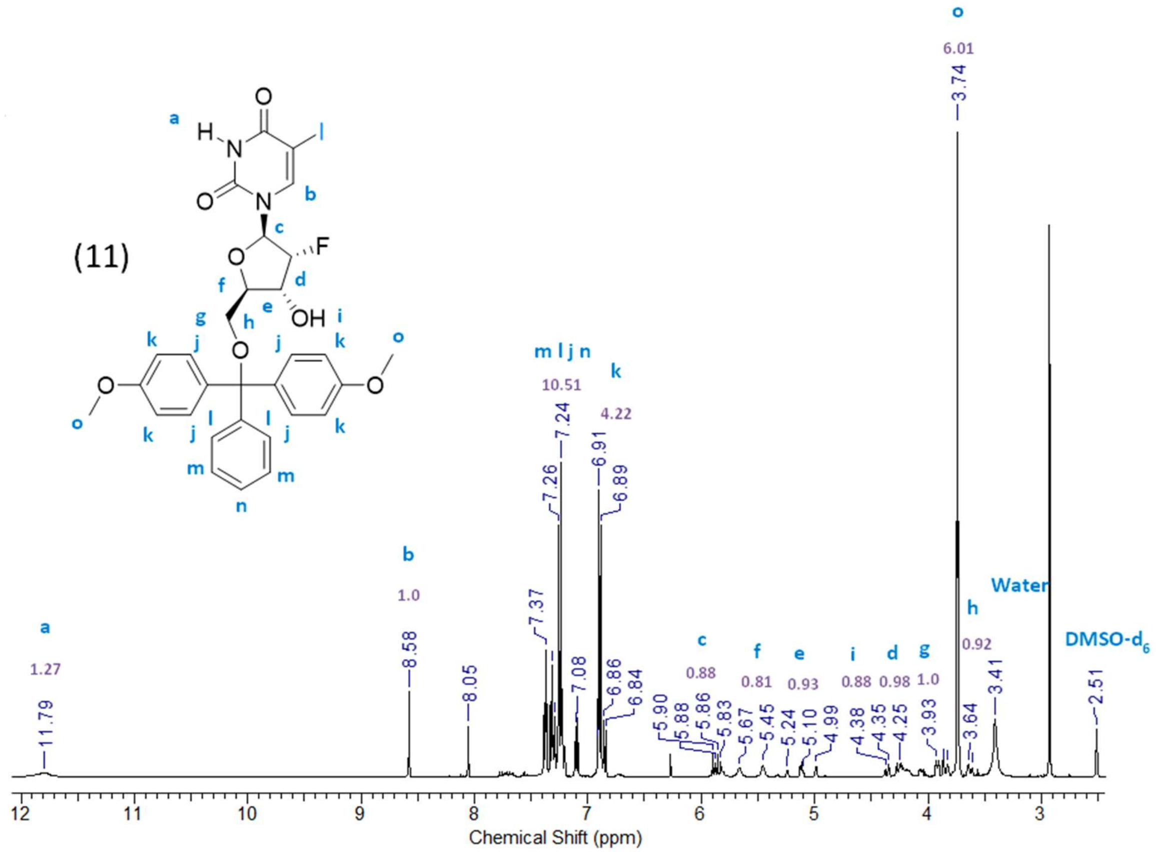

Figure 38. ^{1}H NMR spectrum of 4’4-DMT tagged 2'-desoxy-2'-fluoro-5-iodo-uridine (DMSO-d$_6$).

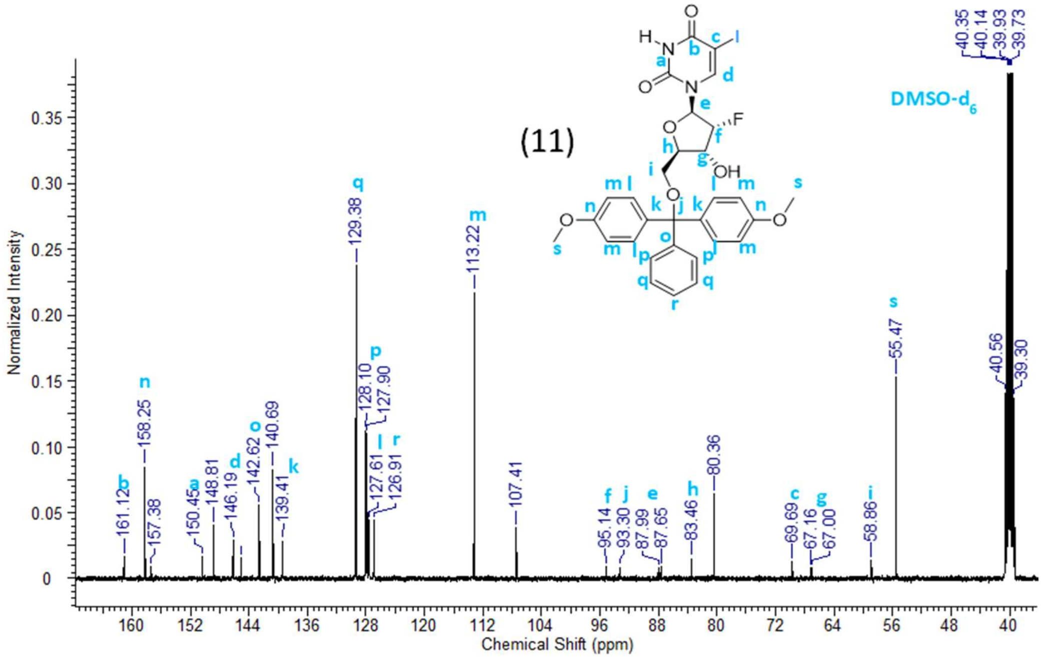

Figure 39. ^{13}C NMR spectrum of 4’4-DMT tagged 2'-desoxy-2'-fluoro-5-iodo-uridine (DMSO-d$_6$).

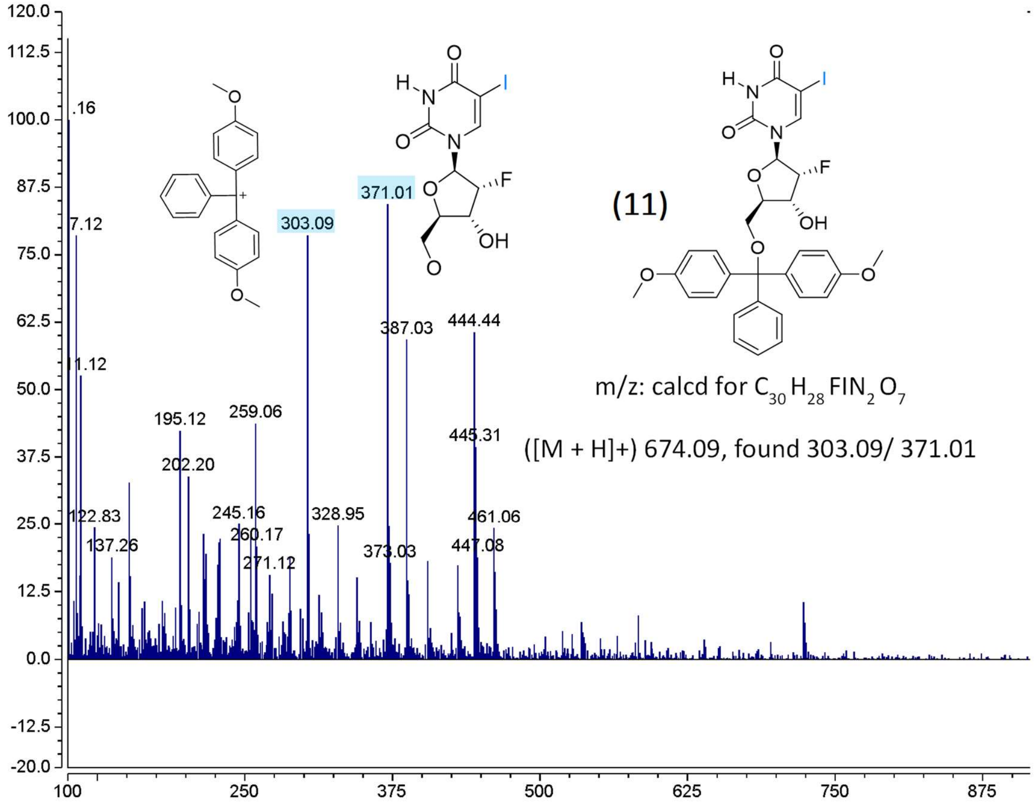

Figure 40. Mass spectrum of 4'4-DMT tagged 2'-desoxy-2'-fluoro-5-iodo-uridine.

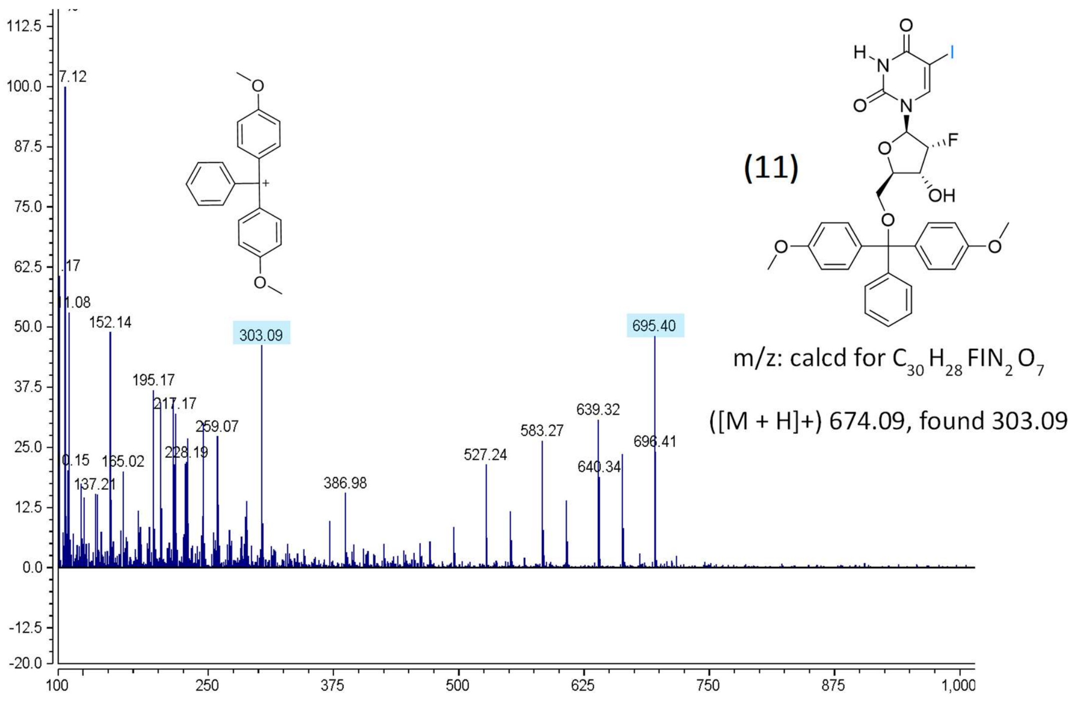

Figure 41. Mass spectrum of 4'4-DMT tagged 2'-desoxy-2'-fluoro-5-iodo-uridine.

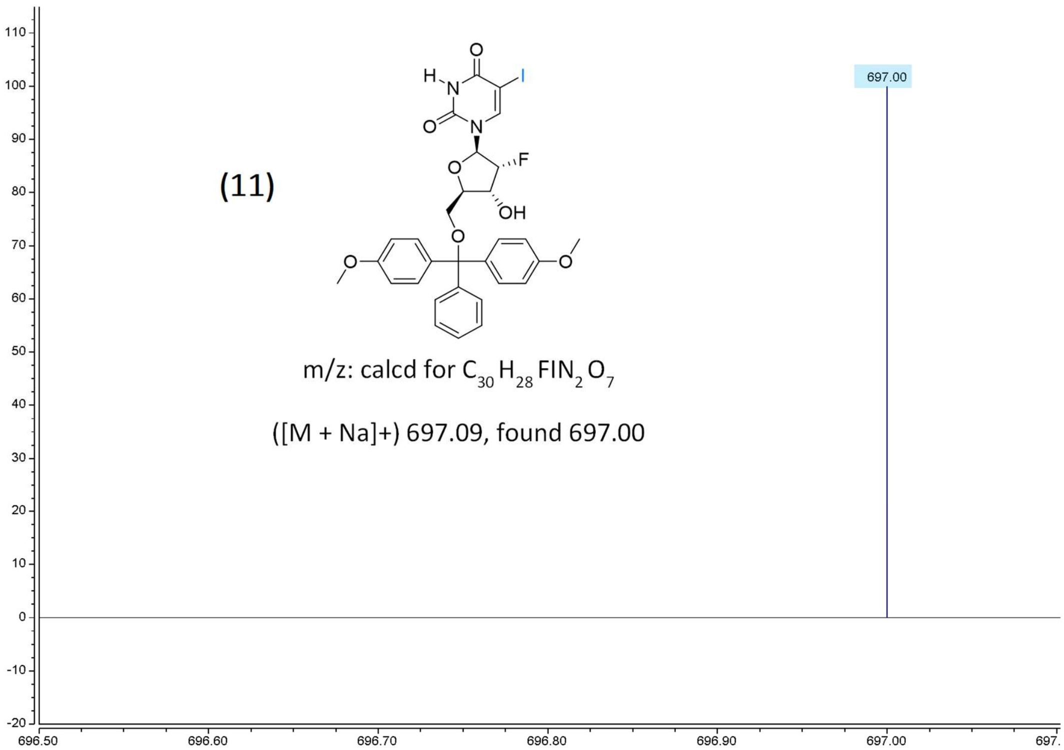

Figure 42. Mass spectrum of 4'4-DMT tagged 2'-desoxy-2'-fluoro-5-iodo-uridine.

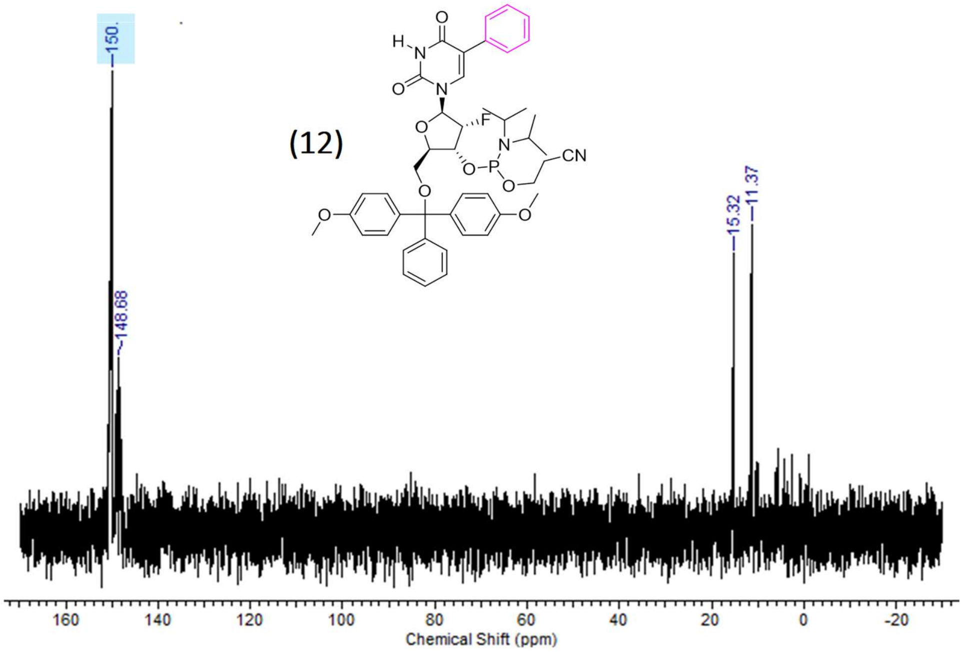

Figure 43. ^{31}P NMR spectrum of 4'4-DMT tagged 2'-desoxy-2'-fluoro-5-phenyl-uridine phosphoramidite (CH_2Cl_2).

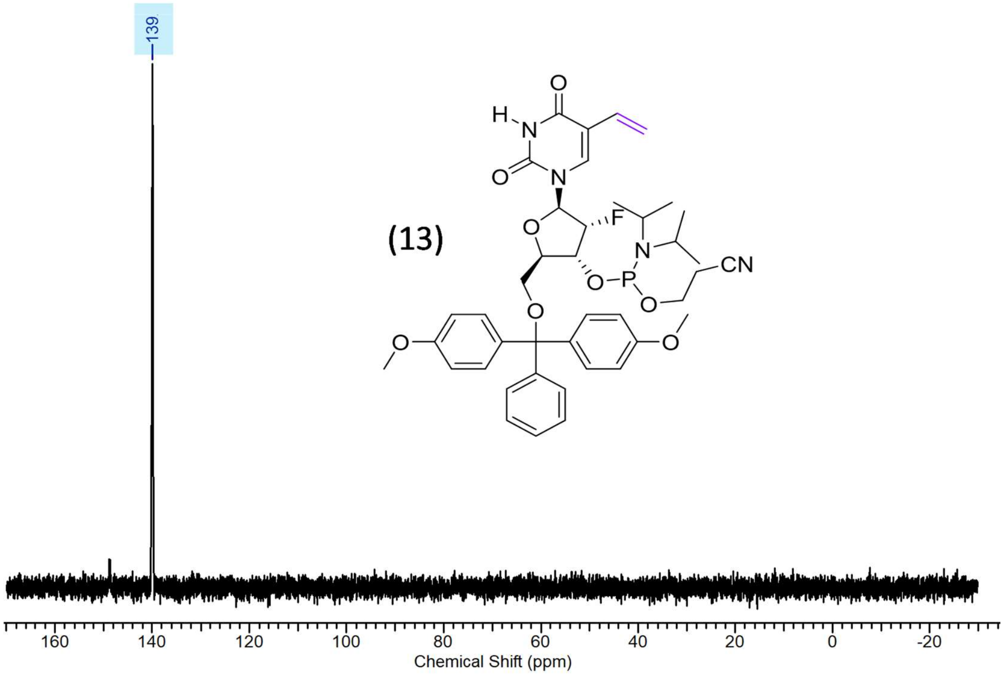

Figure 44. ^{31}P NMR spectrum of 4'4-DMT tagged 2'-desoxy-2'-fluoro-5-vinyl-uridine phosphoramidite (CH_2Cl_2).

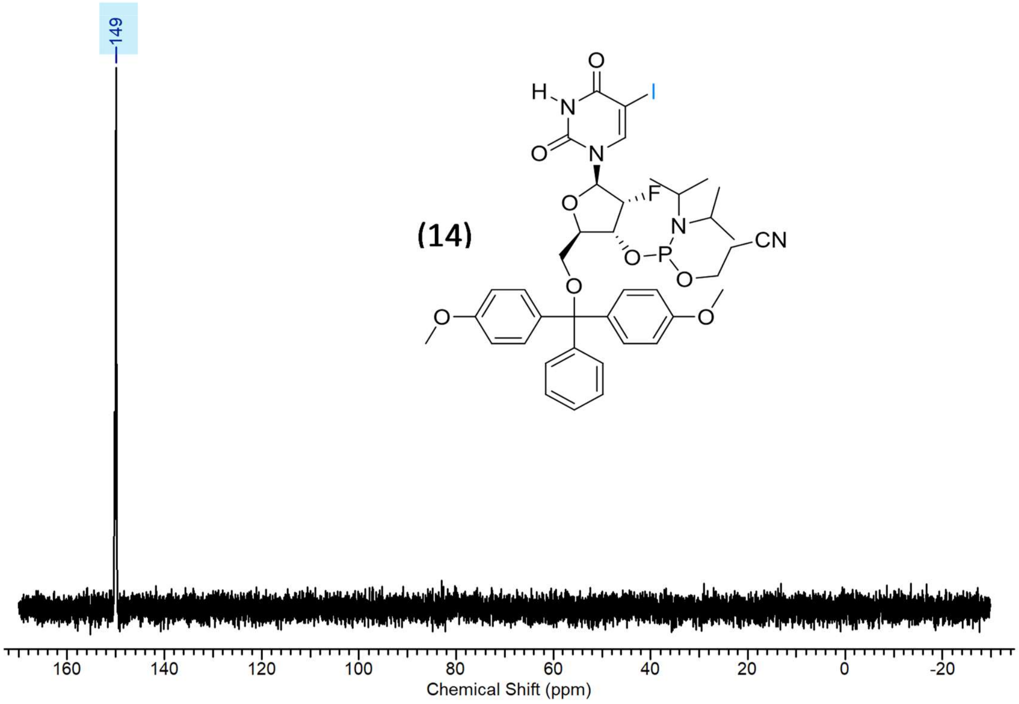

Figure 45. ^{31}P NMR spectrum of 4'4-DMT tagged 2'-desoxy-2'-fluoro-5-iodo-uridine phosphoramidite (CH_2Cl_2).

S4. Aptamer Library Synthesis

General Expedite™ 8909 DNA Synthesiser set up for the synthesis of aptamers and aptamer library. The phosphoramidite samples were dissolved in 20 mL (20 mL for 1 g of sample) acetonitrile (DCM for Tentagel beads), put in bottles and screwed into the synthesiser lines. The other reagents put on to the machine: oxidizer (0.02M iodine, 20% pyridine), Cap A Mix (THF/Pyridine/acetic anhydride 8:1:1), Cap B Mix (10% methylimidazole in THF), deblock (3% trimethylamine in DCM) and ETT activator solution (0.25 M, 5-ethylthio-1H-tetrazole in acetonitrile). A leak test is run to check nitrogen is not leaking from the lines. If passed, the lines are then flushed with the new reagents added to them. The beads are added to the column, which is then fitted onto the synthesiser, which is then flushed with acetonitrile several times. The sequence and protocol are then selected using Validate XP connected to the Expedite™ 8909 DNA Synthesiser. The aptamer sequences are then run and monitored using the trityl monitor.

Synthesis of MinE07 Aptamers. All library components were synthesised on an Expedite™ 8909 Nucleic Acid Synthesiser system provided by Biolytic. Phosphoramidites were dissolved in dry acetonitrile to the concentrations as suggested by the supplier, solvents were used as provided. Oligomers were synthesised on a 1 µM scale. Universal UnyLinker support (0.021g) was added to a synthesiser column. See **General Expedite™ 8909 DNA Synthesiser set up for the synthesis of aptamers and aptamer library** for the setup of the synthesiser. The sequences uploaded on to the synthesiser:

MinE07:5'-rGrGrAfCrGrGrAfUfUfUrArAfUfCrGfCfCrGfUrArGrArArArArGfCrAfUrGfUfCrArArArGfCfCrGrGrArAfCfCrGfUfCfC-3'.
MinE07-Biotin:5'-Biotin-rGrGrAfCrGrGrAfUfUfUrArAfUfCrGfCfCrGfUrArGrArArArArGfCrAfUrGfUfCrArArArGfCfCrGrGrArAfCfCrGfUfCfC-3'.
MinE07-U-Ph-Biotin:5'-Biotin-rGrGrAfCrGrGrAfU(U-Ph)fU(U-Ph)fU(U-Ph)rArAfU(U-Ph)fCrGfCfCrGfU(U-Ph)rArGrArArArArGfCrAfU(U-Ph)rGfU(U-Ph)fCrArArArGfCfCrGrGrArAfCfCrGfU(U-Ph)fCfC-3'.
MinE07-U-Vi-Biotin:5'-Biotin-rGrGrAfCrGrGrAfU(U-Vi)fU(U-Vi)fU(U-Vi)rArAfU(U-Vi)fCrGfCfCrGfU(U-Vi)rArGrArArArArGfCrAfU(U-Vi)rGfU(U-Vi)fCrArArArGfCfCrGrGrArAfCfCrGfU(U-Vi)fCfC-3'.
MinE07-U-I-Biotin:5'-Biotin-rGrGrAfCrGrGrAfU(U-I)fU(U-I)fU(U-I)rArAfU(U-I)fCrGfCfCrGfU(U-I)rArGrArArArArGfCrAfU(U-I)rGfU(U-I)fCrArArArGfCfCrGrGrArAfCfCrGfU(U-I)fCfC-3'.

Synthesis of MinE07Lib Aptamer Library. -OH modified TG-beads (0.0087 g) were added to a synthesiser column. This was enough to synthesis 200 copies of every possible sequence. Round 1: 3'-PCLinker-fCfC-5'. This sequence was loaded onto the synthesiser and run on all TG-beads. Round 2: Uridine 1: 3'-fU-5' – The TG-beads were taken out of the main column and split in 4 columns (U-Ph, U-Vi, U, U-I). A synthesis circle using compound **12** was performed on column U-Ph. A synthesis circle using compound **13** was run through column U-Vi. A synthesis circle using compound **14** was run through column U. A synthesis circle using unmodified uridine phosphoramidite was run through column U-I. Round 3: 3'-rGfCfCrArArGrGfCfCrGrArArAfC-5'. This sequence was loaded onto the synthesiser and run on all TG-beads. Round 4: Uridine 2: 3'-fU-5' – The same synthesis procedure was performed as for round 2. Round 5: 3'-rG-5'. This sequence was loaded onto the synthesiser and run on all TG-beads. Round 6: Uridine 3: 3'-fU-5' – The same synthesis procedure was performed as round 2. Round 7: 3'-rAfCrGrArArArArGrA-5'. This sequence was loaded onto the synthesiser and run on all TG-beads. Round 8: Uridine 4: 3'-fU-5' – The same synthesis procedure was performed as round 2. Round 9: 3'-rGfCfCrGfC-5'. This sequence was loaded onto the synthesiser and run on all TG-beads. Round 10: Uridine 5: 3'-fU-5' – The same synthesis procedure was performed as round 2. Round 11: 3'-rArA-5'. This sequence was loaded onto the synthesiser and run on all TG-beads. Round 12: Uridine 6: 3'-fU-5' – The same synthesis procedure was performed as round 2. Round 13: Uridine 7: 3'-fU-5' – The same synthesis procedure was performed as round 2. Round 14: Uridine 8: 3'-fU-5' – The same synthesis procedure was performed as round 2. Round 15: 3'-rArGrGfCrArGrG-5'. This sequence was loaded onto the synthesiser and run on all TG-beads. The weight of the final MinE07Library TG-beads was 0.0029 g.

Table 1. Response values from the trityl monitors for each round of synthesis. Note that the trityl yields in the aptamer library synthesis go up and down during the split and mix depending on the exact portion of TG-beads taken from the total batch.

**Round**	**Nucleotides**	**Response value – qualitative indication of coupling efficiency**
1	3'-PCLinker-fCfC-5'	9.52×10^5
2	3'- fU (U-Ph, U-Vi, U, U-I)-5'	4.95×10^5, 6.04×10^5, 1.60×10^6, 8.49×10^5
3	3'-rGfCfCrArArGrGfCfCrGrArArAfC-5'	1.28×10^6
4	3'- fU (U-Ph, U-Vi, U, U-I)-5'	1.60×10^6, 4.60×10^5, 1.28×10^6, 1.66×10^6
5	3'-rG-5'	2.52×10^5
6	3'- fU (U-Ph, U-Vi, U, U-I)-5'	3.01×10^5, 1.36×10^6, 1.44×10^5, 8.95×10^5
7	3'-rAfCrGrArArArArGrA-5'	1.47×10^6
8	3'- fU (U-Ph, U-Vi, U, U-I)-5'	1.71×10^6, 2.65×10^5, 1.20×10^6, 1.69×10^5
9	3'- rGfCfCrGfC -5'	1.38×10^6
10	3'- fU (U-Ph, U-Vi, U, U-I)-5'	6.07×10^5, 2.59×10^5, 2.65×10^5, 4.29×10^5
11	3'- rArA -5'	1.28×10^6
12	3'- fU (U-Ph, U-Vi, U, U-I)-5'	2.02×10^6, 4.38×10^5, 2.86×10^5, 1.27×10^6
13	3'- fU (U-Ph, U-Vi, U, U-I)-5'	8.95×10^5, 9.21×10^5, 1.54×10^5, 1.58×10^5
14	3'- fU (U-Ph, U-Vi, U, U-I)-5'	3.29×10^5, 6.34×10^5, 1.64×10^5, 7.01×10^5
15	3'-rArGrGfCrArGrG-5'	1.38×10^6

S5. Fluorescence-activated bead sorting

Two-way sort of Accudrop Beads to confirm photo-bleaching theory. Accudrop beads (2 mL) were put into a FACS sized falcon tube. Standard calibration was performed. The drop-delay value was adjusted while viewing BD FACS Accudrop beads in the centre and side sort streams that are illuminated by a red laser. Sort monitoring was undertaken with live video feed of breakoff point, waste collection, and side streams. The accudrop beads were sorted into two gates. The beads that were sorted into positive gate were then put back through the FACS and sorted again in gates. This was repeated again. The 488 nm laser was used with filter 513/17 nm. All data was collected and analysed using the BD FACS Sortware sorter software program.

Two-way sort of 100% 6-carboxyfluorescein tagged microspheres from plain microspheres to confirm photo-bleaching theory. Standard calibration was performed. 6-carboxyfluorescein tagged TG-beads (2 mL) were put into a FACS sized falcon tube. They were sorted into two gates. The microspheres that were sorted into positive were then put back through the FACS and sorted again in gates P7 and P8. This was repeated again. The 488 nm laser was used with filter 513/17 nm.

Sorting MinE07Lib to extract the top binding MinE07 modified aptamers. All of the MinE07Library (1 µM, 0.0029 g) was added to an Eppendorf tube. Binding buffer (100 µL) was added and the tube was incubated at room temperature for 15 minutes. This was centrifuged at 300rpm for 1 minute; the binding buffer was removed and the microspheres were then washed with binding buffer (2 x 100 µL). EGFR-Fc (0.03357 mg/mL, 100 µL) was then added to the tube and this was incubated at room temperature for 60 minutes. This was spun down at 300 rpm for 1 minute, the EGFR-Fc was removed, and the microspheres were then washed with wash buffer (2 x 100 µL). Protein A-FITC (0.03357 mg/mL, 100 µL) was then added to the tube and this was incubated at room temperature for 60 minutes. This was spun down at 300 rpm for 1 minute, the Protein A-FITC was removed, and the microspheres were then washed with wash buffer (2 x 100 µL). Sheath fluid (4 mL) was added, and the microspheres sonicated. The sample was then added to a FACS sized falcon tube. Standard calibration was performed. The MinE07Lib-EGFR-Fc-Protein-A-FITC microspheres sample was two-way sorted three times, changing the gates each time to match the photobleaching and to select less each time. The FACS was calibrated using the 96 well plate set up program along with the accudrop beads. The top microspheres selected from the first three rounds of sorting the MinE07Lib-EGFR-Fc-Protein-A-FITC microsphere were then sorted across two 96 well plates with 100 µL of RO water. The 488 nm laser was used with filter 513/17 nm. All data was collected and analysed using the BD FACS Sortware sorter software program.

S6. Sequencing by Tandem Mass Spectrometry

Photocleaving the top hit MinE07Library aptamers. Both 96 well plates containing the top MinE07Library hits in was placed under a UV lamp for 3 hours to photocleave the aptamers from the TG-beads. The contents of each well (100 µL) was then transferred into individual PCR tubes and stored at 4˚C.

LC-MS/MS of MinE07-Biotin. Mobile phase A is 8 mM tetraethyl ammonium bromide (TEAB) in LC-MS grade water adjusted to the pH 7.5-7.8. Mobile phase B is 8 mM TEAB in a 1:1 ratio of LC-MS grade methanol and water. The MinE07-Biotin samples are dissolved in 10 µL of LC-MS water to the concentrations of 1 µM, 0.5 µM, 0.2 µM, 0.1 µM, 0.05 µM, 0.02 µM and 0.01 µM. A was initially held at 92% and B at 8%. B was increased to 65% at 30 mins and up to 95% at 31 mins. At 33 mins B was dropped back to 8% and then held until 60 mins. The column was maintained at 50˚C.[2, 3]

LC-MS/MS of Hits from MinE07Library Selection. Electrospray mass spectra were recorded on a Bruker micrOTOF-Q II mass spectrometer. The samples were analysed by injecting 2 µL of 0.1 mg/mL solutions into a flowing stream of 95% methanol, 10 mM ammonium acetate at a flow rate of 20 µL/min. The samples were analysed in both positive and negative ion modes. All the masses are mono isotopic and lock mass corrected and therefore should be within 5 ppm of the calculated mass. The samples were purified with MicroSpin™ G-50 columns. Mobile phase A was 8 mM tetraethyl ammonium bromide (TEAB) in LC-MS grade water adjusted to the pH 7.5-7.8. Mobile phase B was 8 mM TEAB in a 1:1 ratio of LC-MS grade methanol and water. The MinE07Library samples were in nuclease free water at an unknown concentration. Initially A was held at 92% and B at 8%. At 30 mins B was increased to 65% and up to 95% at 31 mins. At 33 mins B was dropped back to 8% and then held until 60 mins. The column is maintained at 50˚C.[2,3] 15 MinE07Lib hit were randomly chosen to be analysed.

Analysis of top Aptamers LC-MS/MS data. The data generated by LC-MS/MS of the top hit aptamers MinE07UA, MinE07UB and MinE07UC was analysed using the program RoboOligo.[8] Unlabelled peaks in the MS/MS graphs correspond to fragments that are less than 4 nucleotides long, and so could correspond to multiple places along the chain, or are too close to the limit of detection to be considered as accurate

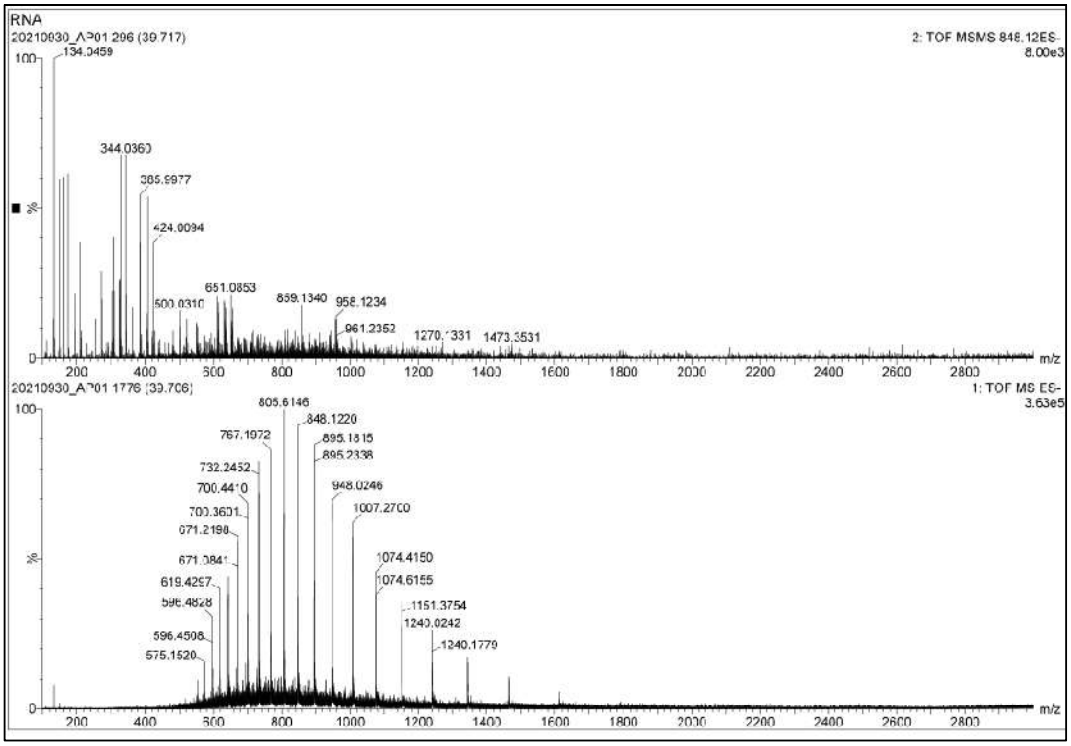

Figure 46. LC-MS/MS spectrum of **MinE07-Biotin** at 10 µM.

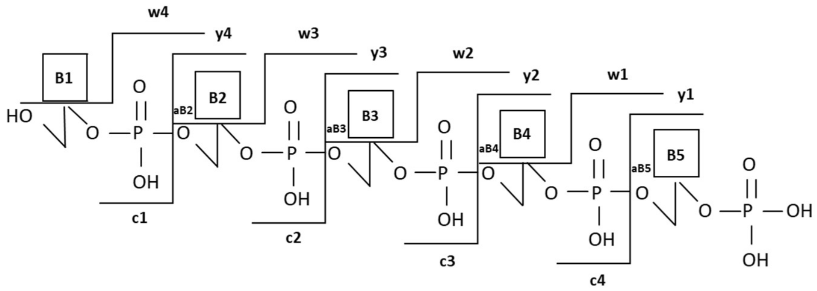

Figure 47. RNA oligomer fragmentation products produced by collision induced dissociation showing the most abundant fragment ions c- and y- fragments as well as w- and a-b- ions.

Fragment 1 of MinE07-Biotin

5'-Biotin- **| rGrGrAfCrGrG |** rAfUfUfUrArAfUfCrGfCfCrGfUrArGrArArArArGfCrAfUrGfUfCrArArArGfCfCrGrGrA rAfCfCrGfUfCfC-3'

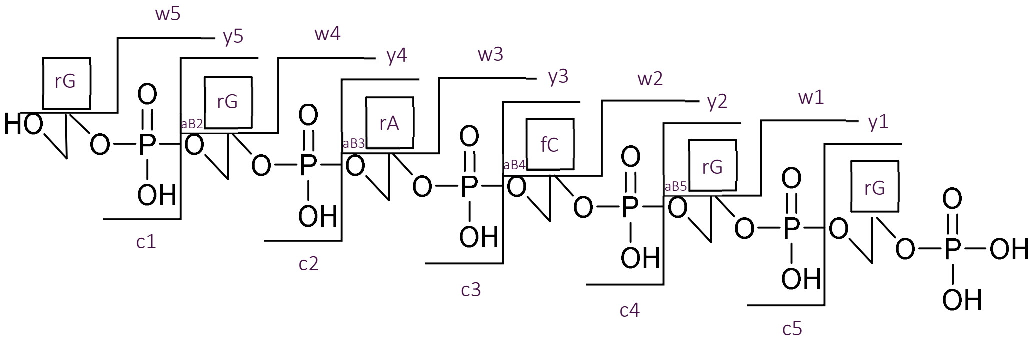

Figure 48. Fragment 1 of **MinE07-Biotin** 5'-3'.

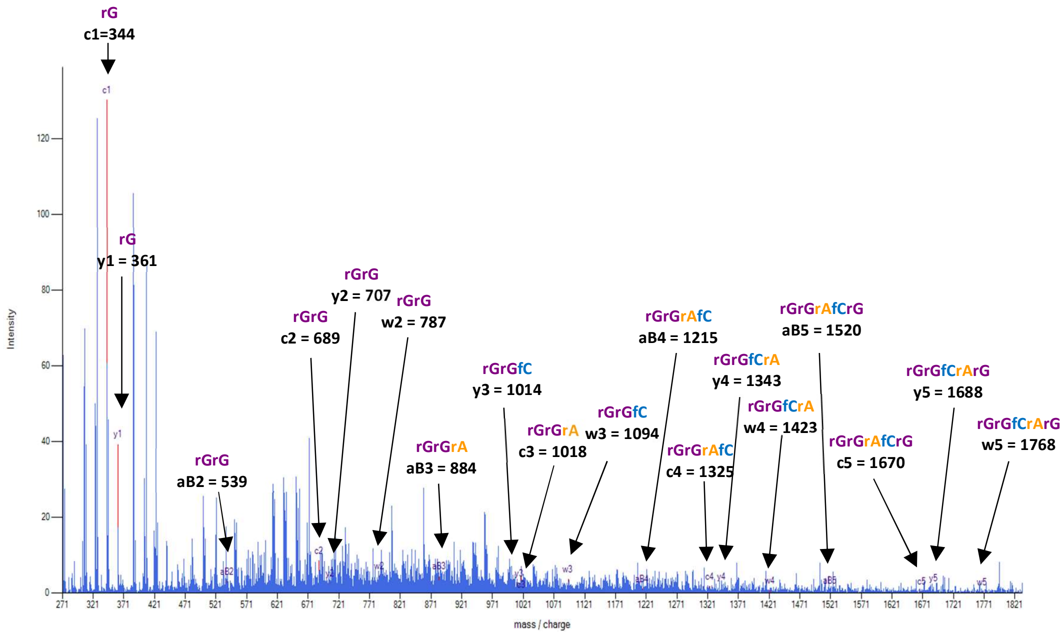

Figure 49. LC-MS/MS Fragment 1 Data of **MinE07-Biotin**.

Fragment 2 of MinE07-Biotin

5'-Biotin-rGrGrAfCr **| GrGrAfUfUfU |** rArAfUfCrGfCfCrGfUrArGrArArArArGfCrAfUrGfUfCrArArArGfCfCrGrGrA rAfCfCrGfUfCfC-3'

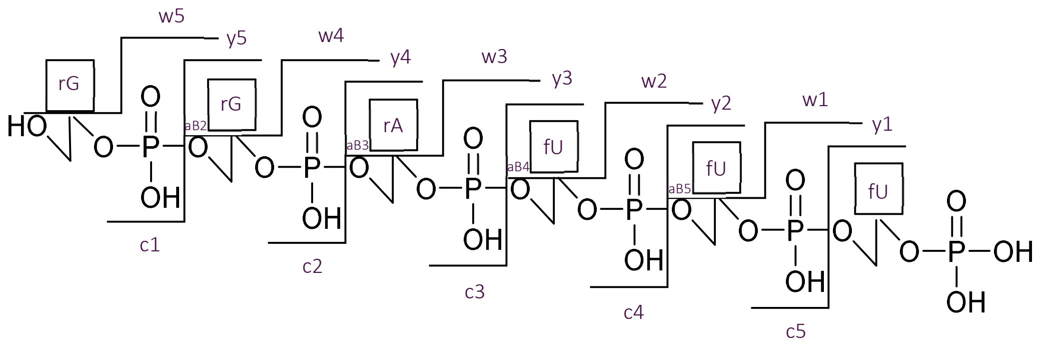

Figure 50. Fragment 2 of **MinE07-Biotin** 5'-3'.

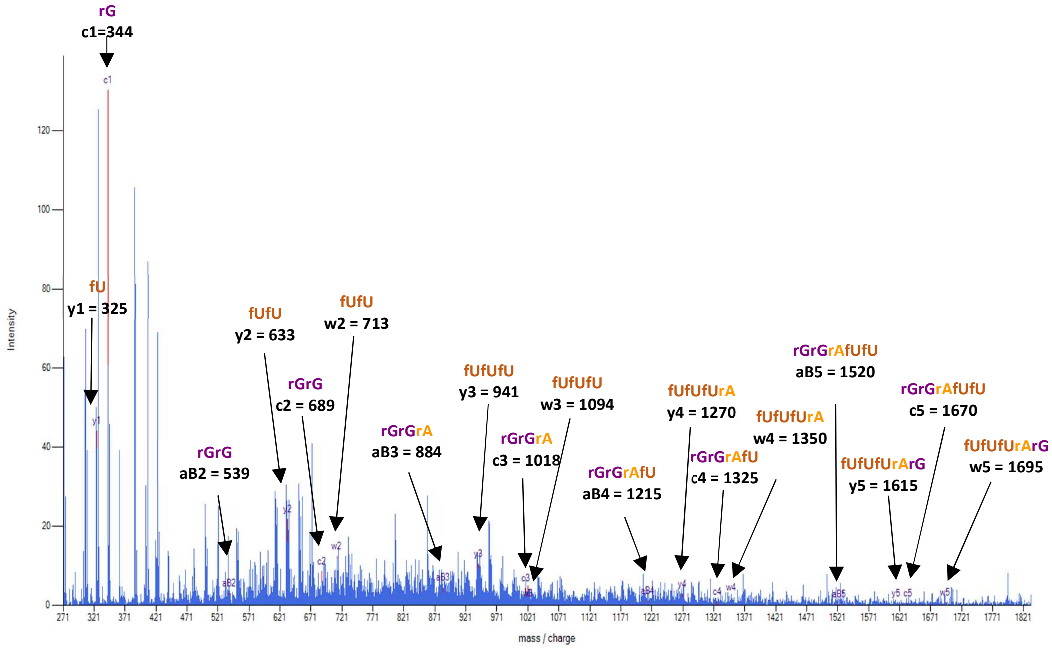

Figure 51. LC-MS/MS Fragment 2 Data of **MinE07-Biotin**.

Fragment 3 of MinE07-Biotin

5'-Biotin-rGrGrAfCrGrGrAfUfUfUrA **| rAfUfCrGfCfC |** rGfUrArGrArArArArGfCrAfUrGfUfCrArArArGfCfCrGrGrA rAfCfCrGfUfCfC-3'

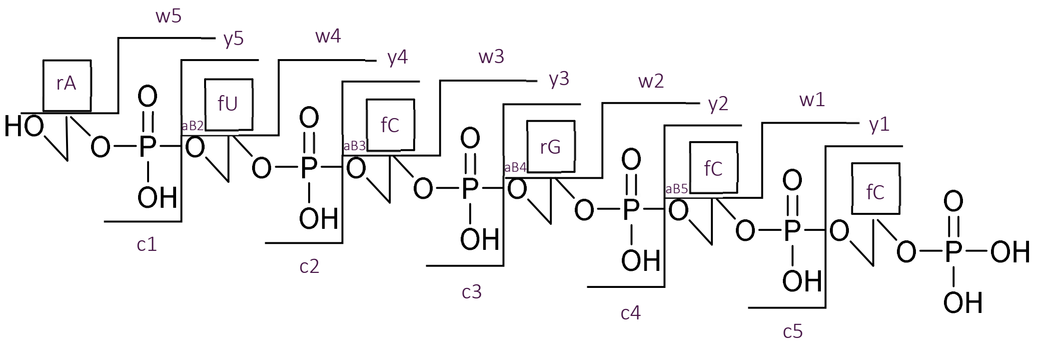

Figure 52. Fragment 3 of **MinE07-Biotin** 5'-3'.

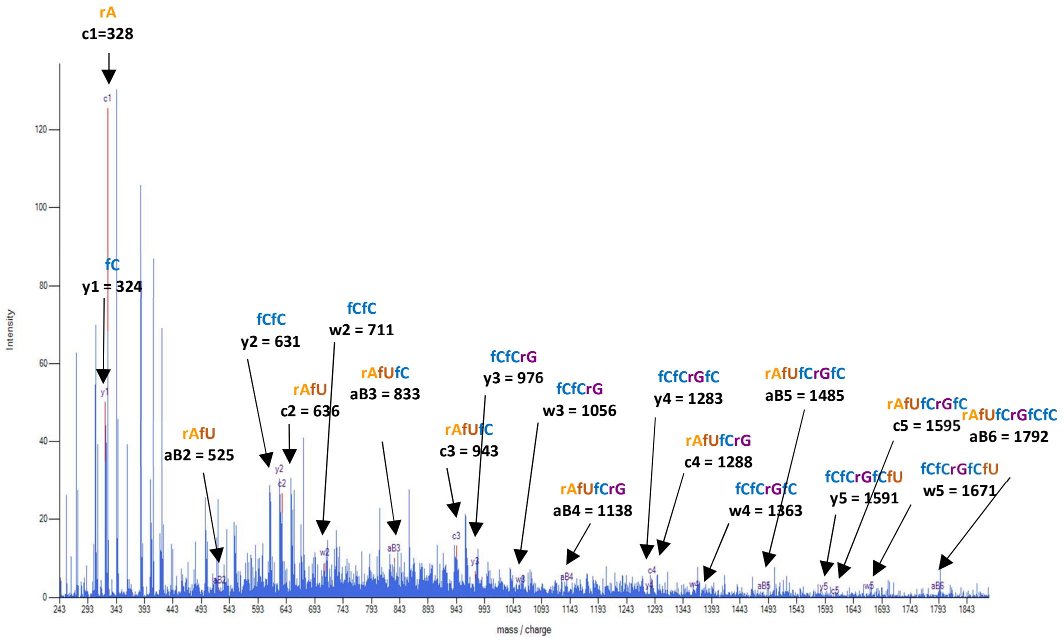

Figure 53. LC-MS/MS Fragment 3 Data of **MinE07-Biotin**.

Fragment 4 of MinE07-Biotin

5'-Biotin-rGrGrAfCrGrGrAfUfUfUrArAfUfCrGfCfC | **rGfUrArGrArArA** | rArGfCrAfUrGfUfCrArArArGfCfCrGrGrArAfCfCrGfUfCfC-3'

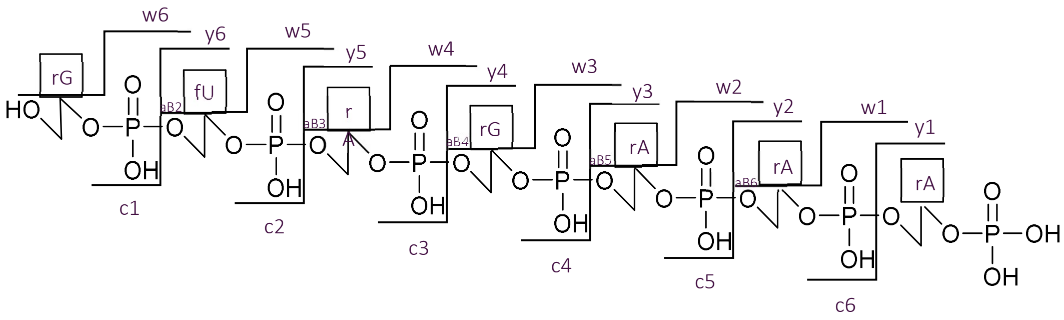

Figure 54. Fragment 4 of **MinE07-Biotin** 5'-3'.

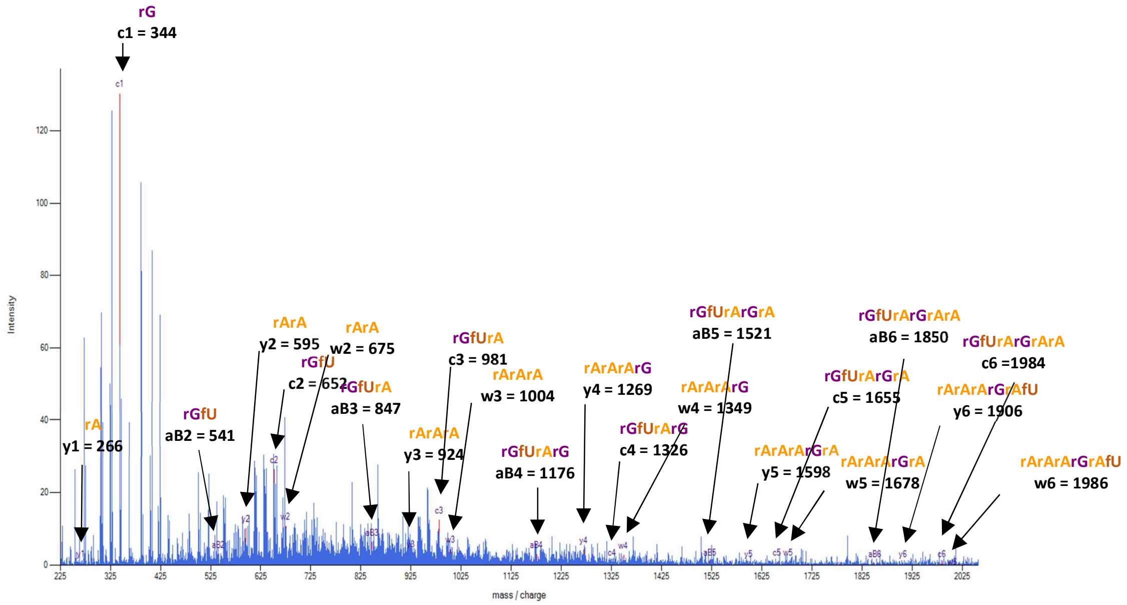

Figure 55. LC-MS/MS Fragment 4 Data of **MinE07-Biotin**.

Fragment 5 of MinE07-Biotin

5’-Biotin-rGrGrAfCrGrGrAfUfUfUrArAfUfCrGfCfCrGfUrArG **| rArArArArGfC |** rAfUrGfUfCrArArArGfCfCrGrGrArAfCfCrGfUfCfC-3’

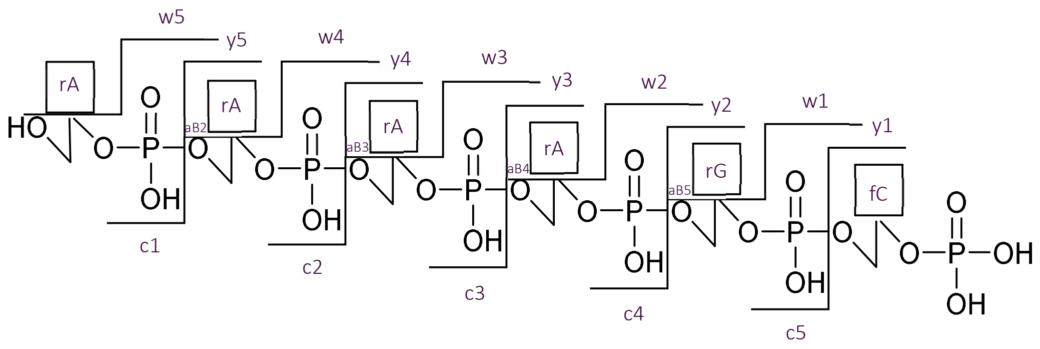

Figure 56. Fragment 5 of MinE07-Biotin 5’-3’.

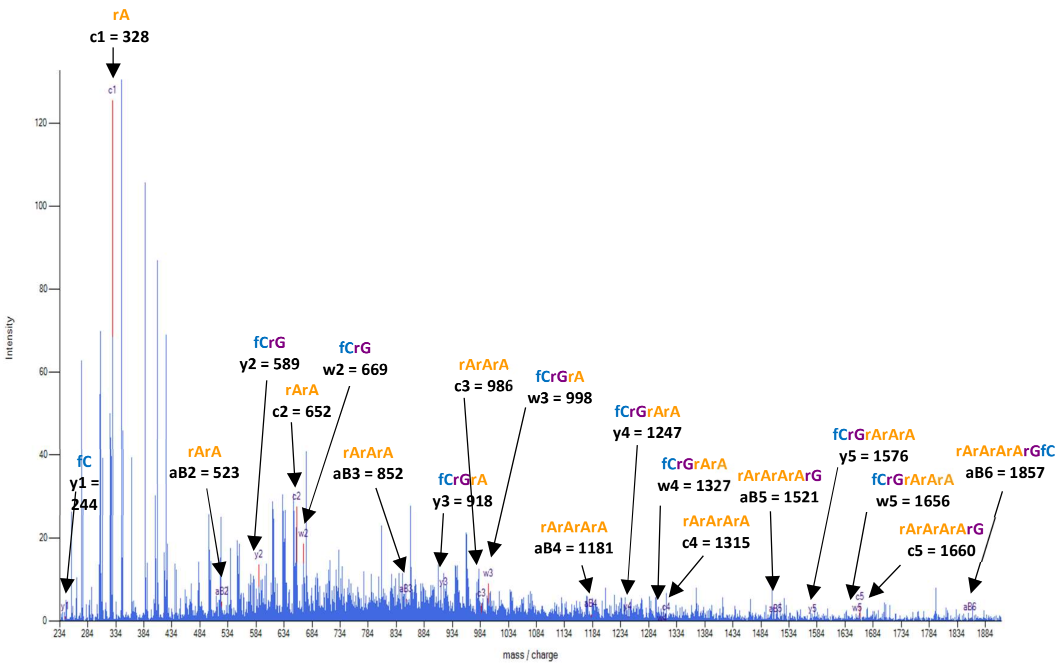

Figure 57. LC-MS/MS Fragment 5 Data of **MinE07-Biotin**.

Fragment 6 of MinE07-Biotin

5'-Biotin-rGrGrAfCrGrGrAfUfUfUrArAfUfCrGfCfCrGfUrArGrArArArA **| rGfCrAfUrG |** fUfCrArArArGfCfCrGrGrA rAfCfCrGfUfCfC-3'

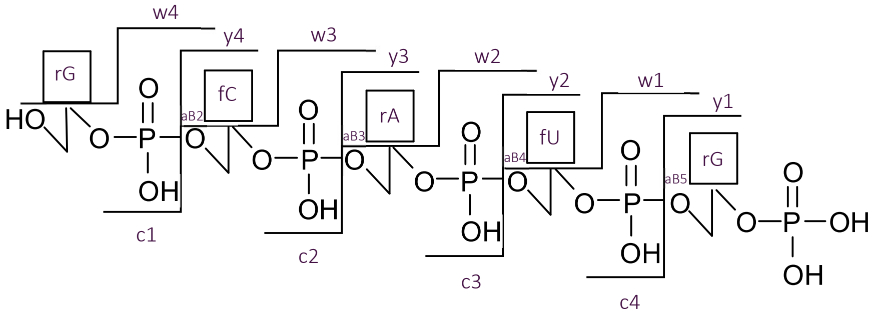

Figure 58. Fragment 6 of **MinE07-Biotin** 5'-3'.

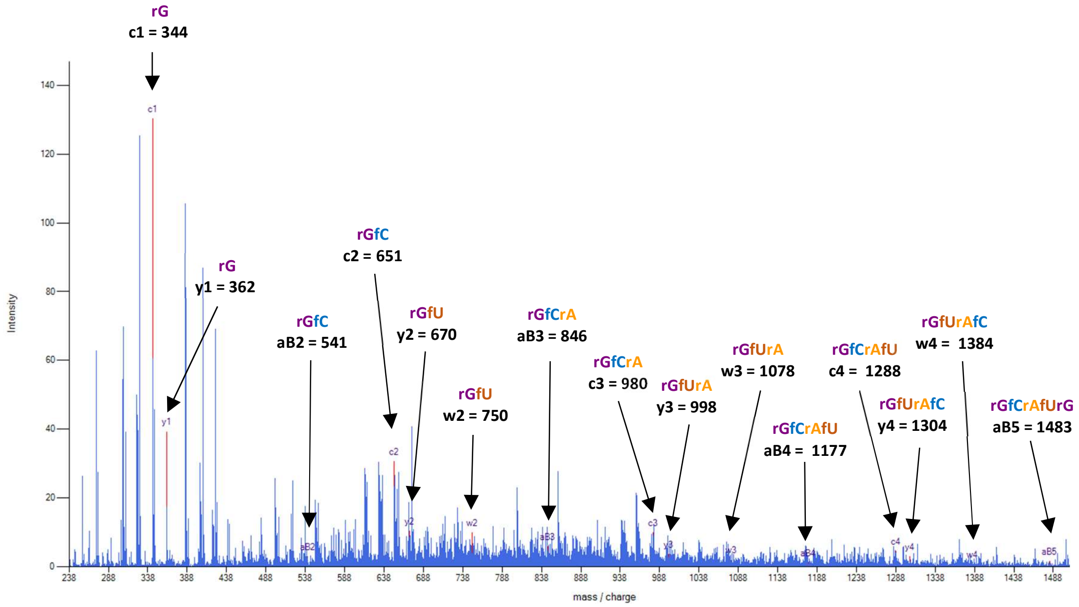

Figure 59. LC-MS/MS Fragment 6 Data of **MinE07-Biotin**.

Fragment 7 of MinE07-Biotin

5'-Biotin-rGrGrAfCrGrGrAfUfUfUrArAfUfCrGfCfCrGfUrArGrArArArArGfCrAfU **| rGfUfCrArArArG |** fCfCrGrGrArAfCfCrGfUfCfC-3'

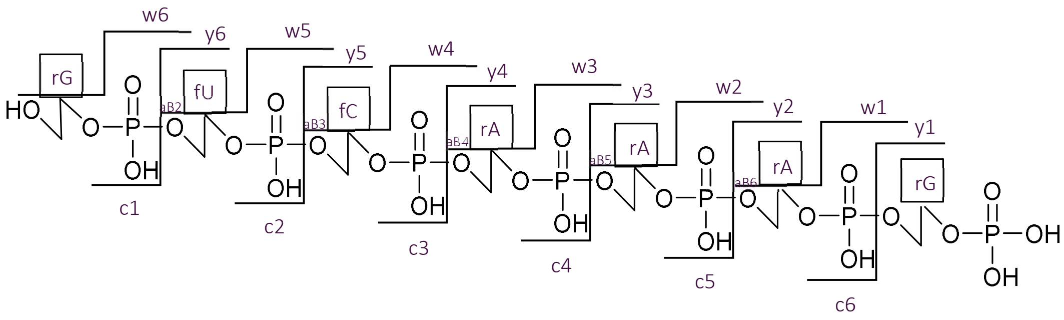

Figure 60. Fragment 7 of **MinE07-Biotin** 5'-3'.

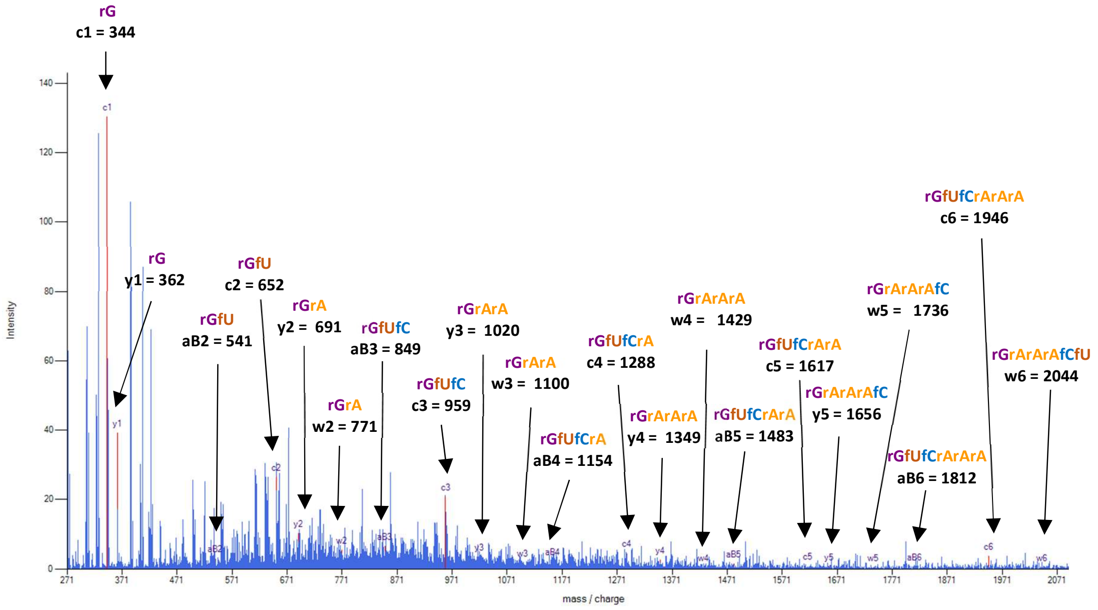

Figure 61. LC-MS/MS Fragment 7 Data of **MinE07-Biotin**.

Fragment 8 of MinE07-Biotin

5'-Biotin-rGrGrAfCrGrGrAfUfUfUrArAfUfCrGfCfCrGfUrArGrArArArArGfCrAfUrGfUfCrA **| rArArGfCfCrG |** rGrArAfCfCrGfUfCfC-3'

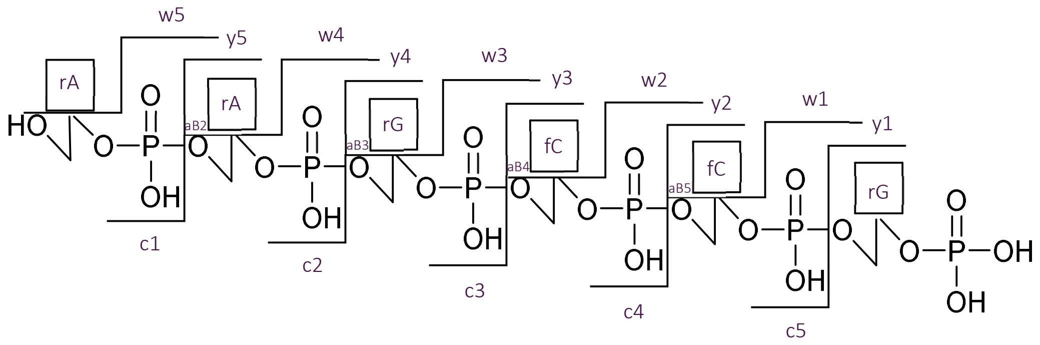

Figure 62. Fragment 8 of **MinE07-Biotin** 5'-3'.

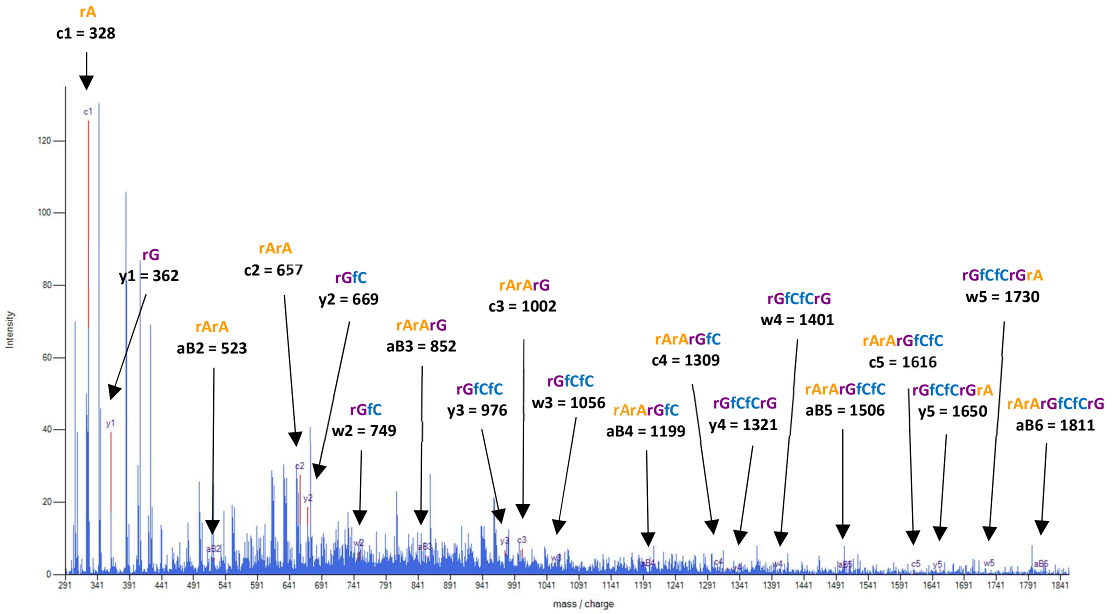

Figure 63. LC-MS/MS Fragment 8 Data of **MinE07-Biotin**.

Fragment 9 of MinE07-Biotin

5'-Biotin-rGrGrAfCrGrGrAfUfUfUrArAfUfCrGfCfCrGfUrArGrArArArArGfCrAfUrGfUfCrArArArG **| fCfCrGrGrArA |** fCfCrGfUfCfC-3'

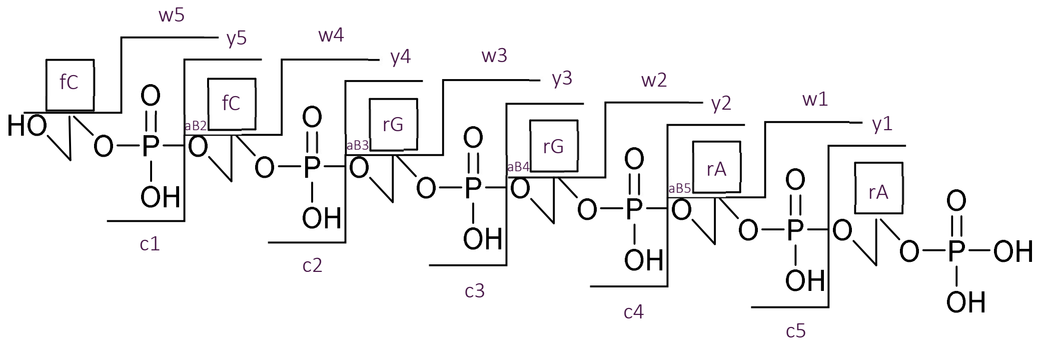

Figure 64. Fragment 9 of **MinE07-Biotin** 5'-3'.

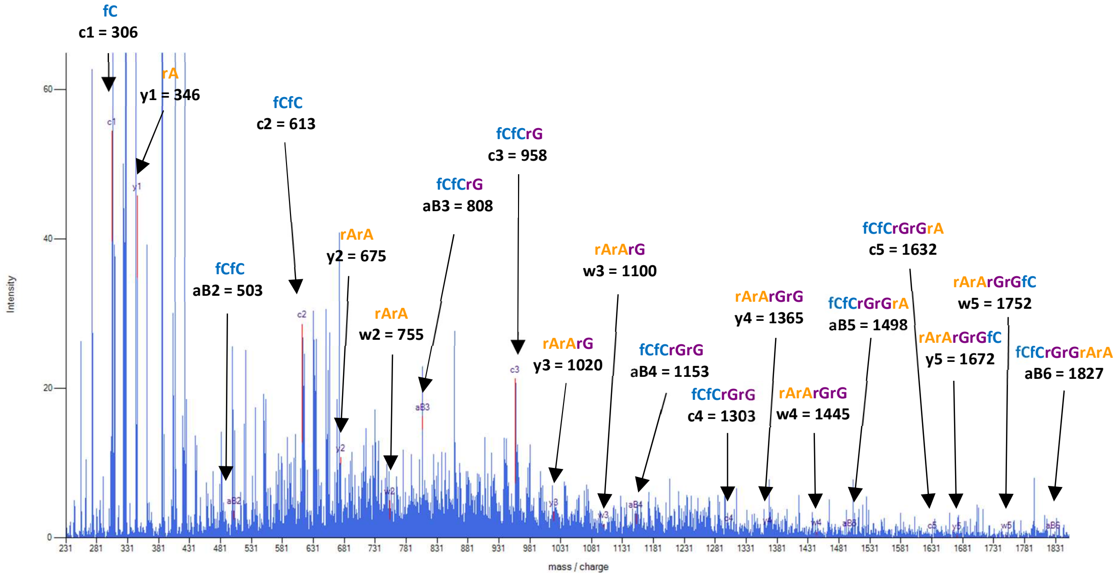

Figure 65. LC-MS/MS Fragment 9 Data of **MinE07-Biotin**.

Fragment 10 of MinE07-Biotin

5’-Biotin-rGrGrAfCrGrGrAfUfUfUrArAfUfCrGfCfCrGfUrArGrArArArArGfCrAfUrGfUfCrArArArGfCfC **| rGrGrArA fCfC |** rGfUfCfC-3’

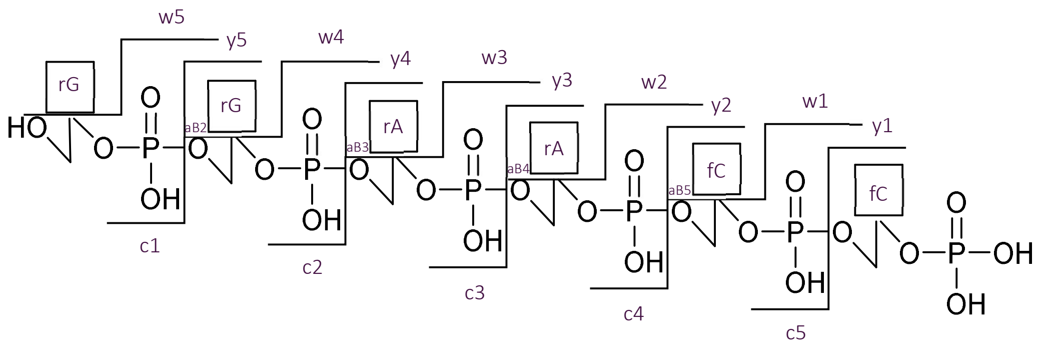

Figure 66. Fragment 10 of **MinE07-Biotin** 5’-3’.

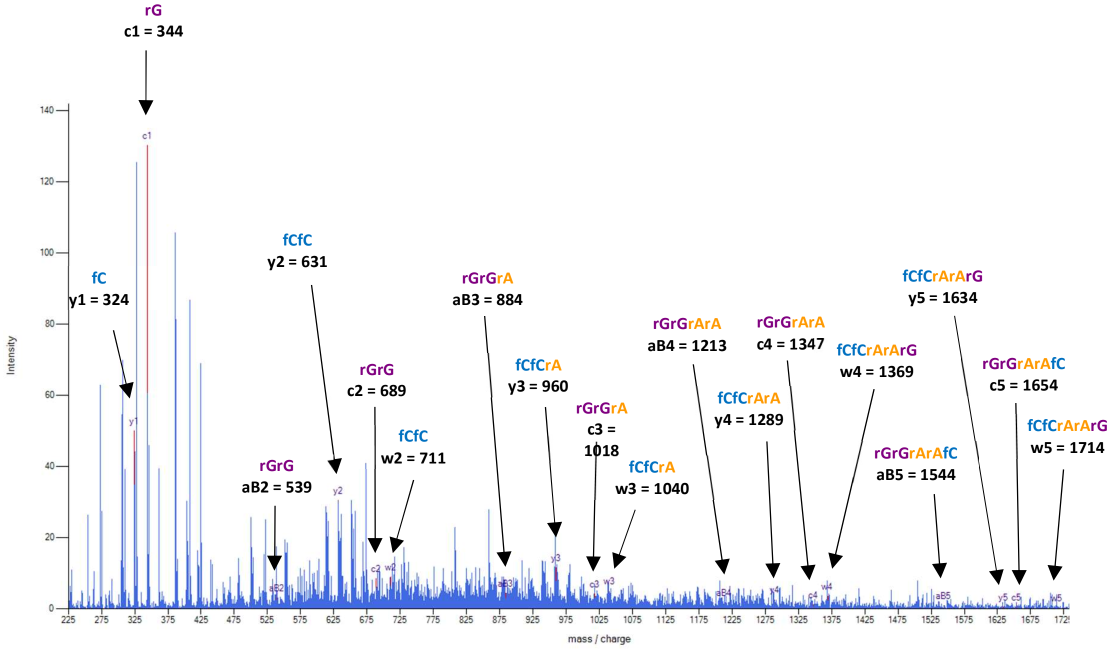

Figure 67. LC-MS/MS Fragment 10 Data of **MinE07-Biotin**.

Fragment 11 of MinE07-Biotin

5'-Biotin-rGrGrAfCrGrGrAfUfUfUrArAfUfCrGfCfCrGfUrArGrArArArArGfCrAfUrGfUfCrArArArGfCfCrGrGrArA **|** **fCfCrGfUfCfC |**-3'

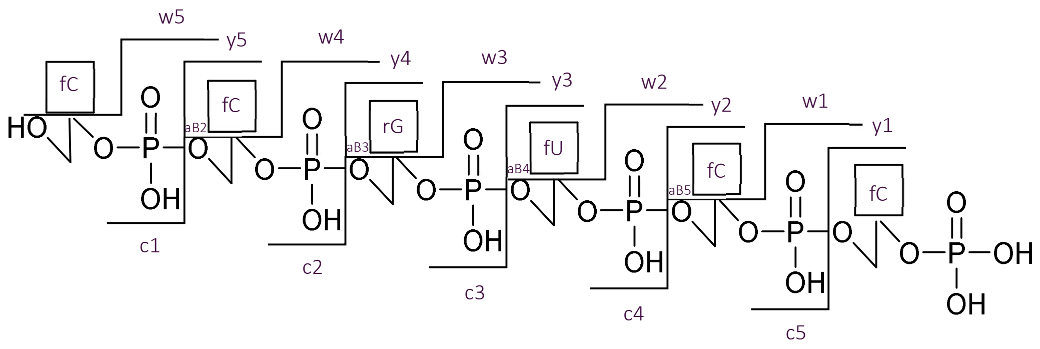

Figure 68. Fragment 11 of **MinE07-Biotin** 5'-3'.

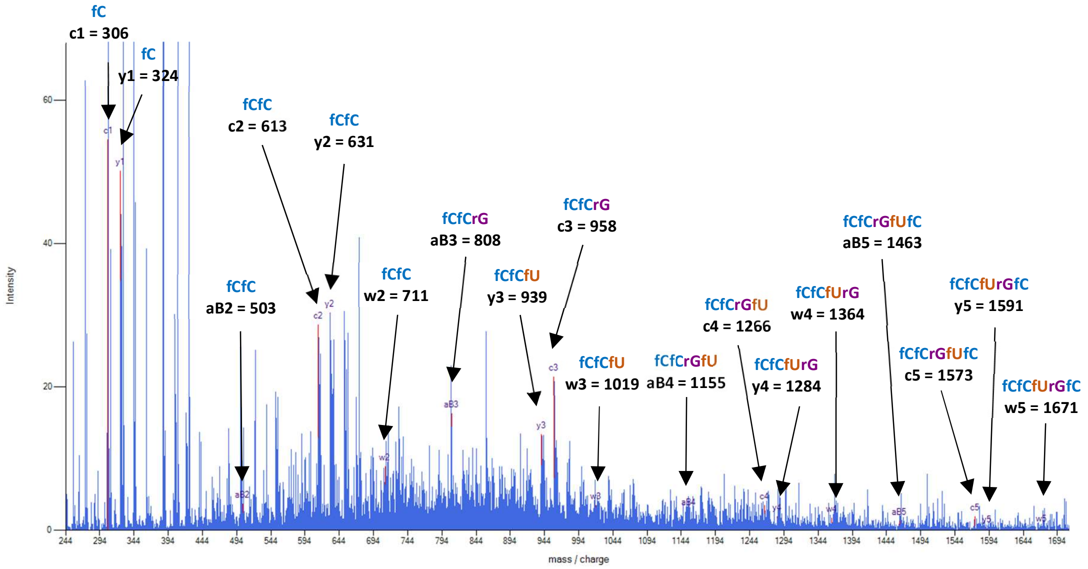

Figure 69. LC-MS/MS Fragment 11 Data of **MinE07-Biotin**.

LC-MS/MS Analysis of MinE07UA

MinE07UA: 5’-fCrGrGrAfUfU-PhfU-PhrArAfUfCrGfCfCrGfU-IrArGrArArArArGfCrAfUrGfUfCrArArArGfCfCrGrGrArAfCfCrGfU-PhfCfC-PCL-3’

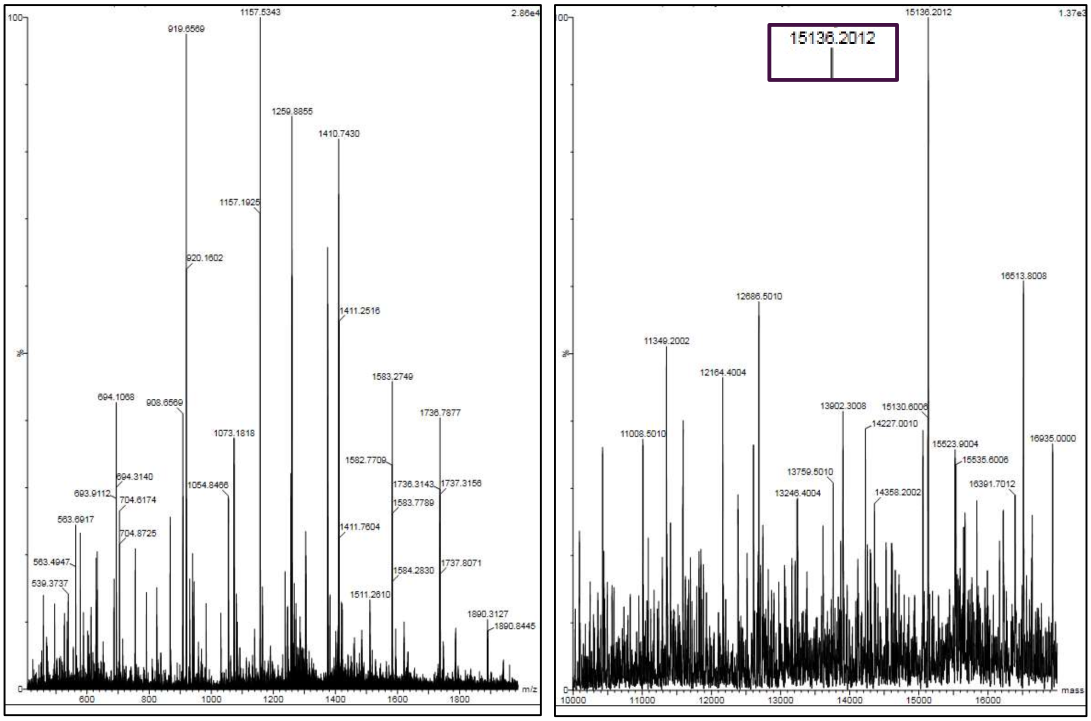

Figure 70. Mass spectrum of **MinE07UA** and predicted mass.

Fragment 1 of MinE07UA

5’-fCrGrGrA **| fUfU-PhfU-PhrArA |** fUfCrGfCfCrGfU-IrArGrArArArArGfCrAfUrGfUfCrArArArGfCfCrGrGrArAfCf CrGfU-PhfCfC-PCL-3’

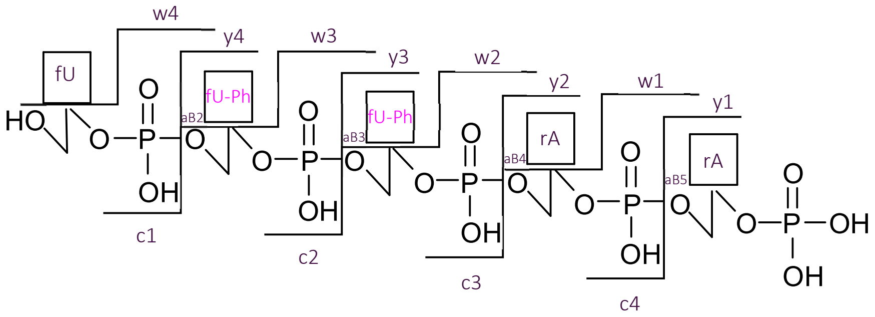

Figure 71. Fragment 1 of **MinE07UA** 5’-3’.

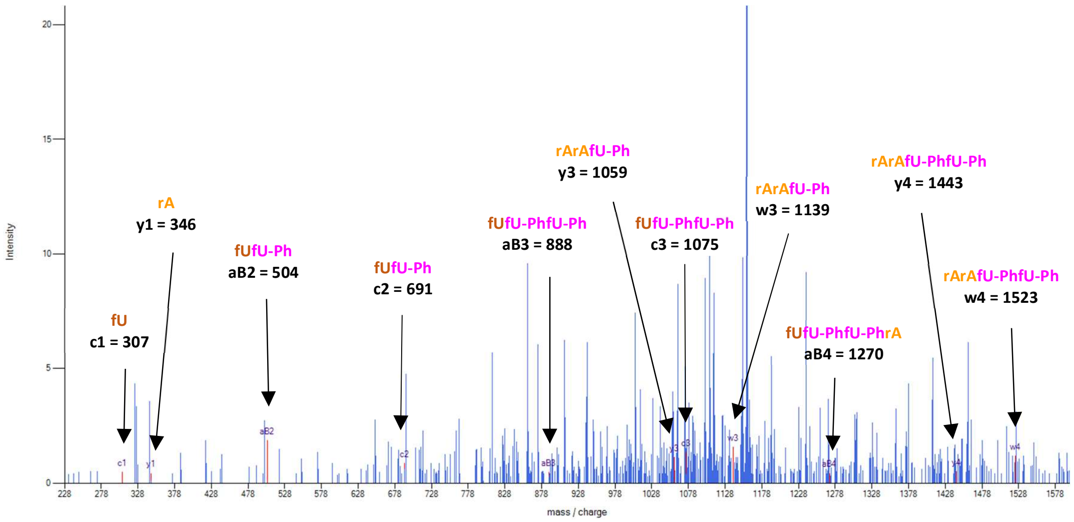

Figure 72. LC-MS/MS Fragment 1 Data of **MinE07UA**.

Fragment 2 of MinE07UA

5'-fCrGrGrAfUfU-Ph | **fU-PhrArAfUfCrG** | fCfCrGfU-IrArGrArArArArGfCrAfUrGfUfCrArArArGfCfCrGrGrArAfCf CrGfU-PhfCfC-PCL-3'

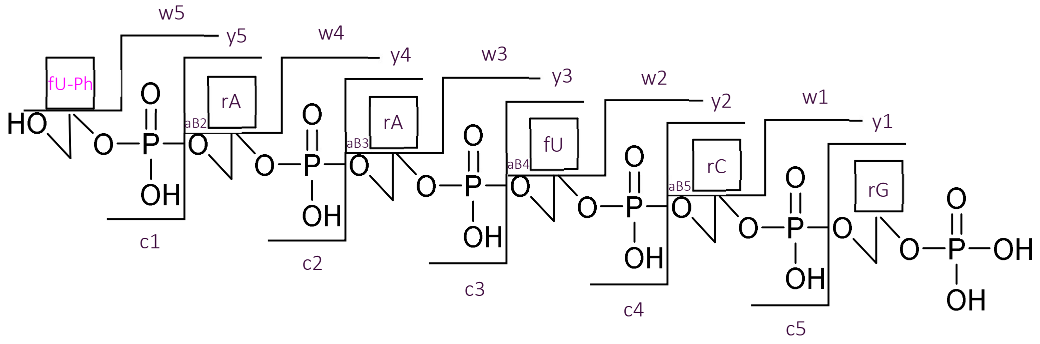

Figure 73. Fragment 2 of **MinE07UA** 5'-3'.

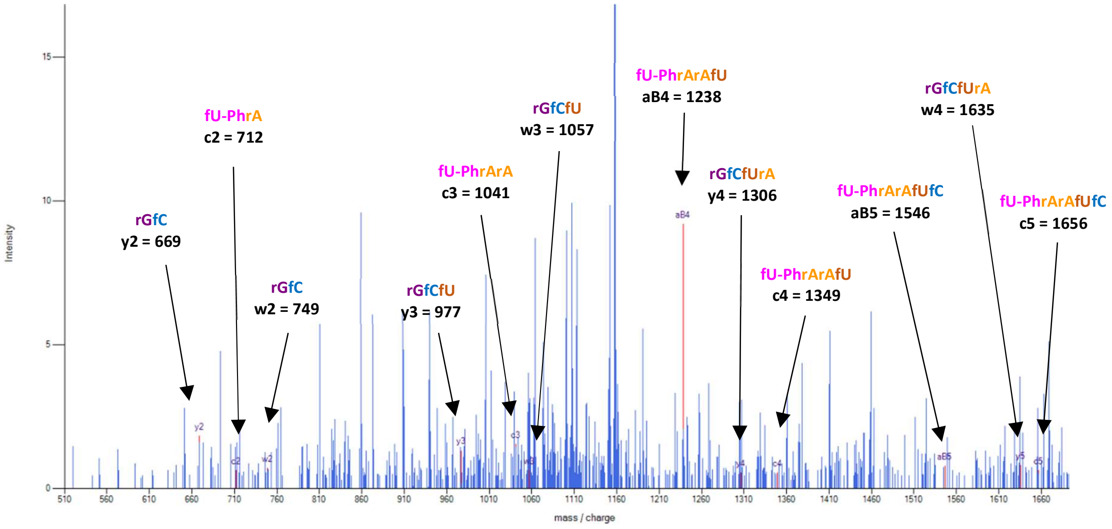

Figure 74. LC-MS/MS Fragment 2 Data of **MinE07UA**.

Fragment 3 of MinE07UA

5'-fCrGrGrAfUfU-PhfU-PhrArA | **fUfCrGfCfC** | rGfU-IrArGrArArArArGfCrAfUrGfUfCrArArArGfCfCrGrGrAfCfCrGfU-PhfCfC-PCL-3'

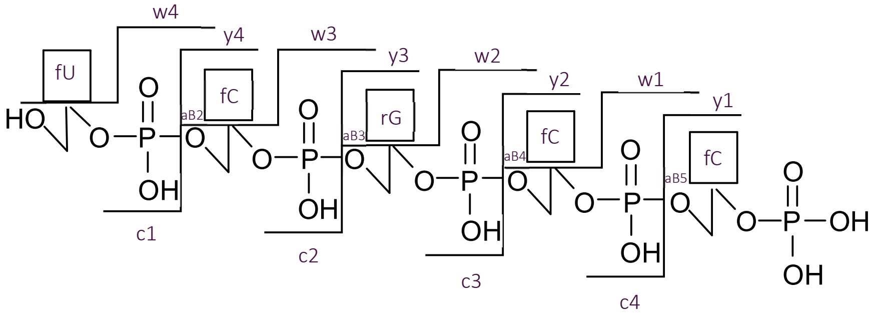

Figure 75. Fragment 3 of **MinE07UA** 5'-3'.

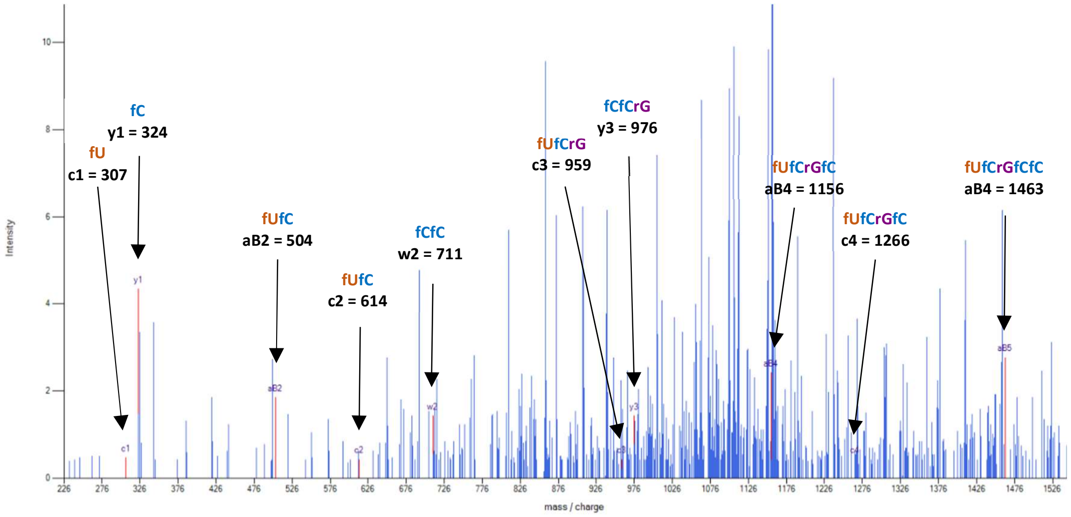

Figure 76. LC-MS/MS Fragment 3 Data of **MinE07UA**.

Fragment 4 of MinE07UA

5'-fCrGrGrAfUfU-PhfU-PhrArAfUfC | **rGfCfCrGfU-IrA** | rGrArArArArGfCrAfUrGfUfCrArArArGfCfCrGrGrArAfCf CrGfU-PhfCfC-PCL-3'

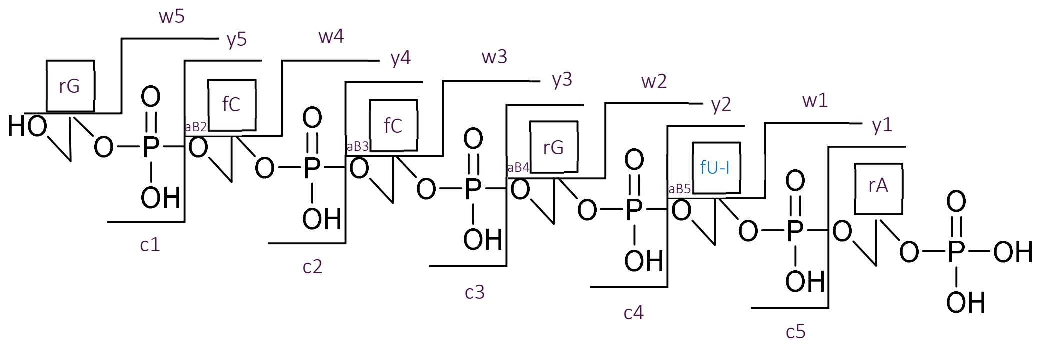

Figure 77. Fragment 4 of **MinE07UA** 5'-3'.

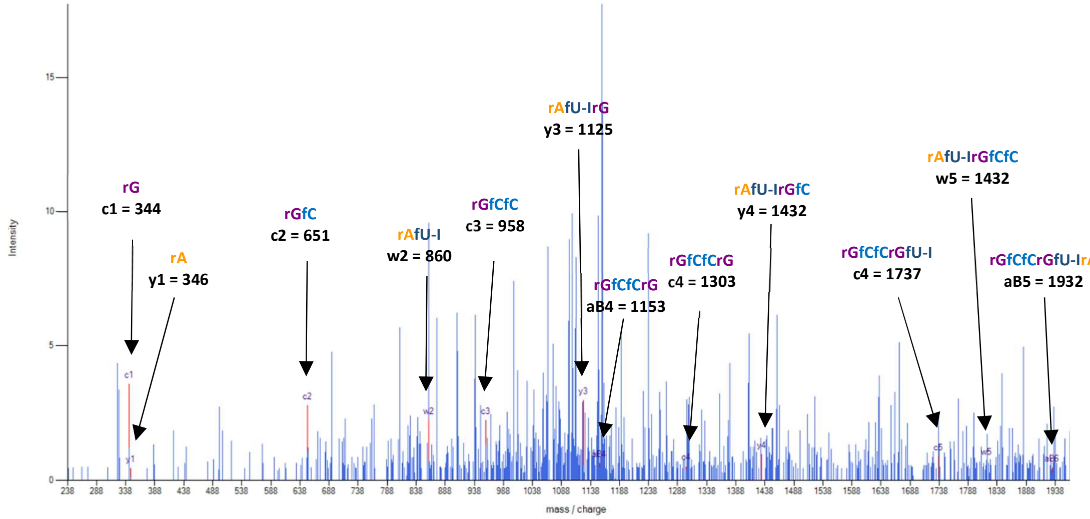

Figure 78. LC-MS/MS Fragment 4 Data of **MinE07UA**.

Fragment 5 of MinE07UA

5'-fCrGrGrAfUfU-PhfU-PhrArAfUfCrGfCfCrGfU-IrArGrArArArArGfCrA | **fUrGfUfCrA** | rArArGfCfCrGrGrArAfCf CrGfU-PhfCfC-PCL-3'

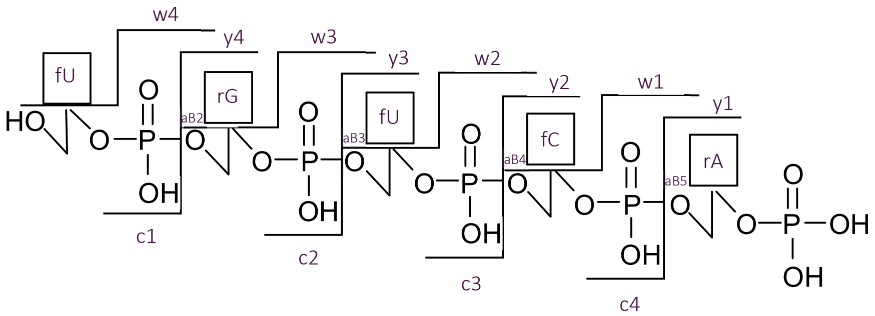

Figure 79. Fragment 5 of **MinE07UA** 5'-3'.

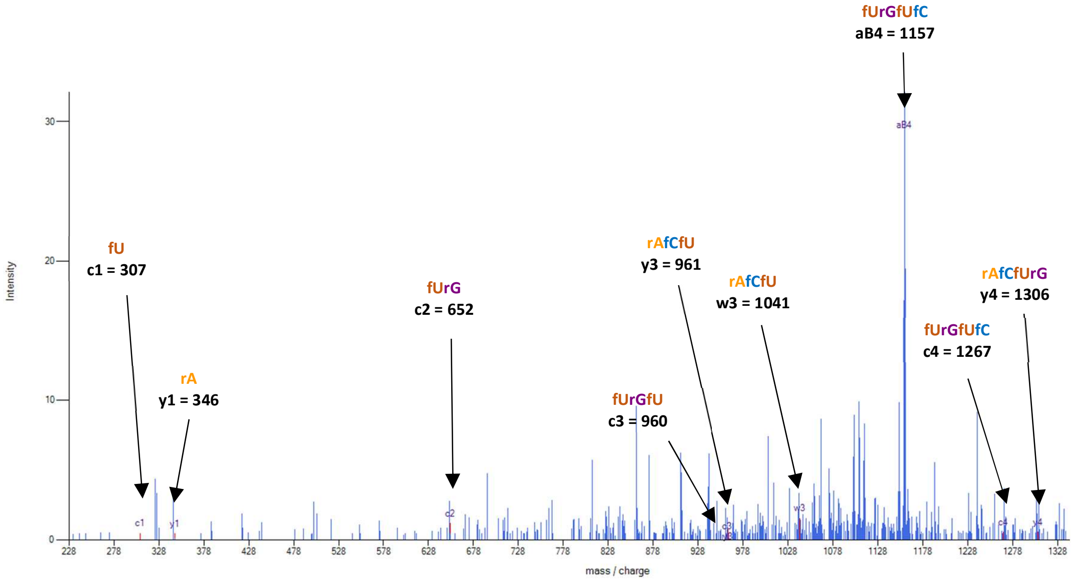

Figure 80. LC-MS/MS Fragment 5 Data of **MinE07UA**.

Fragment 6 of MinE07UA

5’-fCrGrGrAfUfU-PhfU-PhrArAfUfCrGfCfCrGfU-IrArGrArArArArGfCrAfUrGfUfCrArArArGfCfCrG **|rGrArAfCfCr GfU-PhfC |** fC-PCL-3’

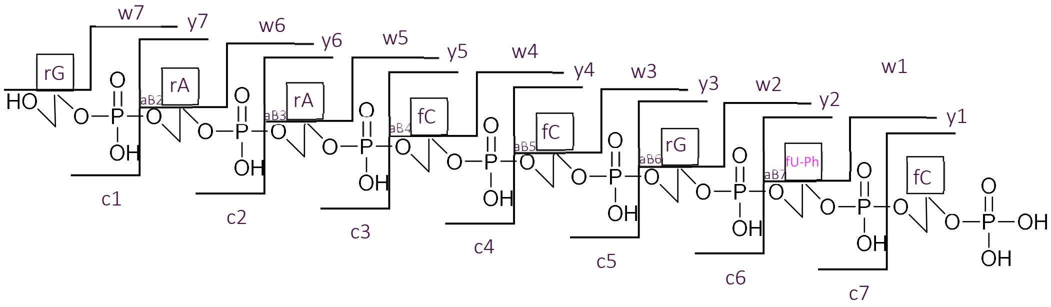

Figure 81. Fragment 6 of **MinE07UA** 5’-3’.

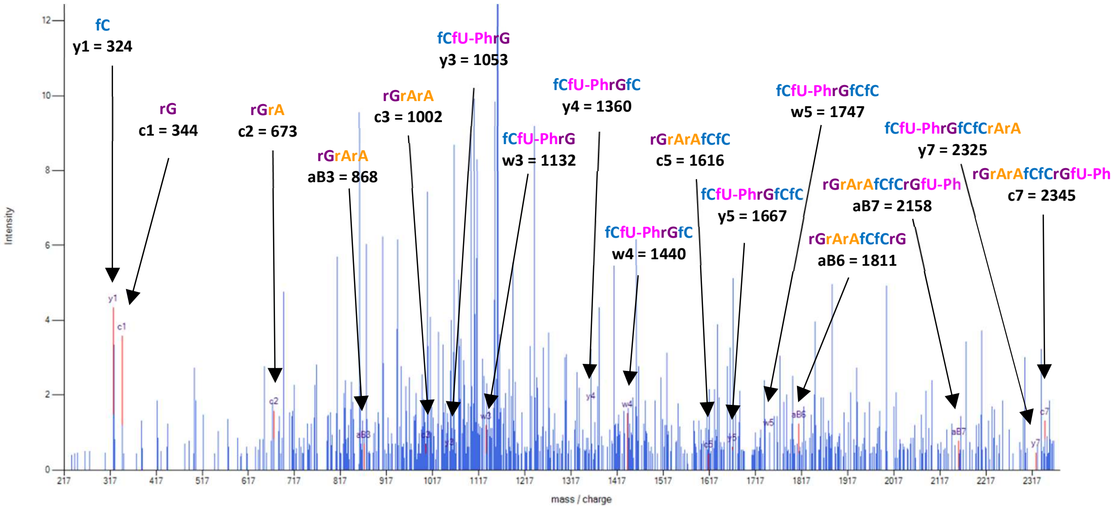

Figure 82. LC-MS/MS Fragment 6 Data of **MinE07UA**.

Fragment 7 of MinE07UA

5'-fCrGrGrAfUfU-PhfU-PhrArAfUfCrGfCfCrGfU-IrArGrArArArArGfCrAfUrGfUfCrArArArGfCfCrGrGrArA | **fCfCrGfU-PhfC** | fC-PCL-3'

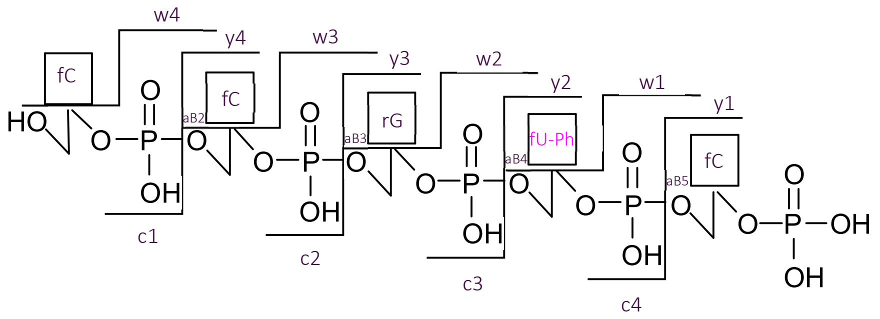

Figure 83. Fragment 7 of **MinE07UA** 5'-3'.

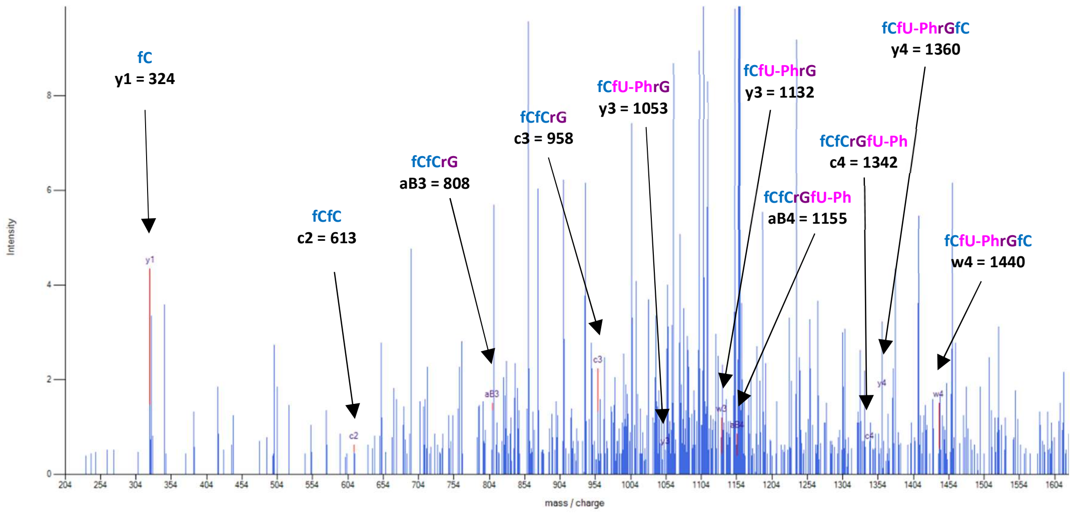

Figure 84. LC-MS/MS Fragment 7 Data of **MinE07UA**.

LC-MS/MS Analysis of MinE07UB

MinE07UB: 5’-rGrGrAfCrGrGrAfUfU-PhfU-PhrArAfU-IfCrGfCfCrGfU-PhrArGrArArArArGfCrAfUrGfUfCrArArAr GfCfCrGrGrArAfCfCrGfUfCfC-PCL-3’

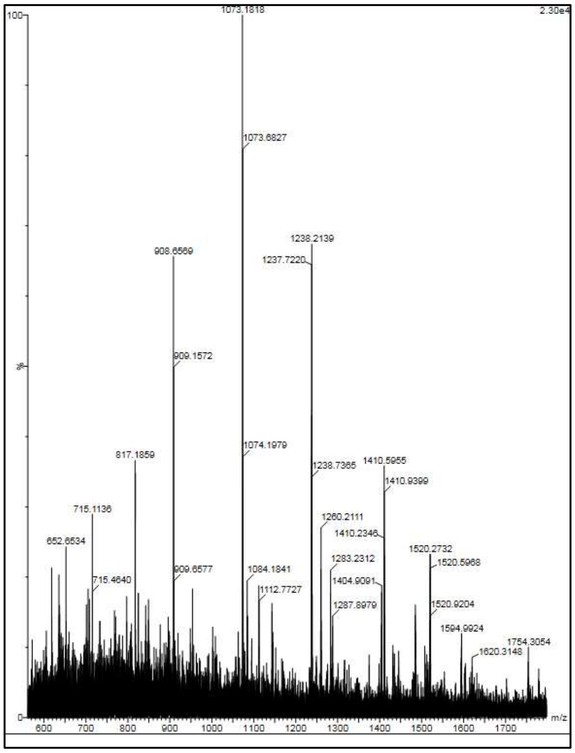

Figure 85. Mass spectrum of **MinE07UB**.

Fragment 1 of **MinE07UB**

5'-rGrGrAfCrGrG **| rAfUfU-PhfU-PhrA |** rAfU-IfCrGfCfCrGfU-PhrArGrArArArArGfCrAfUrGfU-IfCrArArArGfCfCr GrGrArAfCfCrGfUfCfC-PCL-3'

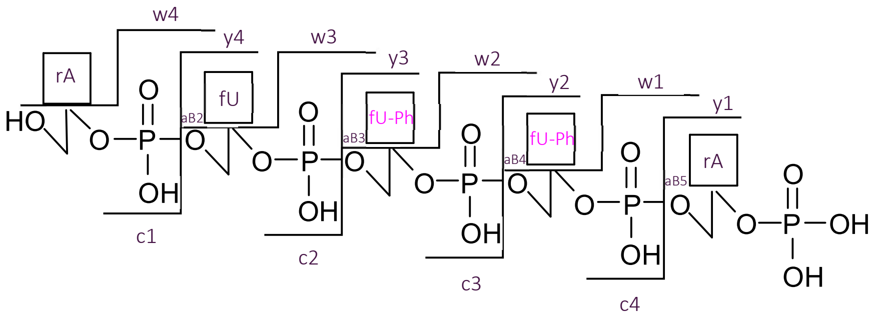

Figure 86. Fragment 1 of **MinE07UB** 5'-3'.

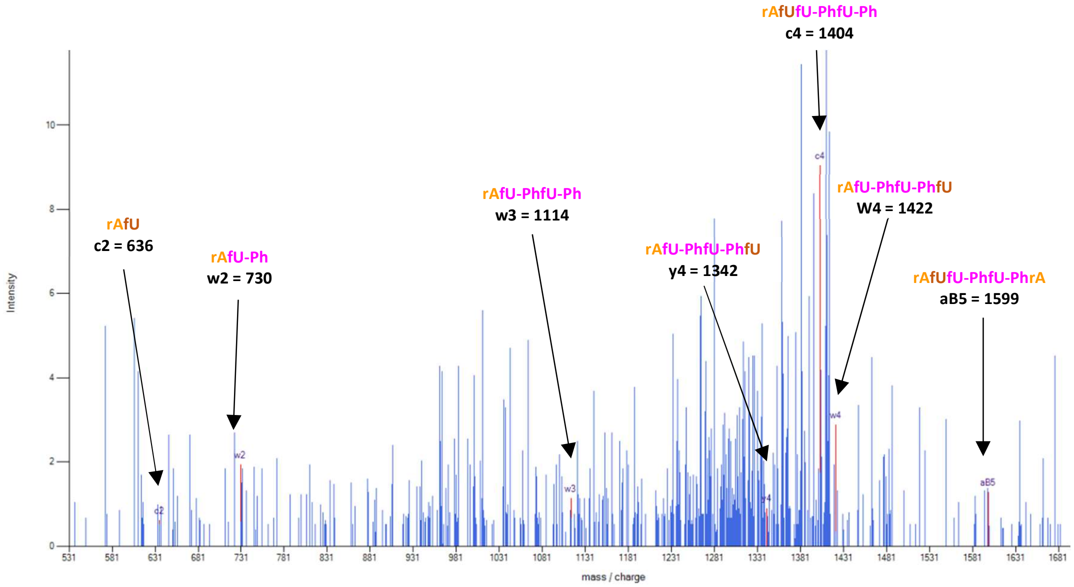

Figure 87. LC-MS/MS Fragment 1 Data of **MinE07UB**.

Fragment 2 of MinE07UB

5'-rGrGrAfCrGrGrA | **fUfU-PhfU-PhrArA** | fU-IfCrGfCfCrGfU-PhrArGrArArArArGfCrAfUrGfU-IfCrArArArGfCfCr GrGrArAfCfCrGfUfCfC-PCL-3'

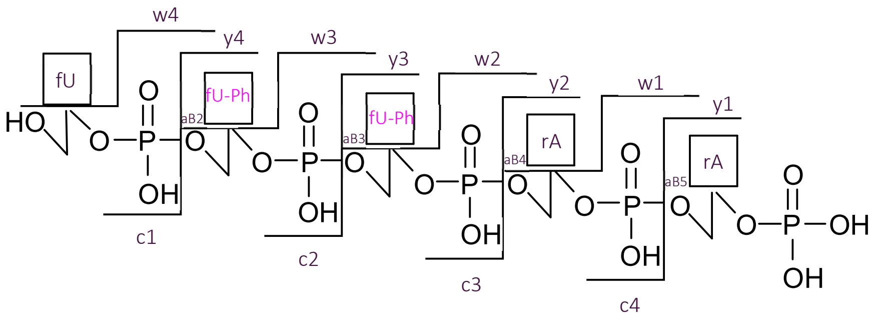

Figure 88. Fragment 2 of **MinE07UB** 5'-3'.

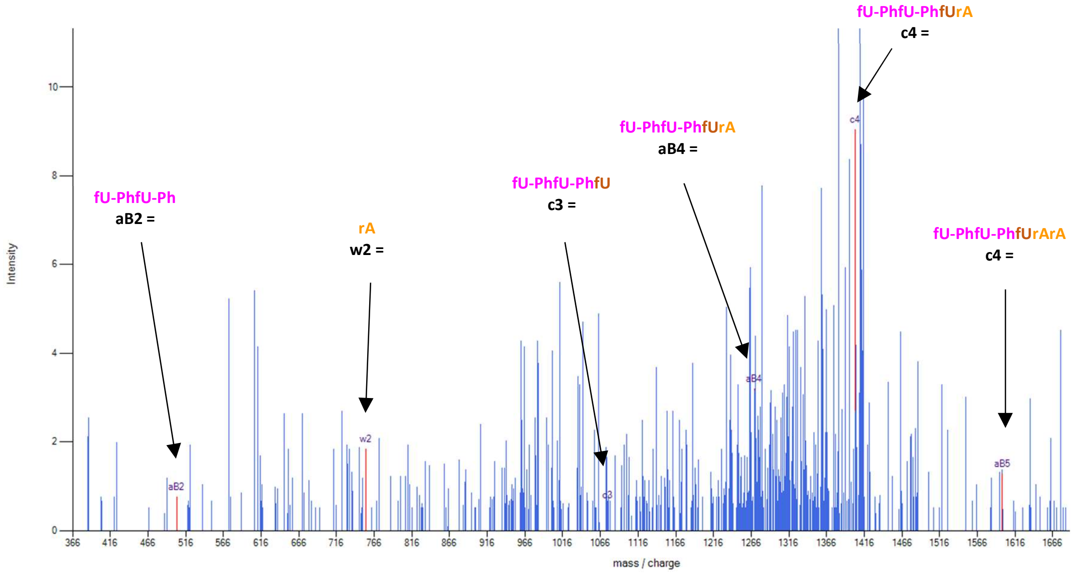

Figure 89. LC-MS/MS Fragment 2 Data of **MinE07UB**.

Fragment 3 of MinE07UB

5'-rGrGrAfCrGrGrAfUfU-PhfU-PhrA | **rAfU-IfCrGfCfCrGfU-PhrA** | rGrArArArArGfCrAfUrGfU-IfCrArArArGfCfC rGrGrArAfCfCrGfUfCfC-PCL-3'

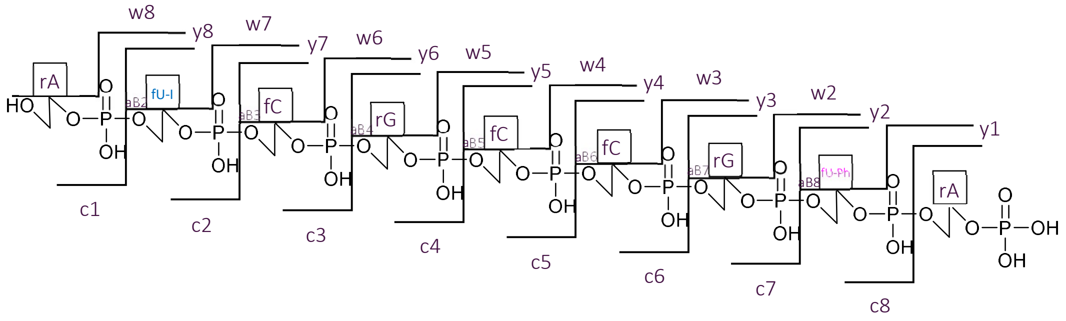

Figure 90. Fragment 3 of **MinE07UB** 5'-3'.

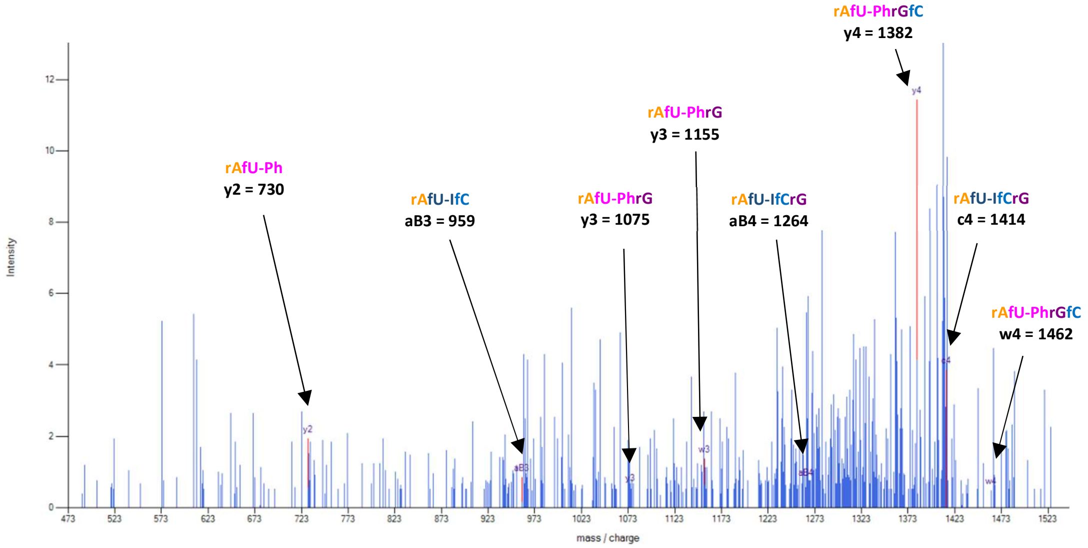

Figure 91. LC-MS/MS Fragment 3 Data of **MinE07UB**.

Fragment 4 of MinE07UB

5'-rGrGrAfCrGrGrAfUfU-PhfU-PhrArAfU-IfCrGfC | **fCrGfU-PhrArGrA** | rArArArGfCrAfUrGfU-IfCrArArArGfCfCr GrGrArAfCfCrGfUfCfC-PCL-3'

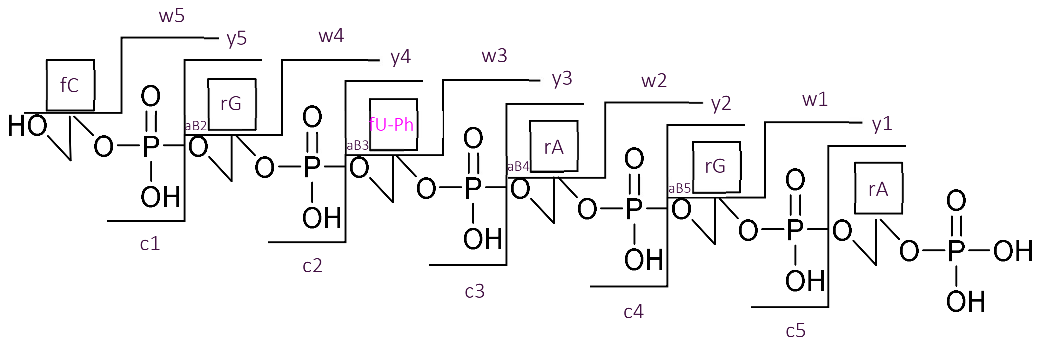

Figure 92. Fragment 4 of **MinE07UB** 5'-3'.

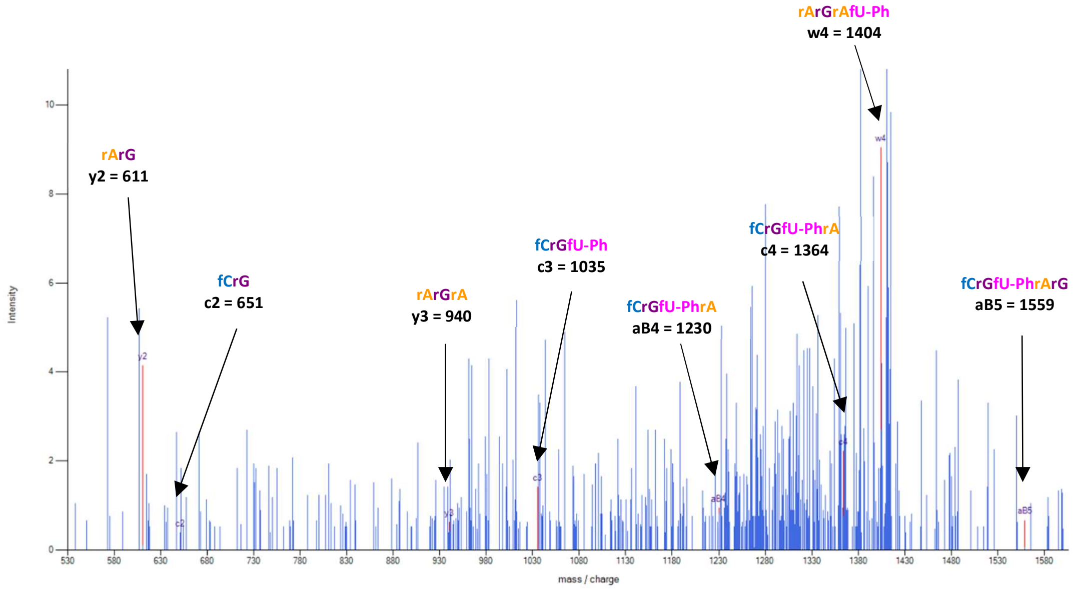

Figure 93. LC-MS/MS Fragment 4 Data of **MinE07UB**.

Fragment 5 of MinE07UB

5'-rGrGrAfCrGrGrAfUfU-PhfU-PhrArAfU-IfCrGfCfCrGfU-PhrA | **rGrArArArA** | rGfCrAfUrGfU-IfCrArArArGfCfCr GrGrArAfCfCrGfUfCfC-PCL-3'

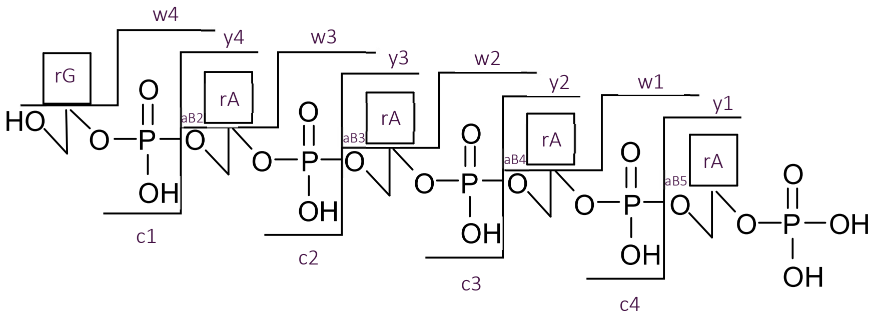

Figure 94. Fragment 5 of **MinE07UB** 5'-3'.

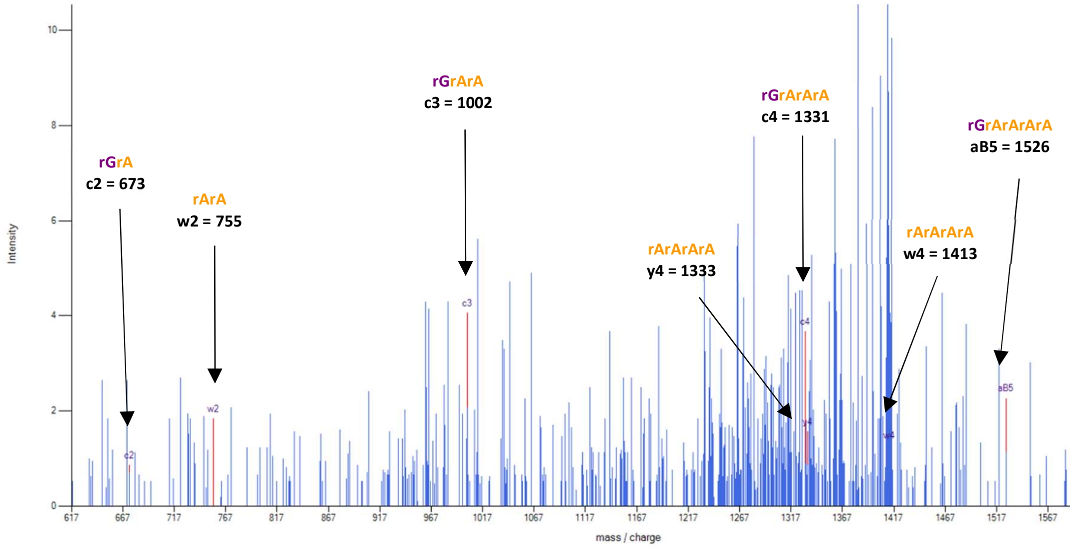

Figure 95. LC-MS/MS Fragment 5 Data of **MinE07UB**.

Fragment 6 of MinE07UB

5'-rGrGrAfCrGrGrAfUfU-PhfU-PhrArAfU-IfCrGfCfCrGfU-PhrArGrArArArArG | **fCrAfUrGfU-IfC |**

rArArArGfCfCrGrGrArAfCfCrGfUfCfC-PCL-3'

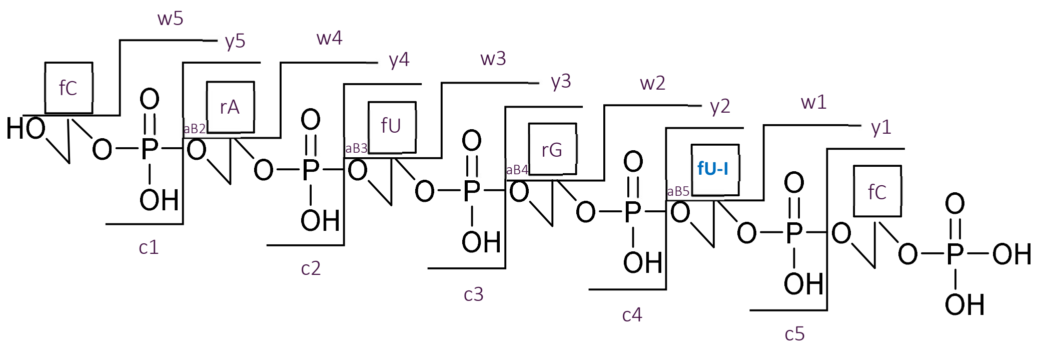

Figure 96. Fragment 6 of **MinE07UB** 5'-3'.

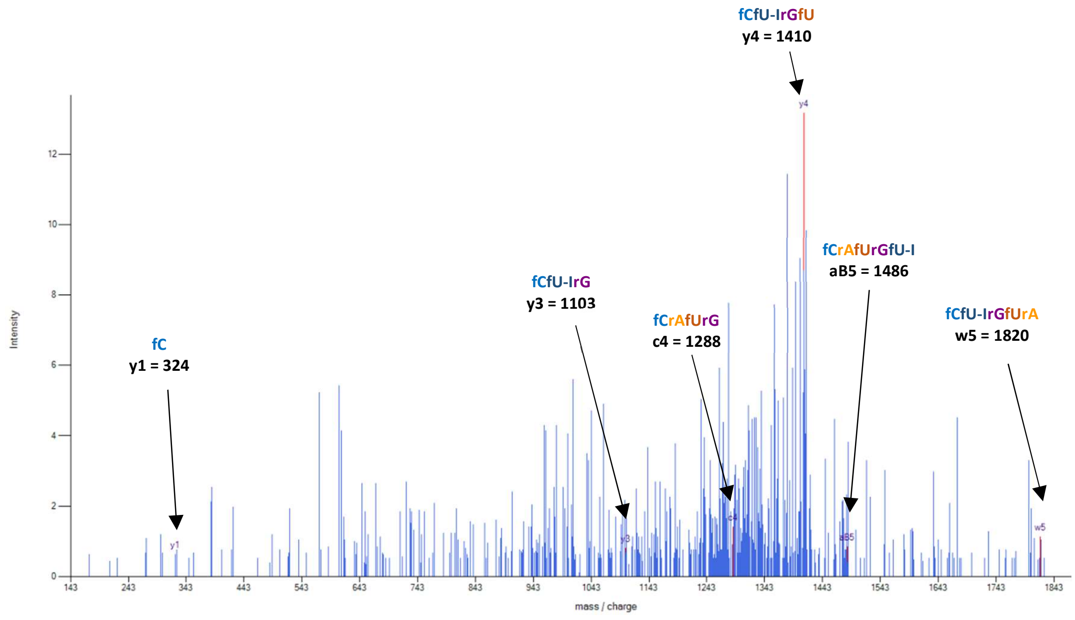

Figure 97. LC-MS/MS Fragment 6 Data of **MinE07UB**.

Fragment 7 of MinE07UB

5'-rGrGrAfCrGrGrAfUfU-PhfU-PhrArAfU-IfCrGfCfCrGfU-PhrArGrArArArArGfC | **rAfUrGfU-IfC** | rArArArGfCfCrGrGrArAfCfCrGfUfCfC-PCL-3'

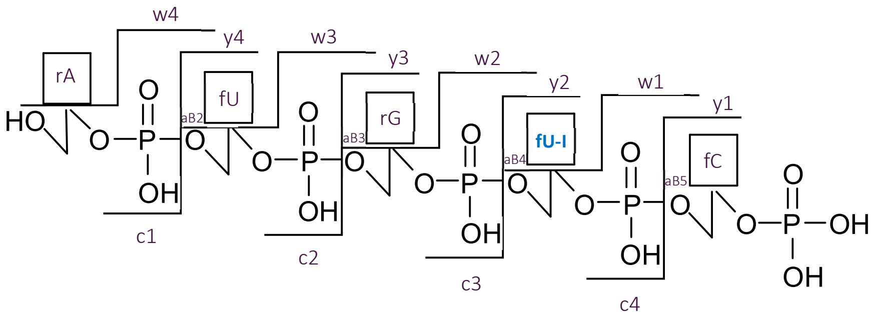

Figure 98. Fragment 7 of **MinE07UB** 5'-3'.

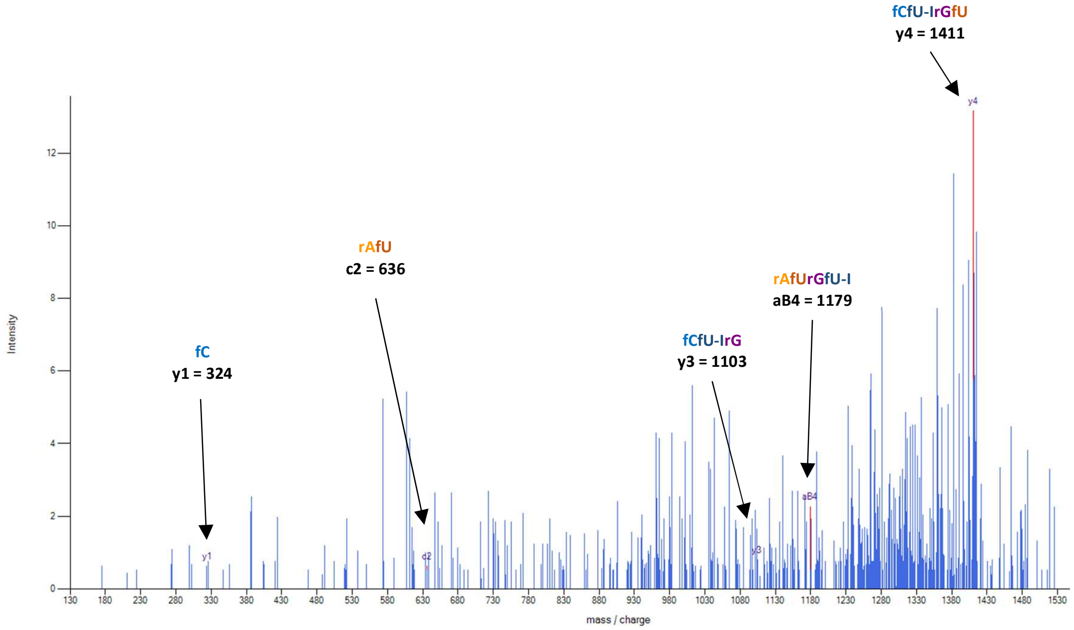

Figure 99. LC-MS/MS Fragment 7 Data of **MinE07UB**.

Fragment 8 of MinE07UB

5'-rGrGrAfCrGrGrAfUfU-PhfU-PhrArAfU-IfCrGfCfCrGfU-PhrArGrArArArArGfCrAfUrGfU-IfCrArArArGfCfCrGrG **| rArAfCfCrGfU |** fCfC-PCL-3'

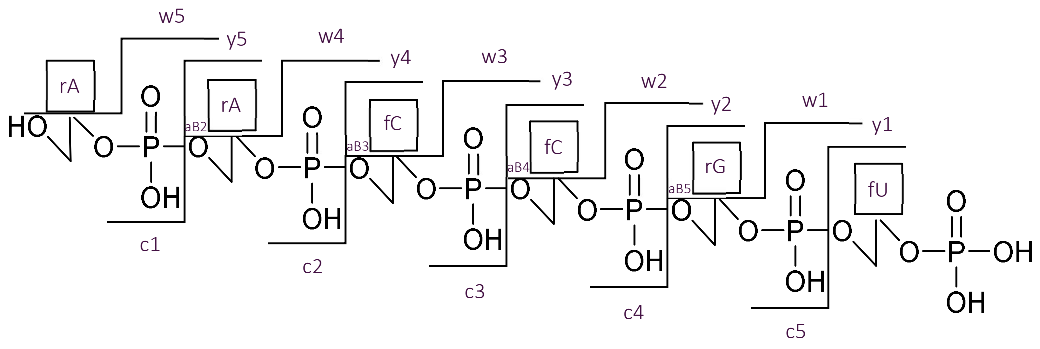

Figure 100. Fragment 8 of **MinE07UB** 5'-3'.

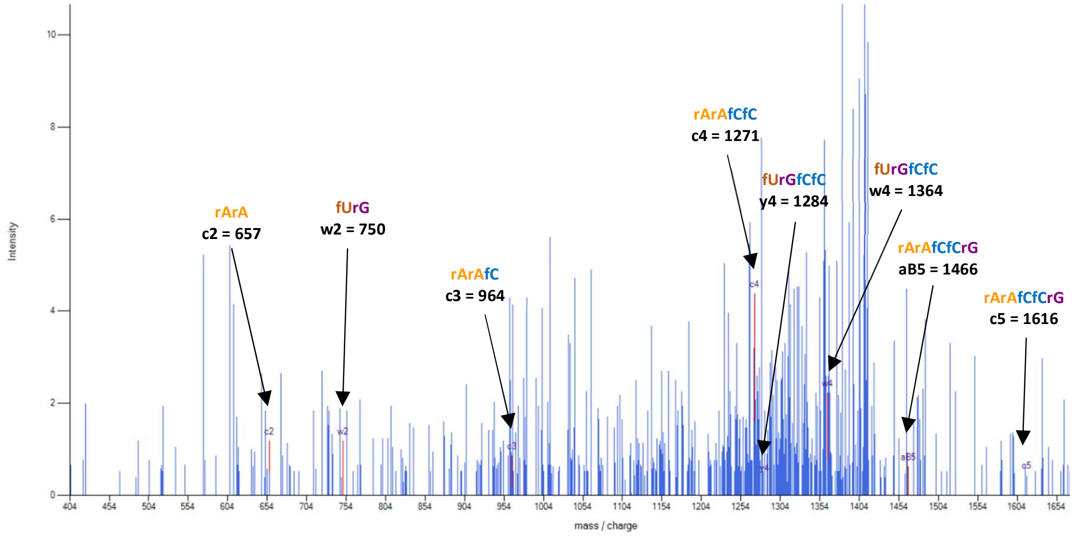

Figure 101. LC-MS/MS Fragment 8 Data of **MinE07UB**.

Fragment 9 of MinE07UB

5'-rGrGrGrAfCrGrGrAfUfU-PhfU-PhrArAfU-IfCrGfCfCrGfU-PhrArGrArArArArGfCrAfUrGfU-IfCrArArArGfCfCrGrGrA | **rAfCfCrGfUfC |** fC-PCL-3'

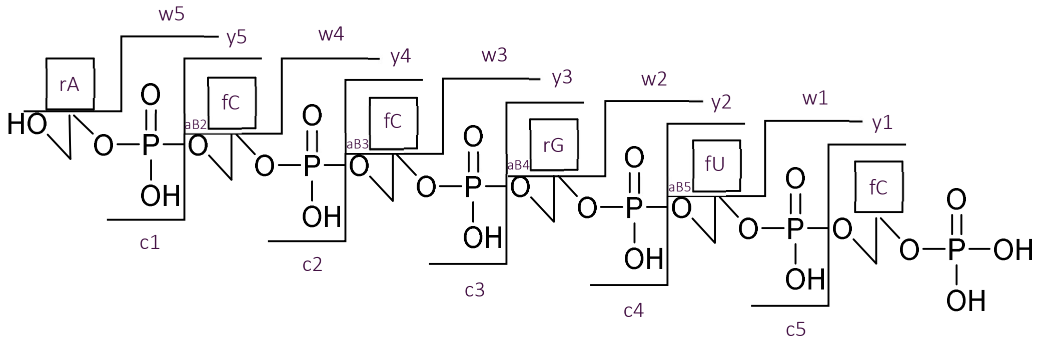

Figure 102. Fragment 9 of **MinE07UB** 5'-3'.

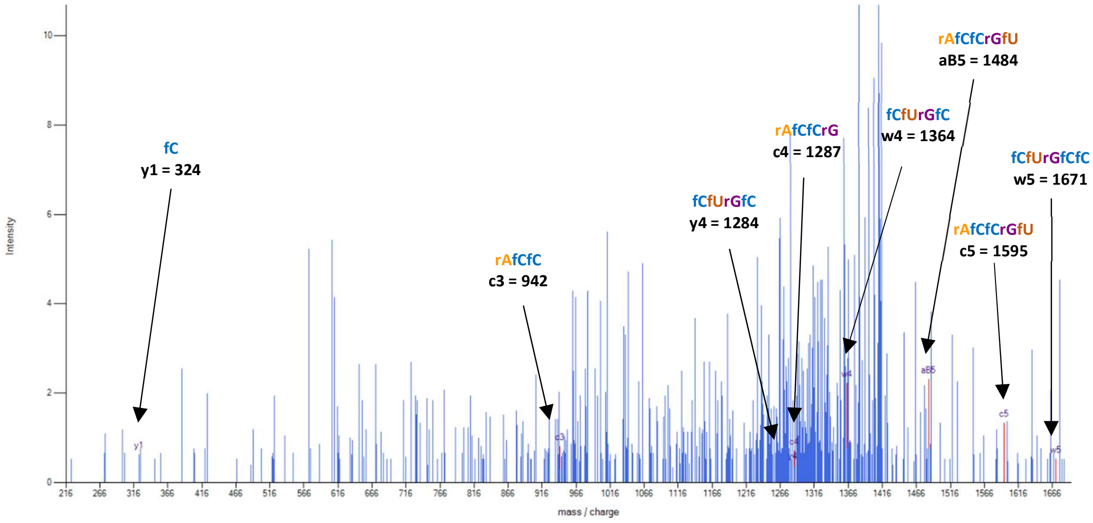

Figure 103. LC-MS/MS Fragment 9 Data of **MinE07UB**.

LC-MS/MS Analysis of MinE07UC

MinE07-139: 5’-rGrGrAfU-VifUfUrArAfUfCrGfCfCrGfUrArGrArArArArGfCrAfU-PhrGfU-VifCrArArArGfCfCrGrG rArAfCfCrGfUfCfC-PCL-3’

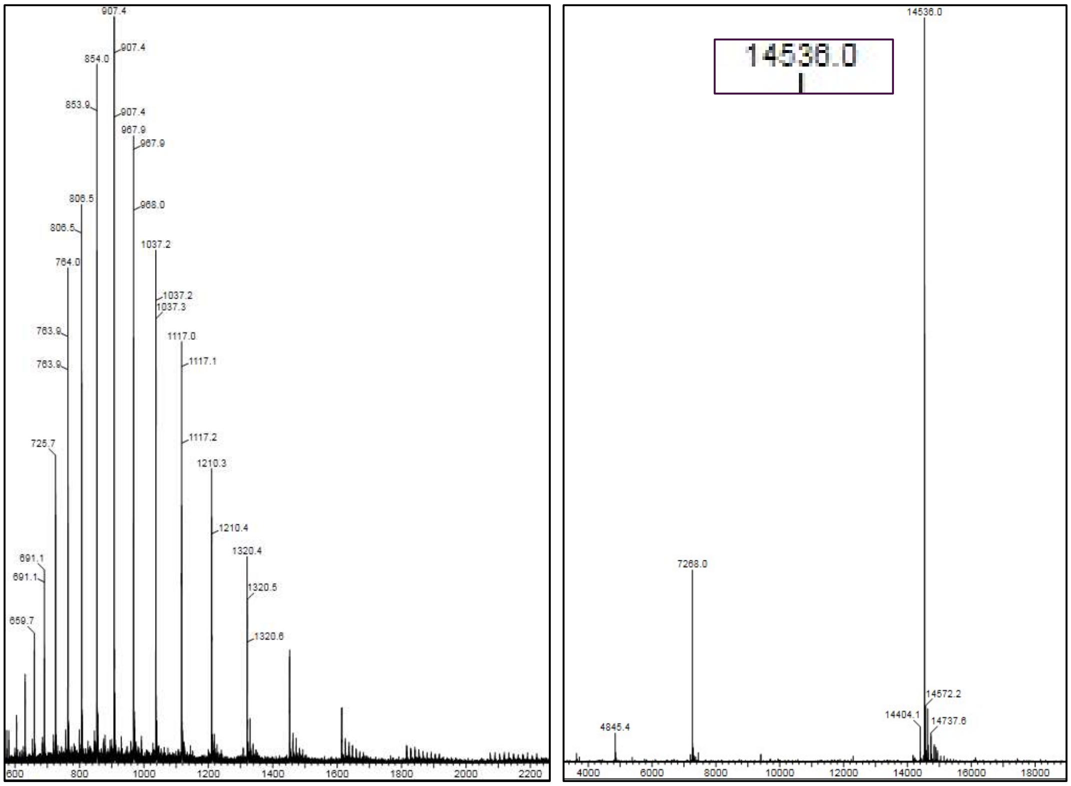

Figure 104. Mass spectrum of MinE07UC and predicted mass

Fragment 1 of MinE07UC

5'-rG | **rGrAfU-VifUfU** | rArAfUfCrGfCfCrGfUrArGrArArArArGfCrAfU-PhrGfU-VifCrArArArGfCfCrGrGrArAfCfC rGfUfCfC-PCL-3'

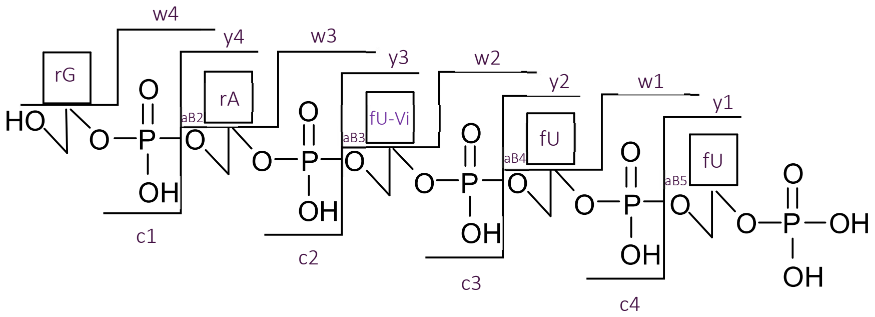

Figure 105. Fragment 1 of **MinE07UC** 5'-3'.

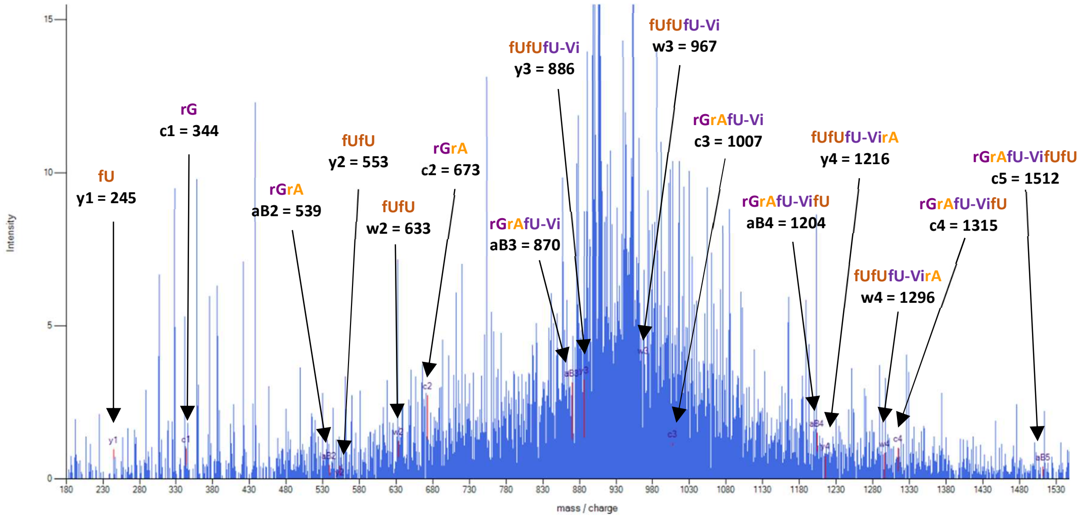

Figure 106. LC-MS/MS Fragment 1 Data of **MinE07UC**.

Fragment 2 of MinE07UC

5'-rGrG **| rAfU-VifUfUfUrArA |** fUfCrGfCfCrGfUrArGrArArArArGfCrAfU-PhrGfU-VifCrArArArGfCfCrGrGrArAfCfC rGfUfCfC-PCL-3'

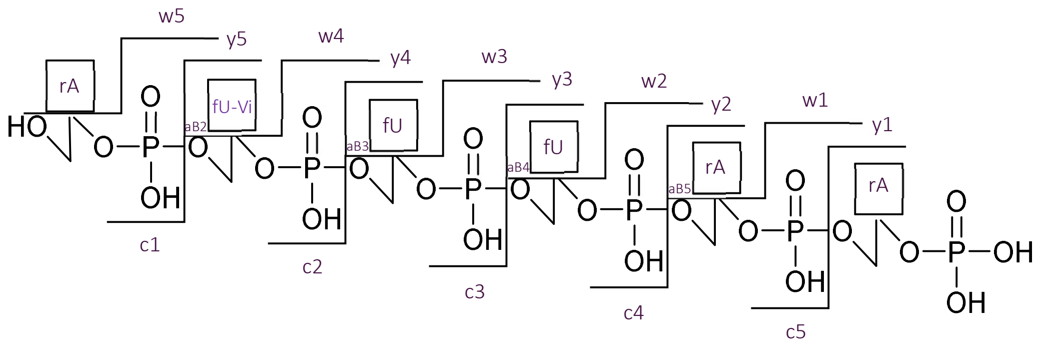

Figure 107. Fragment 2 of **MinE07UC** 5'-3'.

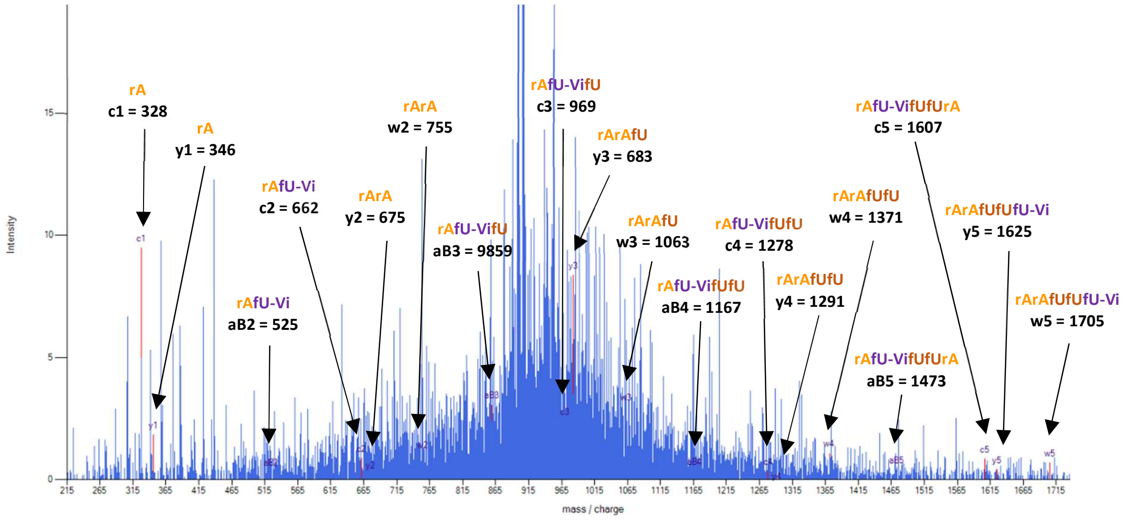

Figure 108. LC-MS/MS Fragment 2 Data of **MinE07UC**.

Fragment 3 of MinE07UC

5'-rGrGrAfU-VifUfU | **rArAfUfCrGfC** | fCrGfUrArGrArArArArGfCrAfU-PhrGfU-VifCrArArArGfCfCrGrGrArAfCfC rGfUfCfC-PCL-3'

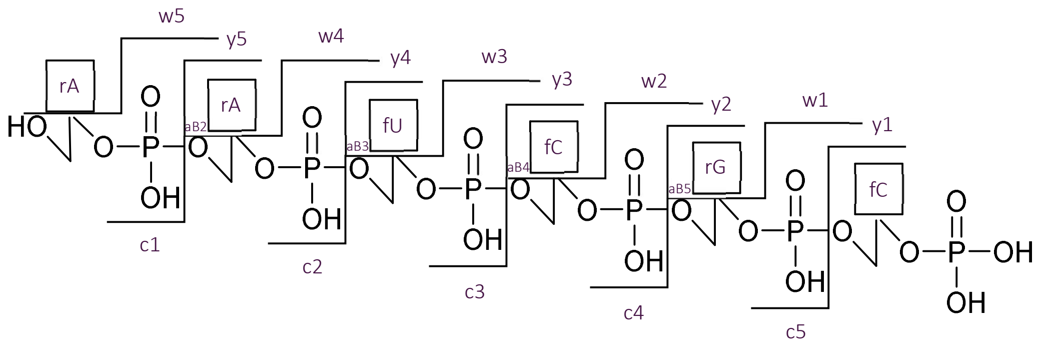

Figure 109. Fragment 3 of **MinE07UC** 5'-3'.

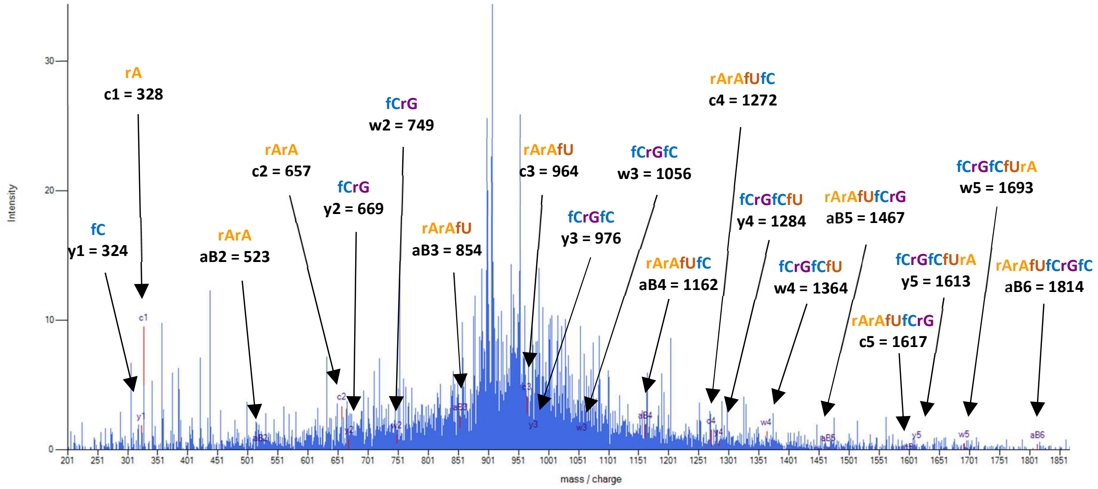

Figure 110. LC-MS/MS Fragment 3 Data of **MinE07UC**.

Fragment 4 of MinE07UC

5'-rGrGrAfU-VifUfUrA | **rAfUfCrGfC** | fCrGfUrArGrArArArArGfCrAfU-PhrGfU-VifCrArArArGfCfCrGrGrArAfCfC rGfUfCfC-PCL-3'

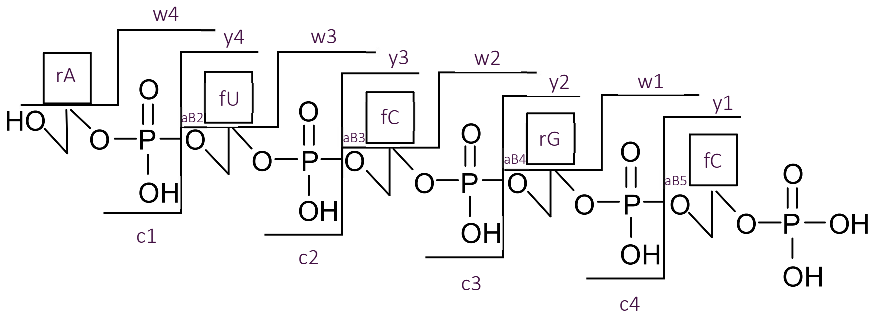

Figure 111. Fragment 4 of **MinE07UC** 5'-3'.

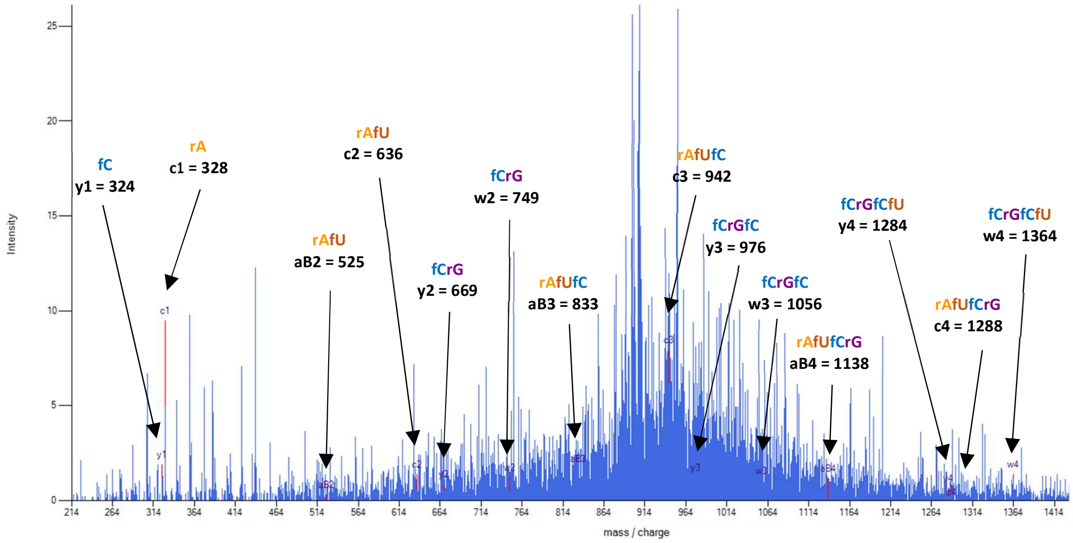

Figure 112. LC-MS/MS Fragment 4 Data of **MinE07UC**.

Fragment 5 of MinE07UC

5'-rGrGrAfU-VifUfUfUrArAfUfC | **rGfCfCrGfUrA** | rGrArArArArGfCrAfU-PhrGfU-VifCrArArArGfCfCrGrGrArAfCfC rGfUfCfC-PCL-3'

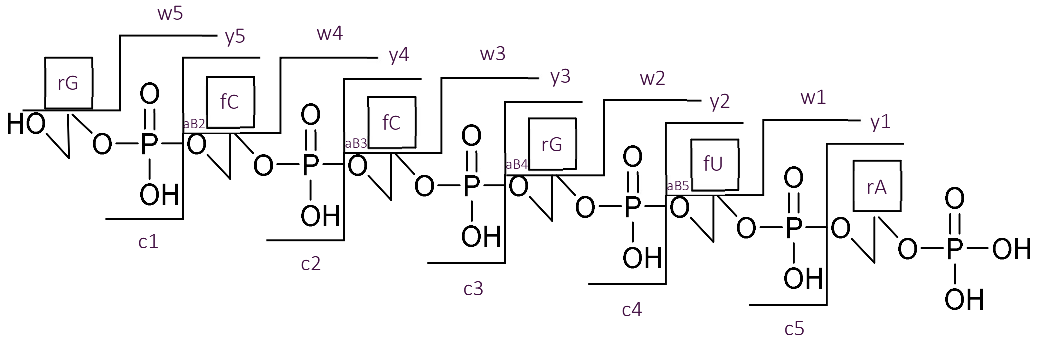

Figure 113. Fragment 5 of **MinE07UC** 5'-3'.

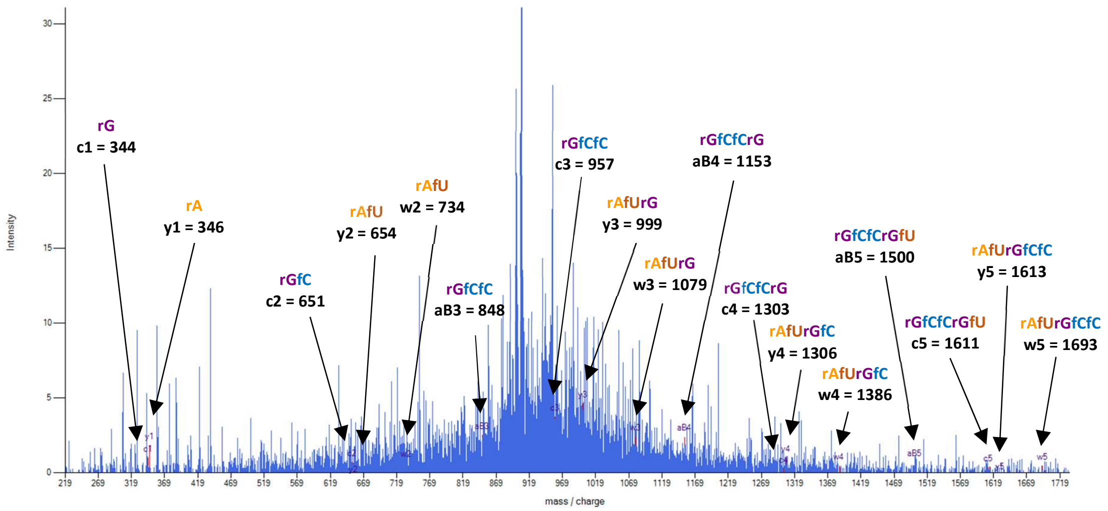

Figure 114. LC-MS/MS Fragment 5 Data of **MinE07UC**.

Fragment 6 of MinE07UC

5'-rGrGrAfU-VifUfUrArAfUfCrGfCfCrGfU | **rArGrArArArA** | rGfCrAfU-PhrGfU-VifCrArArArGfCfCrGrGrArAfCfC rGfUfCfC-PCL-3'

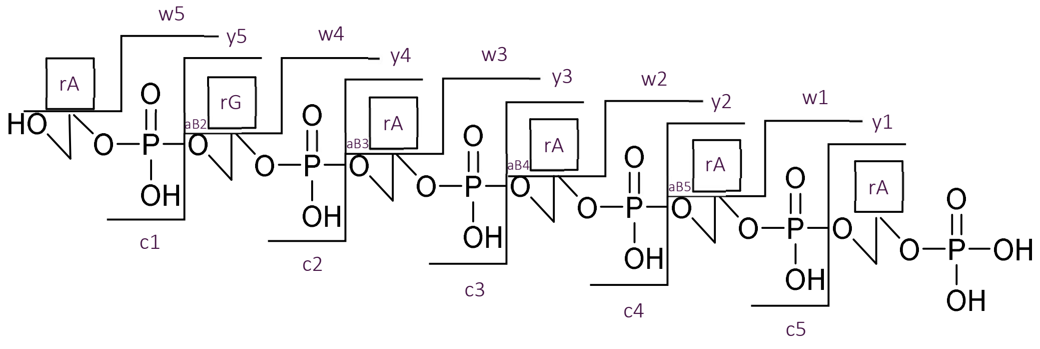

Figure 115. Fragment 6 of **MinE07UC** 5'-3'.

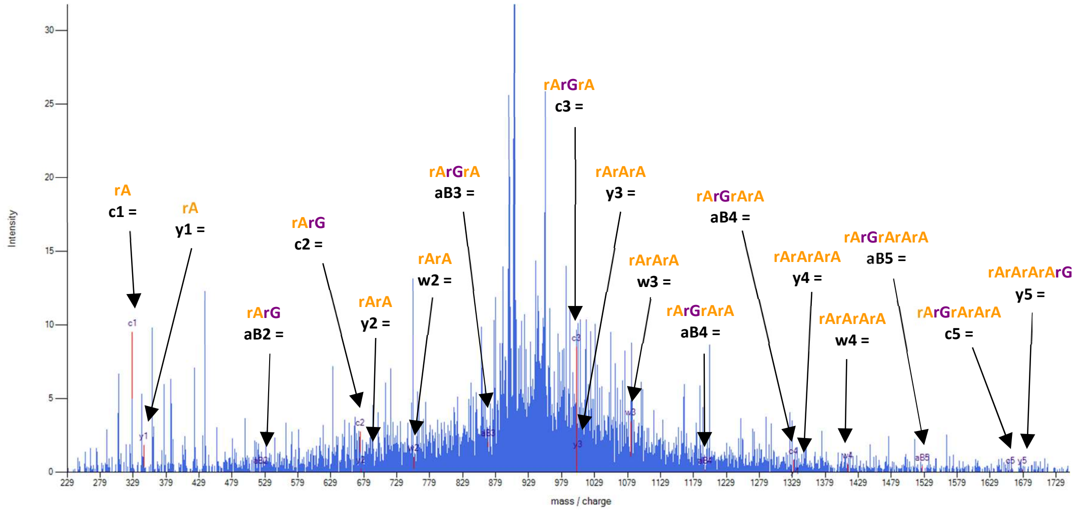

Figure 116. LC-MS/MS Fragment 6 Data of **MinE07UC**.

Fragment 7 of MinE07UC

5'-rGrGrAfU-VifUfUrArAfUfCrGfCfCrGfUrArGrArArArArGfC | **rAfU-PhrGfU-VifCrA** | rArArGfCfCrGrGrArAfCfC rGfUfCfC-PCL-3'

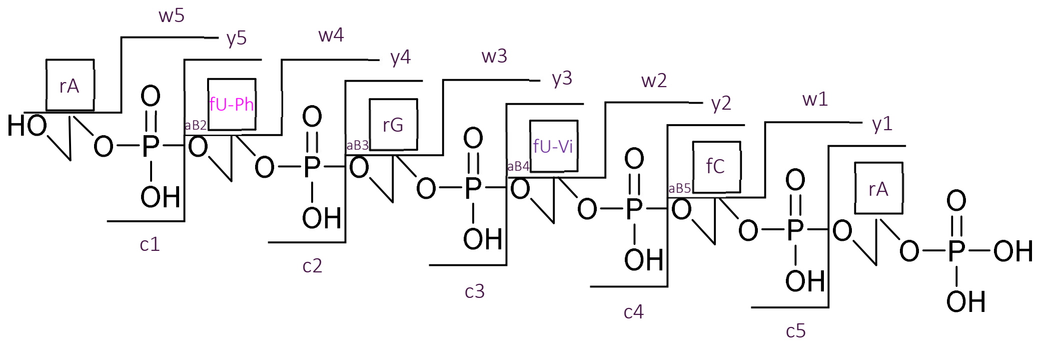

Figure 117. Fragment 7 of **MinE07UC** 5'-3'.

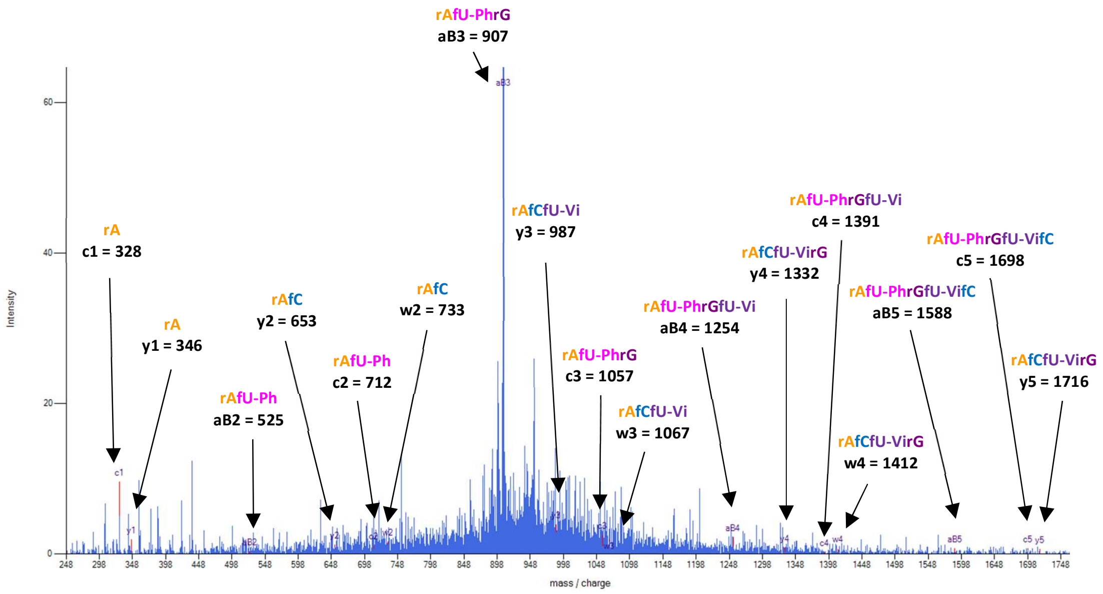

Figure 118. LC-MS/MS Fragment 7 Data of **MinE07UC**.

Fragment 8 of MinE07UC

5'-rGrGrAfU-VifUfUrArAfUfCrGfCfCrGfUrArGrArArArArGfCrAfU-PhrGfU-Vi | **fCrArArArG** | fCfCrGrGrArAfCfC rGfUfCfC-PCL-3'

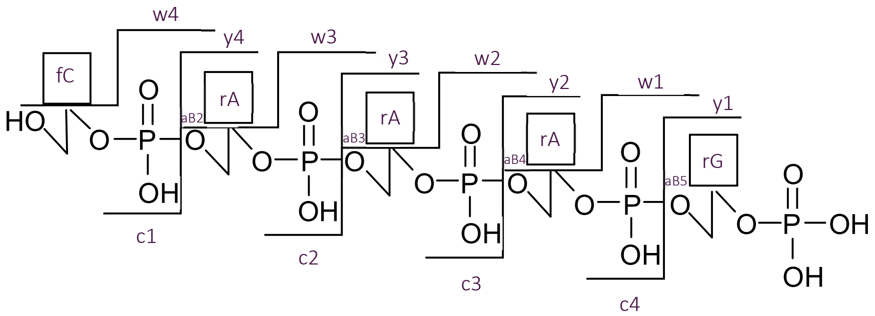

Figure 119. Fragment 8 of **MinE07UC** 5'-3'.

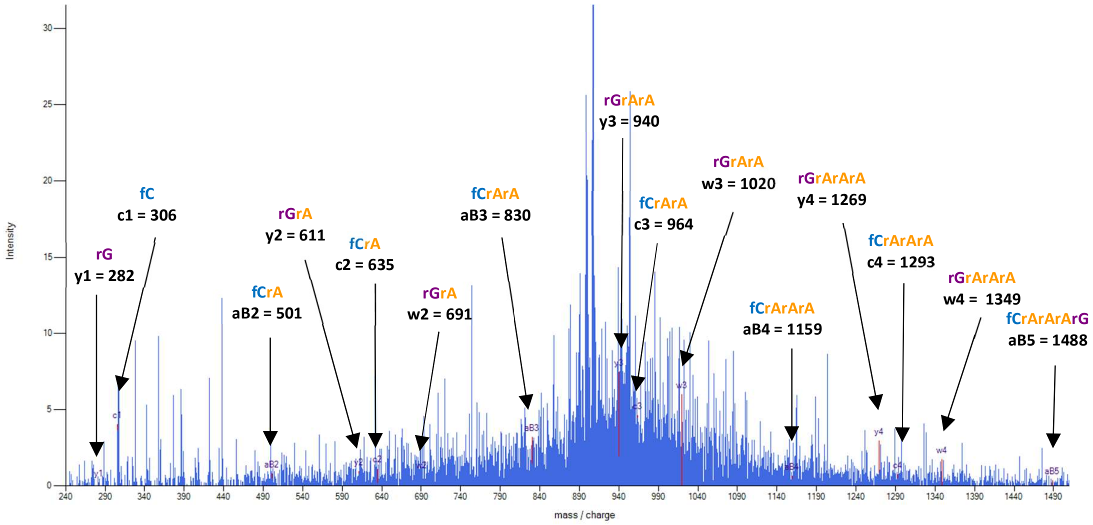

Figure 120. LC-MS/MS Fragment 8 Data of **MinE07UC**.

Fragment 9 of MinE07UC

5'-rGrGrAfU-VifUfUrArAfUfCrGfCfCrGfUrArGrArArArArGfCrAfU-PhrGfU-VifCrArArArG **| fCfCrGrGrArA |** fCfC rGfUfCfC-PCL-3'

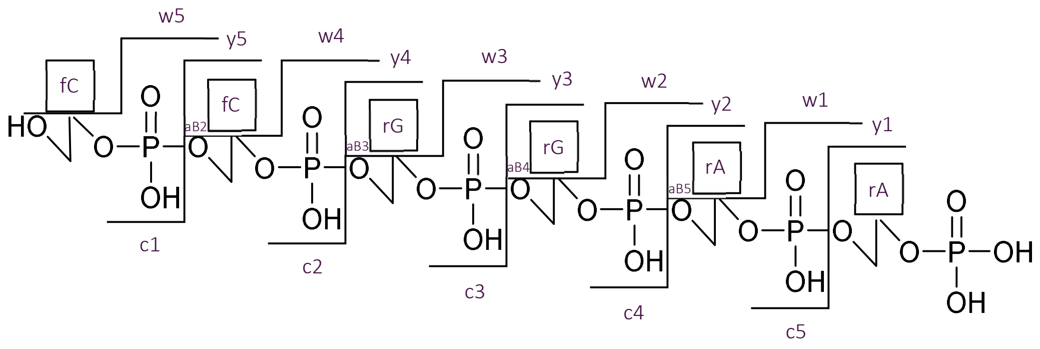

Figure 121. Fragment 9 of **MinE07UC** 5'-3'.

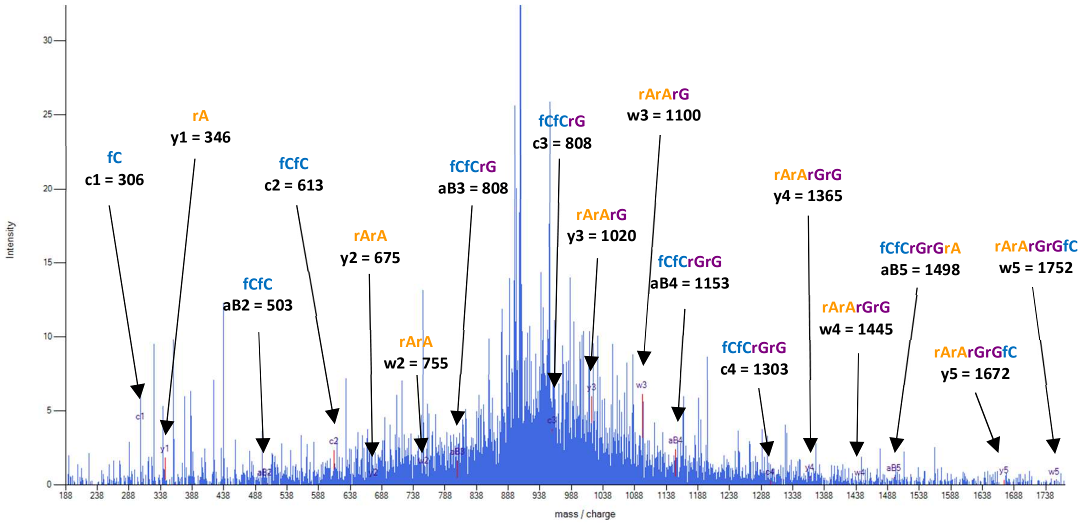

Figure 122. LC-MS/MS Fragment 9 Data of **MinE07UC**.

Fragment 10 of MinE07UC

5'-rGrGrAfU-VifUfUrArAfUfCrGfCfCrGfUrArGrArArArArGfCrAfU-PhrGfU-VifCrArArArGfCfCrG **| rGrArAfCfC |** rGfUfCfC-PCL-3'

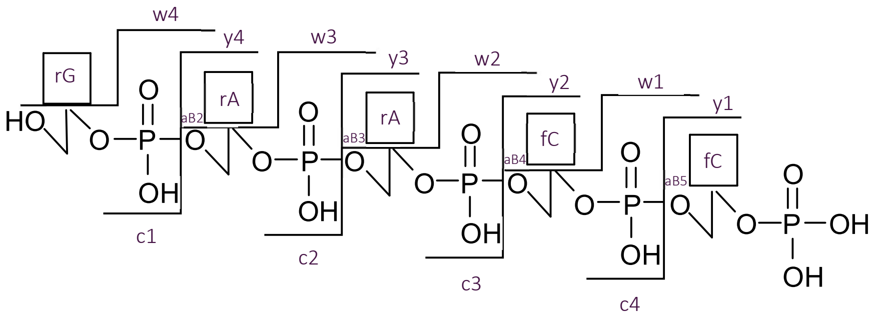

Figure 123. Fragment 10 of **MinE07UC** 5'-3'.

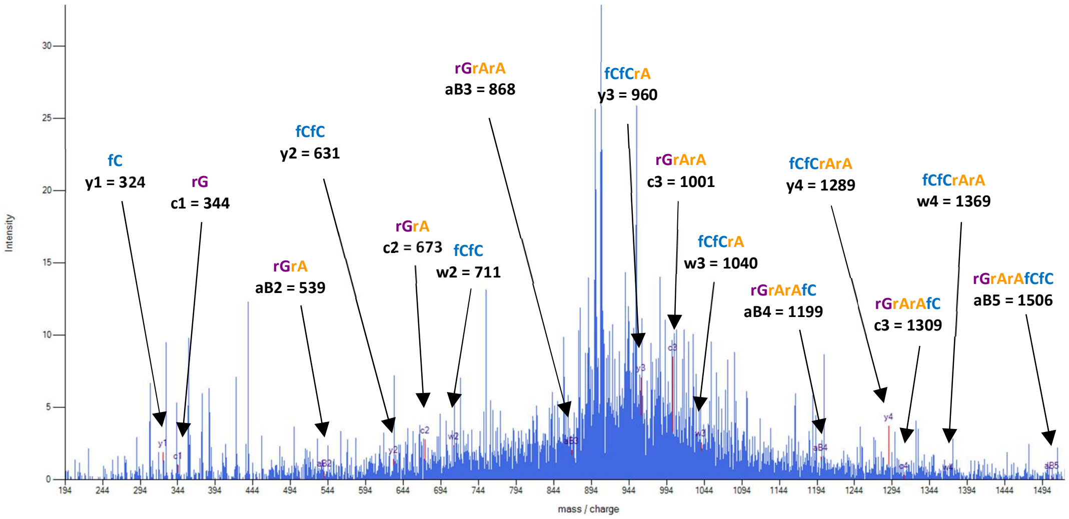

Figure 124. LC-MS/MS Fragment 10 Data of **MinE07UC**.

Fragment 11 of MinE07UC

5'-rGrGrAfU-VifUfUfUrArAfUfCrGfCfCrGfUrArGrArArArArGfCrAfU-PhrGfU-VifCrArArArGfCfCrGrGrArAfC **| fCrG fUfCfC-PCL |** -3'

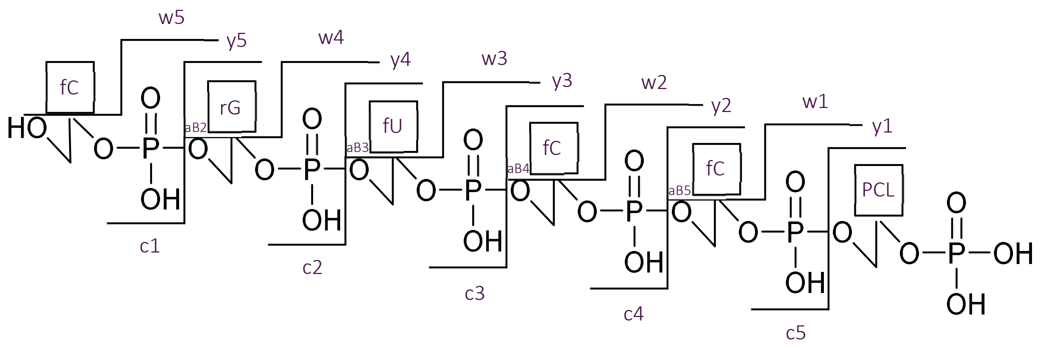

Figure 125. Fragment 11 of **MinE07UC** 5'-3'.

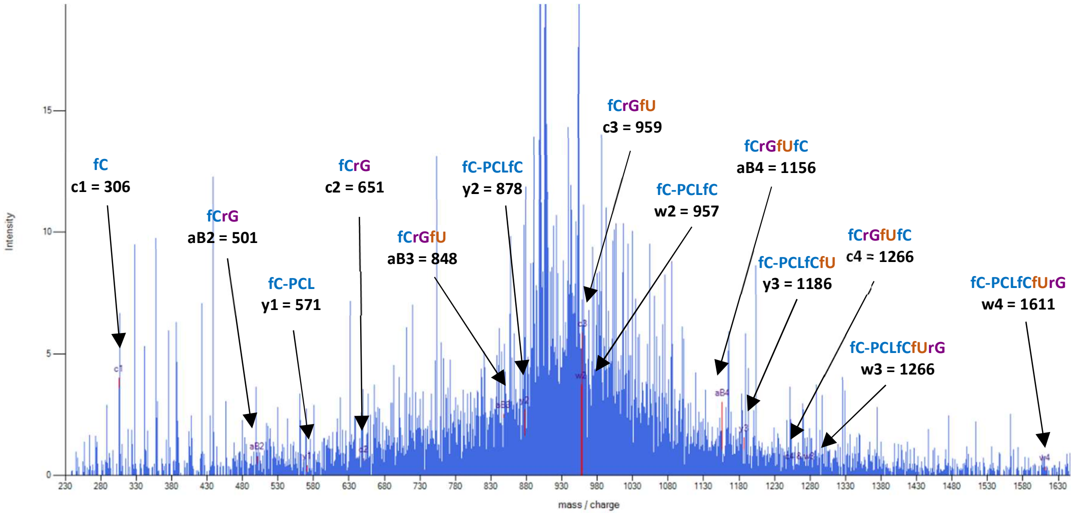

Figure 126. LC-MS/MS Fragment 11 Data of **MinE07UC**.

S7. Aptamer Resynthesis

Synthesis of modified aptamers: MinE07UA-Biotin, MinE07UA'-Biotin, MinE07UB-Biotin, MinE07UC-Biotin and MinE07UC'-Biotin. Universal UnyLinker support (0.021 g) was added to a synthesiser column. See experimental method 4.3.1 for the setup of the synthesiser. The sequences were uploaded on to the synthesiser:

MinE07UA-Biotin:5'-Biotin-fCrGrGrAfUfU-PhfU-PhrArAfUfCrGfCfCrGfU-IrArGrArArArArGfCrAfUrGfUfCrArAr ArGfCfCrGrGrArAfCfCrGfU-PhfCfC-3'. This was run to completion (28.9% yield). MinE07UA'-Biotin:5'-Biotin-rGrGrAfCrGrGrAfUfU-PhfU-PhrArAfUfCrGfCfCrGfU-IrArGrArArArArGfCrAfUrGfUf CrArArArGfCfCrGrGrArAfCfCrGfU-PhfCfC-3'. This was run to completion (15.8% yield). MinE07UB-Biotin:5'-Biotin-rGrGrAfCrGrGrAfUfU-PhfU-PhrArAfU-IfCrGfCfCrGfU-PhrArGrArArArArGfCrAfUrG fUfCrArArArGfCfCrGrGrArAfCfCrGfUfCfC-3'. This was run to completion (21.4% yield). MinE07UC-Biotin:5'-Biotin-rGrGrAfU-VifUfUrArAfUfCrGfCfCrGfUrArGrArArArArGfCrAfU-PhrGfU-VifCrArArAr GfCfCrGrGrArAfCfCrGfUfCfC-3'. This was run to completion (18.4% yield). MinE07UC'-Biotin:5'-Biotin-rGrGrAfCrGrGrAfU-VifUfUrArAfUfCrGfCfCrGfUrArGrArArArArGfCrAfU-PhrGfU-Vi fCrArArArGfCfCrGrGrArAfCfCrGfUfCfC-3'. This was run to completion (21.4% yield). These aptamers were cleavage from their solid support, desalted, PAGE purified and ethanol precipitated as before.

Cleavage of MinE07-Biotin, MinE07-U-Ph-Biotin, MinE07-U-Vi-Biotin and MinE07-U-I-Biotin Aptamers from Universal UnyLinker Support. MinE07-Biotin, MinE07M3-Biotin, MinE07M6-Biotin, MinE07-U-Ph-Biotin, MinE07-U-Vi-Biotin and MinE07-U-I-Biotin aptamers were cleaved and deprotected. The solid support resin was removed from the column and placed into a screw-cap centrifuge tube and to this 1.5 mL of ammonia was added. The aptamers were incubated at 55 °C overnight in a stirring water bath. The aptamers are then placed in a centrifugal vacuum concentrator to remove the ammonia solution. The aptamers were then each re-suspended in autoclaved water.

Desalt of MinE07-Biotin, MinE07-U-Ph-Biotin, MinE07-U-Vi-Biotin and MinE07-U-I-Biotin Aptamers using Zetadex. Purification of the RNA aptamers was carried out by size exclusion gel chromatography using Zetadex resin (emp Biotech). A 20 mL column was prepared with 1 mL of cotton wool at the bottom of the column. A slurry of zetadex resin and autoclaved water was prepared for use in the column. 1 mL of aptamer was loaded onto the column and the column was flushed with deionised water. 1 mL fractions were collected. This was repeated for each aptamer, with a new column each time. The fractions were run on the Nanodrop UV-Vis spectrophotometer. The fractions containing an RNA signal were pooled. dried down and re-suspended in reverse-osmosis purified water and stored in the freezer (-20°C).

PAGE-based analysis of Aptamers. TBE denaturing polyacrylamide gels were prepared from a denaturing 20% polyacrylamide stock solution and TBE buffer, with the final concentrations of polyacrylamide at 15%. The gels were polymerised by addition of 5 µL TEMED (1 µL/mL of gel), followed by APS 5 µL (40% stock concentration). The solution was mixed thoroughly and poured between glass plates with 0.75 mm spacers, followed by insertion of a 0.75 mm comb. The gel was then left to fully polymerise. Approximately 80% of the cassette was filled with TBE buffer. The comb was removed after polymerisation and the wells were flushed with deionised water, and then buffer. 10 µL at 20 nM of each aptamer sample in water was prepared along with 10 µL urea (8 M). 20 µL of each sample was loaded onto the polyacrylamide gel. All gels were run at 300 V, 15 mA, for 60 minutes. Gels were stained in Stains-All prepared in isopropanol-tris buffer, for over an hour. The gels were then rinsed to remove excess stain using water before being imaged on an Epsom scanner.

PAGE-based Purification of Aptamers. MinE07-Biotin, MinE07-U-Ph-Biotin, MinE07-U-Vi-Biotin and MinE07-U-I-Biotin aptamers required purification by Poly Acrylamide Gel Electrophoresis (PAGE). A 1.5 mm 15% denaturing PAGE gel was produced by diluting 37.5 mL of 20% acrylamide denaturing stock solution with 12.5 mL of 1 x TBE buffer. To this 50 µL of TEMED and 130 µL of 40% APS stock solution were added to induce polymerisation. The gel was poured between two glass plates and a 1.5 mm comb was inserted. The comb

was removed after casting and the well washed with TBE buffer. The gel was pre-run at 300 V for 1 hour before the sample was loaded. The aptamers with 8 M urea were loaded onto the gel and run at 250 V for half an hour before being run for a further 1.5 hours at 300 V. The gel was removed from the glass plates and placed onto cling film, which was then placed onto a silica TLC plate; the gel was illuminated with UV light to visualise each aptamer. The aptamer band was cut out of the bulk gel and placed into a 15 mL falcon tube. The gel was homogenised, and 10 mL of autoclaved water was added. The solution was mixed and rapidly frozen in liquid nitrogen before being incubated overnight at 60 °C in a water bath. The solution was split into two equal portions and centrifuged for five minutes, the supernatants were collected, and the pellet extracted using a pipette with 2 mL autoclaved water. This was repeated three times. The supernatants were dried by centrifugal vacuum concentration at 60 °C for five hours and the pellets were re-suspended in autoclaved water.

Ethanol Precipitation of Aptamers after PAGE Purification. Aptamers were desalted by ethanol precipitation; the samples were incubated with 3 M sodium acetate and 100% ethanol over night before being centrifuged for 20 minutes. The supernatant was removed, and the pellet washed with 1 mL of ethanol and centrifuged again; this was repeated three times for each sample. The samples were then left to air dry before being re-suspended in autoclaved water.

Determination of Aptamer concentration by UV-Visible spectrophotometry. Aptamer concentrations were analysed by UV-Visible spectrometry using a Nanodrop spectrophotometer.UV-visible absorption spectra were recorded on a NanoDrop One UV-Vis spectrophotometer by Thermo Fisher Scientific. 2 µL of deionised water was placed onto the stage and this blank was run through the machine. The stage was cleaned with deionised water and a Kimtech wipe. 2 µL of sample was then placed onto the stage and a spectrum was run between the regions of 200 – 360 nm. Each sample was repeated until a minimum of three concordant results were achieved. Using the calculated A_{260} from Intregrated DNA Technologies (IDT) (2.11) and the measured A_{260} from the UV-Vis data, a concentration was calculated by multiplying them together.

MinE07-Biotin: 5'-Biotin-rGrGrAfCrGrGrAfUfUfUrArAfUfCrGfCfCrGfUrArGrArArArArGfCrAfUrGfUfCrArArArG fCfCrGrGrArAfCfCrGfUfCfC-3'

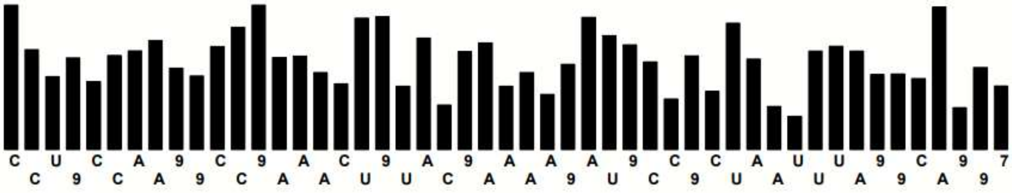

Figure 127. Trityl Monitor of the Synthesis of **Biotin-MinE07** on Universal UnyLinker Solid Support. Guanine is labelled as 9 and the biotin is labelled as 7.

Sequence {5'-3'}

799 AC9 9AU UUA AUC 9CC 9UA 9AA AA9 CAU 9UC AAA 9CC 99A ACC 9UC C

Trityl Histogram for synthesis: AlixRNAaptamerBIOTIN9 49 mer.
Efficiency(Stepwise Yield by base leveling): 97.3%
Final Yield (by rolling average): 100.0%
Stepwise Yield for Monomer: C: 86.3 %
Stepwise Yield for Monomer: U: 100.0 %
Stepwise Yield for Monomer: 9: 99.9 %
Stepwise Yield for Monomer: A: 100.0 %
Stepwise Yield for Monomer: 7: 100.0 %

Figure 128. Aptamer Synthesis Report of the Synthesis of **MinE07-Biotin** on Universal UnyLinker Solid Support. The step-wise coupling efficiency was 97.3%. The overall average yield: $\boldsymbol{0.973^{49} = 0.261}$. This gives a 26.1% overall synthetic yield.

MinE07UA-Biotin: 5'-Biotin-fCrGrGrAfUfU-PhfU-PhrArAfUfCrGfCfCrGfU-IrArGrArArArArGfCrAfUrGfUfCrArAr ArGfCfCrGrGrArAfCfCrGfU-PhfCfC-3'.

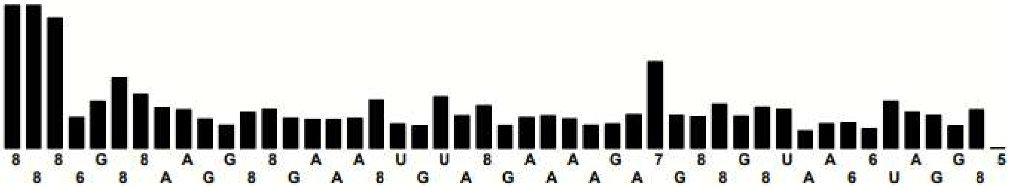

Figure 129. Trityl Monitor of the Synthesis of **MinE07UA-Biotin** on Universal UnyLinker Solid Support Cytosine is labelled as 8, fU-Ph is labelled at 6, fU-I is labelled as 7 and the biotin is labelled as 5. The trityl monitor gave a coupling efficiently of 1.46e[6].

Sequence {5'-3'}

58G GAU 66A AU8 G88 G7A GAA AAG 8AU GU8 AAA G88 GGA A88 G68
88

Trityl Histogram for synthesis: ARP-BIOMINE07-41 47 mer.
Efficiency(Stepwise Yield by base leveling): 97.5%
Final Yield (by rolling average): 100.0%
Stepwise Yield for Monomer: 8: 88.8 %
Stepwise Yield for Monomer: 6: 99.4 %
Stepwise Yield for Monomer: G: 96.3 %
Stepwise Yield for Monomer: A: 97.8 %
Stepwise Yield for Monomer: U: 100.0 %
Stepwise Yield for Monomer: 7: 100.0 %
Stepwise Yield for Monomer: 5: 100.0 %

Figure 130. Aptamer Synthesis Report of the Synthesis of **MinE07-41-Biotin** on Universal UnyLinker Solid Support. The step wise coupling efficiency is 97.5%. The overall average yield: $\boldsymbol{0.975^{49} = 0.289}$. This gives a 28.9% overall synthetic yield.

MinE07A'-Biotin: 5'-Biotin-rGrGrAfCrGrGrAfUfU-PhfU-PhrArAfUfCrGfCfCrGfU-IrArGrArArArArGfCrAfUrGfUf CrArArArGfCfCrGrGrArAfCfCrGfU-PhfCfC-3'.

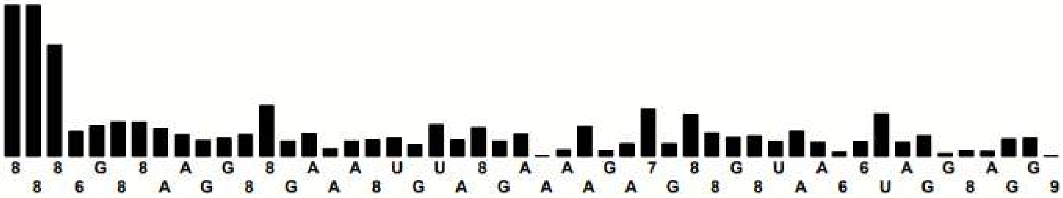

Figure 131. Trityl Monitor of the Synthesis of **MinE07A'-Biotin** on Universal UnyLinker Solid Support Cytosine is labelled as 8, fU-Ph is labelled at 6, fU-I is labelled as 7 and the biotin is labelled as 9. The trityl monitor gave a coupling efficiently of 1.44e[6].

Sequence {5'-3'}

9GG A8G GAU 66A AU8 G88 G7A GAA AAG 8AU GU8 AAA G88 GGA A88
G68 88

Trityl Histogram for synthesis: ARP-BIOMINE07-412 50 mer.
Efficiency(Stepwise Yield by base leveling): 96.3%
Final Yield (by rolling average): 100.0%
Stepwise Yield for Monomer: 8: 89.2 %
Stepwise Yield for Monomer: 6: 97.8 %
Stepwise Yield for Monomer: G: 95.4 %
Stepwise Yield for Monomer: A: 91.9 %
Stepwise Yield for Monomer: U: 100.0 %
Stepwise Yield for Monomer: 7: 100.0 %
Stepwise Yield for Monomer: 9: 100.0 %

Figure 132. Aptamer Synthesis Report of the Synthesis of **MinE07A'-Biotin** on Universal UnyLinker Solid Support. The step wise coupling efficiency is 96.3%. The overall average yield: $\boldsymbol{0.963^{49} = 0.158.}$ This gives a 15.8% overall synthetic yield.

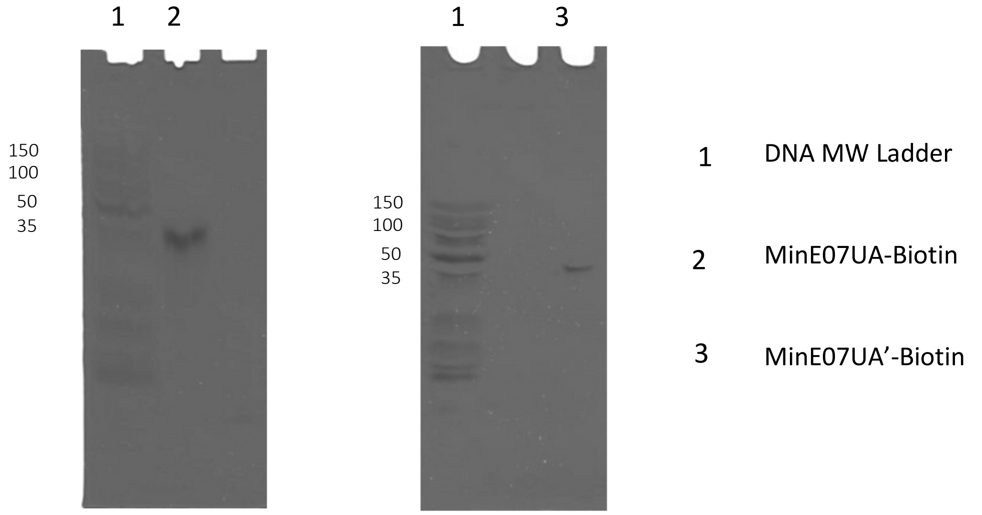

Figure 133. 15% Polyacrylamide Gel of MinE07UA-Biotin and MinE07UA'-Biotin after PAGE purification.

MinE07UB-Biotin: 5'-Biotin-rGrGrArAfCrGrGrAfUfU-PhfU-PhrArAfU-IfCrGfCfCrGfU-PhrArGrArArArArGfCrAfUrGfUfCrArArArGfCfCrGrGrArAfCfCrGfUfCfC-3'

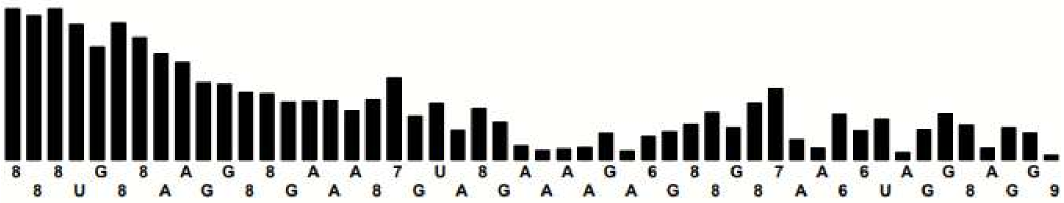

Figure 134. Trityl Monitor of the Synthesis of **MinE07UB-Biotin** on Universal UnyLinker Solid Support Cytosine is labelled as 8, fU-Ph is labelled at 6, fU-I is labelled as 7 and the biotin is labelled as 9. The trityl monitor gave a coupling efficiently of $1.44e^{6}$.

Sequence {5'-3'}

9GG A8G GAU 66A A78 G88 G6A GAA AAG 8AU G78 AAA G88 GGA A88
GU8 88

Trityl Histogram for synthesis: ARP-BIOMINE07-56 50 mer.
Efficiency(Stepwise Yield by base leveling): 96.9%
Final Yield (by rolling average): 100.0%
Stepwise Yield for Monomer: 8: 94.3 %
Stepwise Yield for Monomer: U: 97.3 %
Stepwise Yield for Monomer: G: 95.4 %
Stepwise Yield for Monomer: A: 92.0 %
Stepwise Yield for Monomer: 7: 99.6 %
Stepwise Yield for Monomer: 6: 100.0 %
Stepwise Yield for Monomer: 9: 100.0 %

Figure 135. Aptamer Synthesis Report of the Synthesis of MinE07UB-Biotin on Universal UnyLinker Solid Support. The step wise coupling efficiency is 96.3%. The overall average yield: $\boldsymbol{0.969^{49} = 0.214}$. This gives a 21.4% overall synthetic yield.

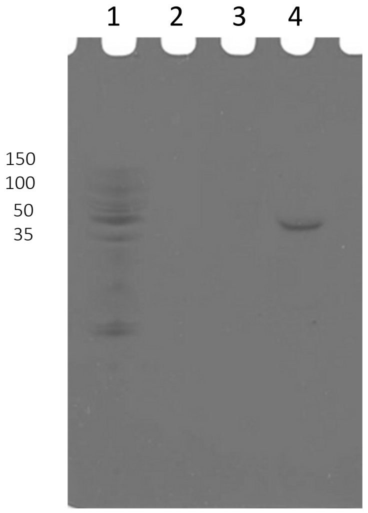

1 DNA MW Ladder

4 MinE07UB-Biotin

Figure 136. 15% Polyacrylamide Gel of **MinE07UB-Bioti**n after PAGE purification.

MinE07UC-Biotin: 5'-Biotin-rGrGrAfU-VifUfUrArAfUfCrGfCfCrGfUrArGrArArArArGfCrAfU-PhrGfU-VifCrArArArGfCfCrGrGrArAfCfCrGfUfCfC-3'

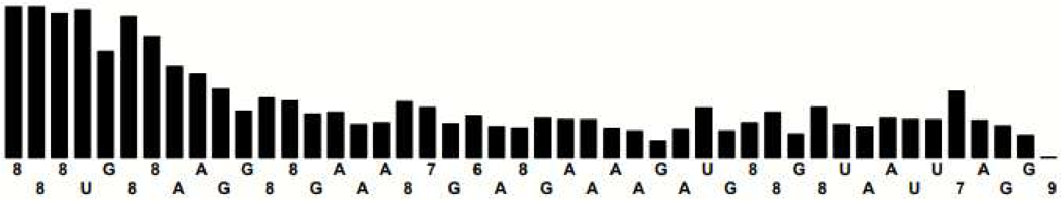

Figure 137. Trityl Monitor of the Synthesis of MinE07UC-Biotin on Universal UnyLinker Solid Support Cytosine is labelled as 8, fU-Ph is labelled at 6, fU-Vi is labelled as 7 and the biotin is labelled as 9. The trityl monitor gave a coupling efficiently of 1.47e[6].

Sequence {5'-3'}

9GG A7U UAA U8G 88G UAG AAA AG8 A6G 78A AAG 88G GAA 88G U88
8

Trityl Histogram for synthesis: ARP-BIOMINE07-139C8 46 mer.
Efficiency(Stepwise Yield by base leveling): 96.6%
Final Yield (by rolling average): 100.0%
Stepwise Yield for Monomer: 8: 91.3 %
Stepwise Yield for Monomer: U: 96.9 %
Stepwise Yield for Monomer: G: 93.9 %
Stepwise Yield for Monomer: A: 94.1 %
Stepwise Yield for Monomer: 7: 100.0 %
Stepwise Yield for Monomer: 6: 100.0 %
Stepwise Yield for Monomer: 9: 100.0 %

Figure 138. Aptamer Synthesis Report of the Synthesis of MinE07UC-Biotin on Universal UnyLinker Solid Support. The step wise coupling efficiency is 96.3%. The overall average yield: $\boldsymbol{0.966^{49} = 0.184}$. This gives an 18.4% overall synthetic yield.

MinE07UC'-Biotin: 5'-Biotin-rGrGrAfCrGrGrAfU-VifUfUrArAfUfCrGfCfCrGfUrArGrArArArArGfCrAfU-PhrGfU-VifCrArArArGfCfCrGrGrArAfCfCrGfUfCfC-3'

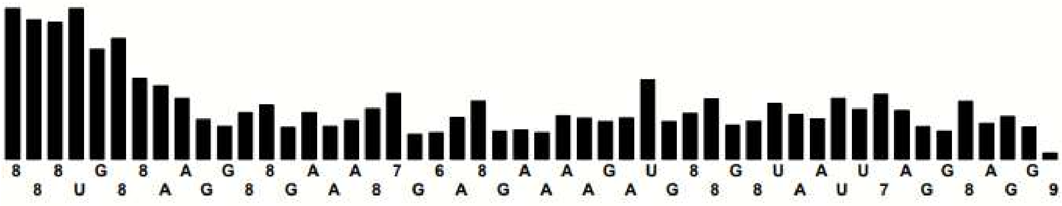

Figure 139. Trityl Monitor of the Synthesis of **MinE07UC'-Biotin** on Universal UnyLinker Solid Support Cytosine is labelled as 8, fU-Ph is labelled at 6, fU-Vi is labelled as 7 and the biotin is labelled as 9. The trityl monitor gave a coupling efficiently of $1.38e^{6}$.

Sequence {5'-3'}

9GG A8G GA7 UUA AU8 G88 GUA GAA AAG 8A6 G78 AAA G88 GGA A88
GU8 88

Trityl Histogram for synthesis: ARP-BIOMINE07-1392C8 50 mer.
Efficiency(Stepwise Yield by base leveling): 96.9%
Final Yield (by rolling average): 100.0%
Stepwise Yield for Monomer: 8: 91.9 %
Stepwise Yield for Monomer: U: 97.8 %
Stepwise Yield for Monomer: G: 93.1 %
Stepwise Yield for Monomer: A: 95.4 %
Stepwise Yield for Monomer: 7: 100.0 %
Stepwise Yield for Monomer: 6: 100.0 %
Stepwise Yield for Monomer: 9: 100.0 %

Figure 140. Aptamer Synthesis Report of the Synthesis of **MinE07UC'-Biotin** on Universal UnyLinker Solid Support. The step wise coupling efficiency is 96.3%. The overall average yield: $\boldsymbol{0.969^{49} = 0.214}$. This gives a 21.4% overall synthetic yield.

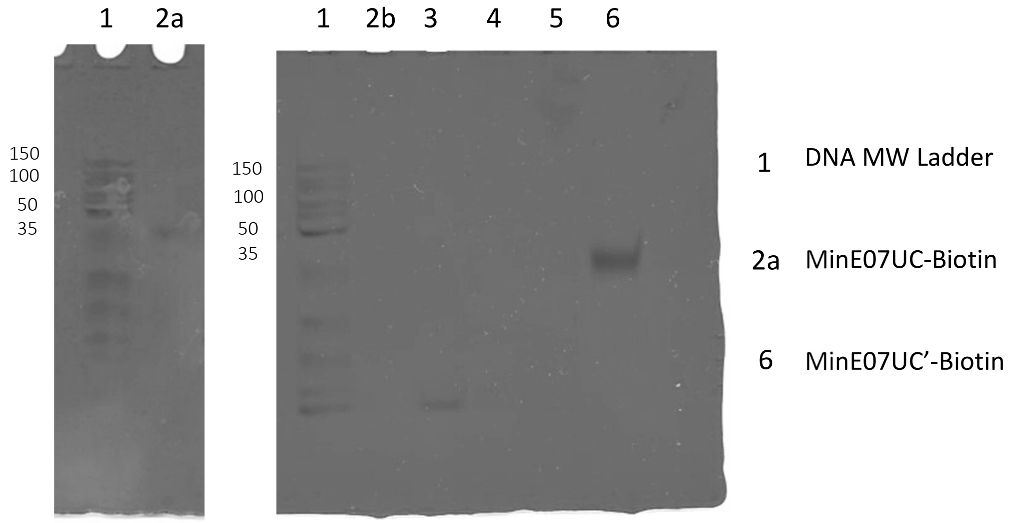

Figure 141. 15% Polyacrylamide Gel of **MinE07UC'-Biotin** and **MinE07UC'-Biotin** after PAGE purification.

MinE07-U-Ph-Biotin:5'-Biotin-rGrGrAfCrGrGrAfU-PhfU-PhfU-PhrArAfU-PhfCrGfCfCrGfU-PhrArGrArArArArGfCrAfU-PhrGfU-PhfCrArArArGfCfCrGrGrArAfCfCrGfU-PhfCfC-3'.

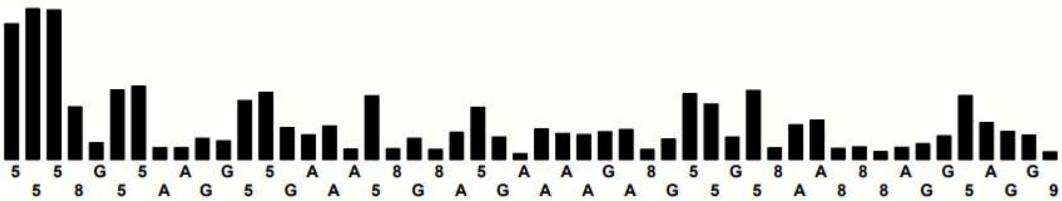

Figure 142. Trityl Monitor of the Synthesis of **MinE07U-Ph-Biotin** on Universal UnyLinker Solid Support. Cytosine is labelled as 5, fU-Ph is 8 and the biotin is 9.

Sequence {5'-3'}

9GG A5G GA8 88A A85 G55 G8A GAA AAG 5A8 G85 AAA G55 GGA A55
G85 55

Trityl Histogram for synthesis: ARPBIOMINE07A 50 mer.
Efficiency(Stepwise Yield by base leveling): 98.4%
Final Yield (by rolling average): 100.0%
Stepwise Yield for Monomer: 5: 97.2 %
Stepwise Yield for Monomer: 8: 94.7 %
Stepwise Yield for Monomer: G: 100.0 %
Stepwise Yield for Monomer: A: 100.0 %
Stepwise Yield for Monomer: 9: 100.0 %

Figure 143. Aptamer Synthesis Report of the Synthesis of **MinE07-U-Ph-Biotin** on Universal UnyLinker Solid Support. The step wise coupling efficiency is 98.4%. The overall average yield: $\mathbf{0.984^{49} = 0.454}$. This gives a 45.4% overall synthetic yield.

MinE07-U-Vi-Biotin:5'-Biotin-rGrGrAfCrGrGrAfU-VifU-VifU-VirArAfU-VifCrGfCfCrGfU-VirArGrArArArArGfCrAfU-VirGfU-VifCrArArArGfCfCrGrGrArAfCfCrGfU-VifCfC-3'

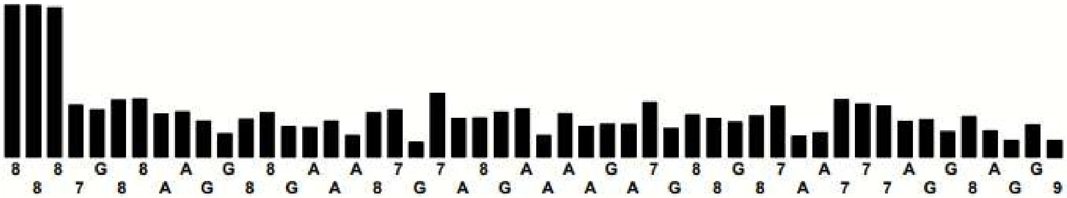

Figure 144. Trityl Monitor of the Synthesis of **MinE07U-Vi-Biotin** on Universal UnyLinker Solid Support Cytosine is labelled as 8, fU-Vi is 7 and biotin is 9.

Sequence {5'-3'}

9GG A8G GA7 77A A78 G88 G7A GAA AAG 8A7 G78 AAA G88 GGA A88
G78 88

Trityl Histogram for synthesis: ARP-BIOMINE07-U-VI 50 mer.
Efficiency(Stepwise Yield by base leveling): 97.8%
Final Yield (by rolling average): 100.0%
Stepwise Yield for Monomer: 8: 93.6 %
Stepwise Yield for Monomer: 7: 100.0 %
Stepwise Yield for Monomer: G: 97.1 %
Stepwise Yield for Monomer: A: 98.5 %
Stepwise Yield for Monomer: 9: 100.0 %

Figure 145. Aptamer Synthesis Report of the Synthesis of **MinE07U-Vi-Biotin** on Universal UnyLinker Solid Support. The step wise coupling efficiency is 97.8%. The overall average yield: $\mathbf{0.978^{49} = 0.3362}$. This gives a 33.62% overall synthetic yield.

MinE07-U-I-Biotin:5'-Biotin-rGrGrAfCrGrGrAfU-IfU-IfU-IrArAfU-IfCrGfCfCrGfU-IrArGrArArArArGfCrAfU-IrGfU-IfCrArArArGfCfCrGrGrArAfCfCrGfU-IfCfC-3'.

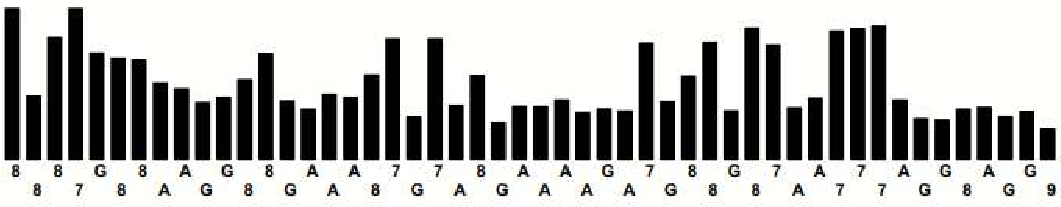

Figure 146. Trityl Monitor of the Synthesis of **MinE07U-I-Biotin** on Universal UnyLinker Solid Support Cytosine is labelled as 8, fU-I is 7 and biotin is 9.

Sequence {5'-3'}

9GG A8G GA7 77A A78 G88 G7A GAA AAG 8A7 G78 AAA G88 GGA A88
G78 88

Trityl Histogram for synthesis: ARP-BIOMINE07-U-I 50 mer.
Efficiency(Stepwise Yield by base leveling): 96.1%
Final Yield (by rolling average): 100.0%
Stepwise Yield for Monomer: 8: 87.7 %
Stepwise Yield for Monomer: 7: 99.3 %
Stepwise Yield for Monomer: G: 95.8 %
Stepwise Yield for Monomer: A: 97.7 %
Stepwise Yield for Monomer: 9: 100.0 %

Figure 147. Aptamer Synthesis Report of the Synthesis of **MinE07-U-I-Biotin** on Universal UnyLinker Solid Support. The step wise coupling efficiency is 96.1%. The overall average yield: $\boldsymbol{0.961^{49} = 0.1424}$. This gives a 14.24% overall synthetic yield.

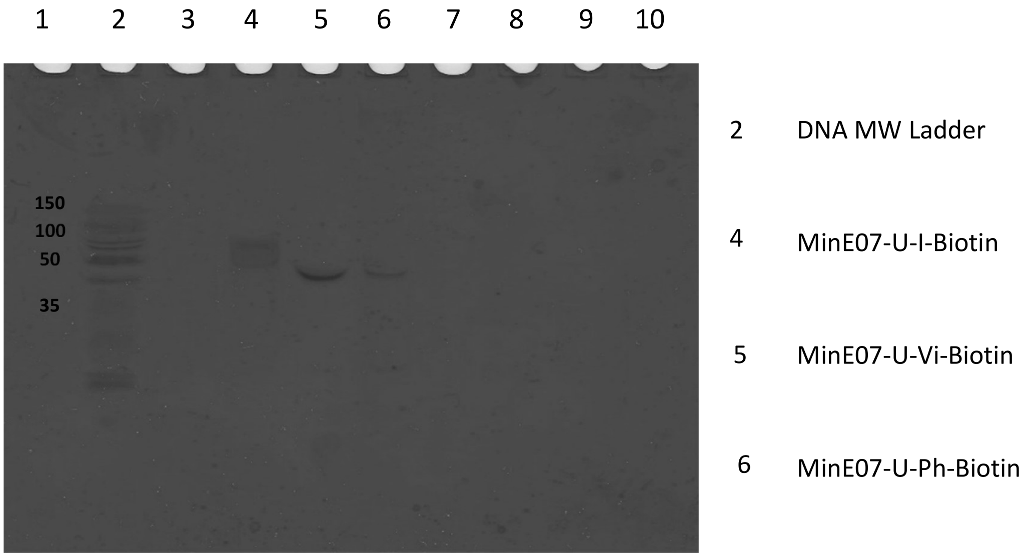

Figure 148. 15% Polyacrylamide Gel of **MinE07-U-Ph-Biotin**, **MinE07-U-Vi-Biotin** and **MinE07-U-I-Biotin** after PAGE purification.

HABA Biotin Binding Assay with MinE07-Biotin, MinE07UA-Biotin, MinE07UA'-Biotin, MinE07UB-Biotin, MinE07UC-Biotin and MinE07UC'-Biotin Aptamers. Streptavidin-AP at a 1:1000 dilution in binding buffer was prepared, 20 µL was added to each well and it was incubated for 30 minutes at room temperature shaking at 450 rpm with 20 µl of Tropix CDP-Star ready to use substrate. The plate was then read using the chemiluminescence protocol on the Victor X4 plate reader to check it was giving a signal. 20 µL of HABA (5 µM) was added to the wells and it was incubated for 30 minutes at room temperature shaking at 450rpm. MinE07UA-Biotin, MinE07UA'-Biotin, MinE07UB-Biotin, MinE07UC-Biotin and MinE07UC'-Biotin were added to relevant wells and incubated for 30 minutes at room temperature shaking at 450rpm. The plate was then read using the chemiluminescence protocol on the Victor X4 plate reader.

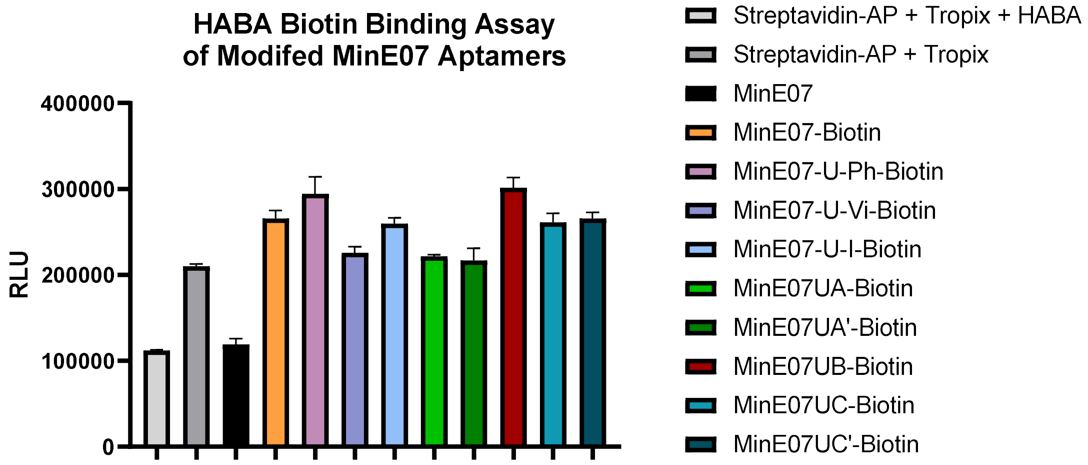

Figure 149. HABA Biotin Binding Assay Results for the modified MinE07 Aptamers (n=3). Negative control = Streptavidin-AP + Tropix + HABA and MinE07. Positive control = Streptavidin-AP + Tropix and MinE07-Biotin.

S8. Aptamer Validation

EGFR Binding Assay with MinE07UA-Biotin, MinE07UA'-Biotin, MinE07UB-Biotin, MinE07UC-Biotin and MinE07UC'-Biotin. The protein A plates were washed 3 times with 150 µL of wash buffer per well. EGFR-Fc protein was prepared in wash buffer at 1 µg/mL. 100 µL EGFR-Fc coating buffer per well was added and incubated for 30 minutes at 450 rpm at room temperature. The liquid was flicked out of the well plate. The plate was washed 3 times with wash buffer and then 50 µL 1x binding buffer to each well was added, and then incubate at 450 rpm for 10 minutes. MinE07UA-Biotin, MinE07UA'-Biotin, MinE07UB-Biotin, MinE07UC-Biotin and MinE07UC'-Biotin aptamers were prepared at concentrations 500 nM, 158 nM, 50 nM, 15.8 nM, 5 nM, 1.58 nM, 1 nM and 0.5 nM in binding buffer. The diluted aptamer was then denatured in the Eppendorf master cycler PCR machine (85°C for 5 minutes, then cooled to 25°C at 0.1°C per second and held at 25°C). The binding buffer was removed from the plate and washed 3 times with wash buffer. 100 µL of aptamer was added to the relevant wells. This was then incubated for 60 minutes at room temperature at 450 rpm. The liquid was flicked out of the well plate, and it was washed 3 times with wash buffer. Streptavidin-AP at a 1:1000 dilution in binding buffer was prepared, 100 µL was added to each well and it was incubated for 30 minutes at room temperature shaking at 450 rpm. The plate was then washed with wash buffer and then 95 µL of Tropix CDP-Star ready to use substrate was added and incubated for 10 minutes with shaking at 450rpm. The plate was then read again using the chemiluminescence protocol was then read on the Victor X4 plate reader. This was repeated until 3 biological repeats were achieved. Data analysis was done on Prism GraphPad Version 9.0.0 using the one site - specific binding model to calculated Bmax and K_d.

MinE07-Biotin

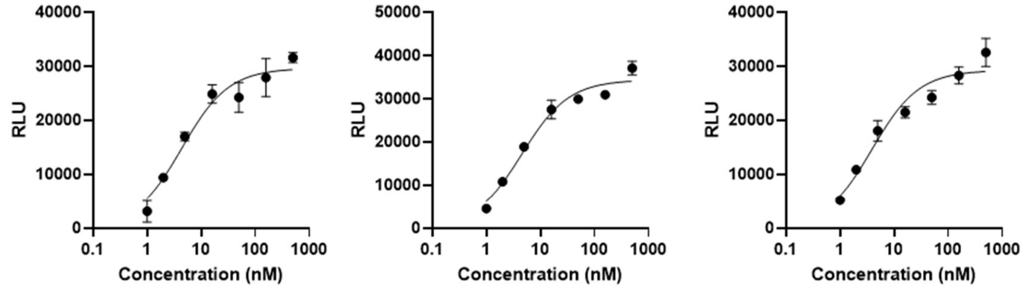

Bmax	28385
Kd	3.923

Bmax	32588
Kd	3.958

Bmax	27263
Kd	3.208

Figure 150. Protein Binding Affinity Assay Graphs of **MinE07-Biotin**

MinE07-U-Ph-Biotin

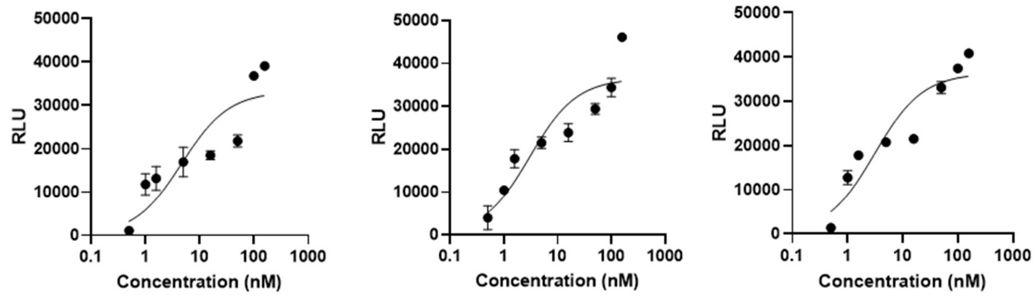

Bmax	33228
Kd	4.654

Bmax	36471
Kd	3.018

Bmax	36354
Kd	3.097

Figure 151. Protein Binding Affinity Assay Graphs of **MinE07-U-Ph**

MinE07-U-Vi-Biotin

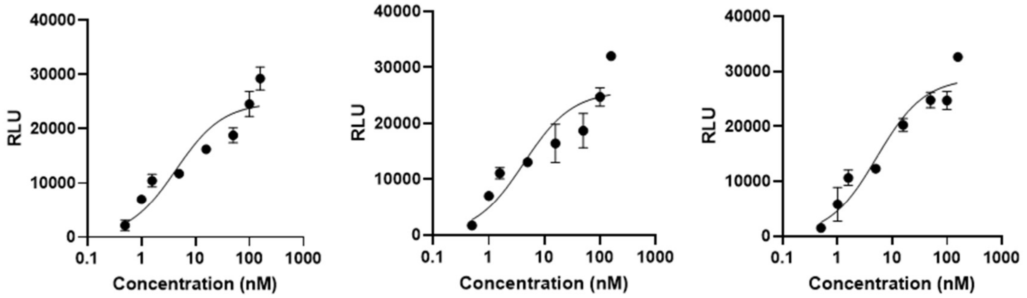

Bmax	24863
Kd	4.355

Bmax	25777
Kd	4.273

Bmax	28817
Kd	5.236

Figure 152. Protein Binding Affinity Assay Graphs of **MinE07-U-Vi**

MinE07-U-I-Biotin

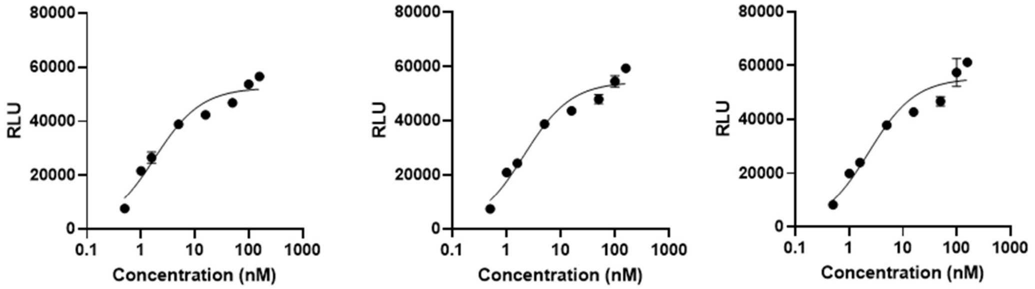

Bmax	52450
Kd	1.778

Bmax	54337
Kd	2.087

Bmax	55389
Kd	2.289

Figure 153. Protein Binding Affinity Assay Graphs of **MinE07-U-I**

MinE07UA-Biotin

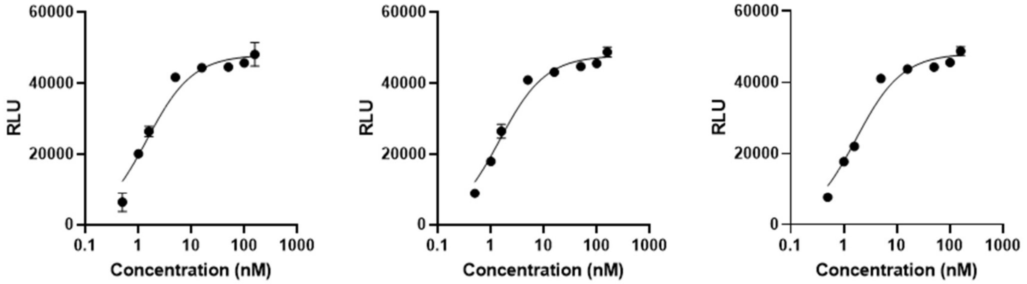

Bmax	48008
Kd	1.439

Bmax	47814
Kd	1.458

Bmax	48165
Kd	1.693

Figure 154. Protein Binding Affinity Assay Graphs of **MinE07A**

MinE07UA'-Biotin

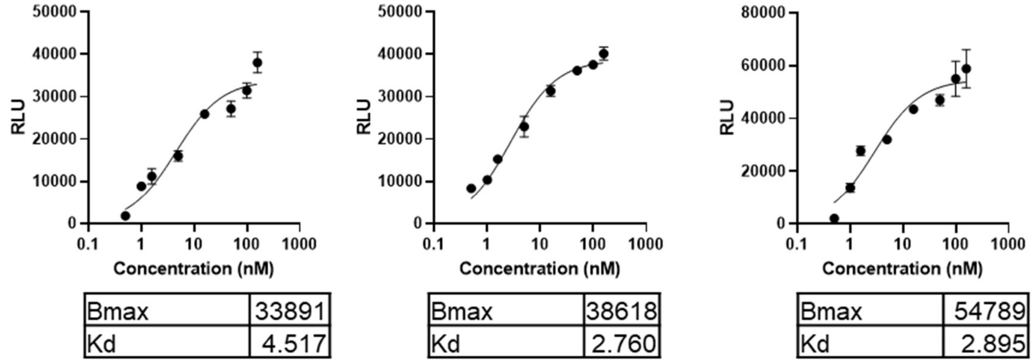

Figure 155. Protein Binding Affinity Assay Graphs of **MinE07A'**

MinE07UB-Biotin

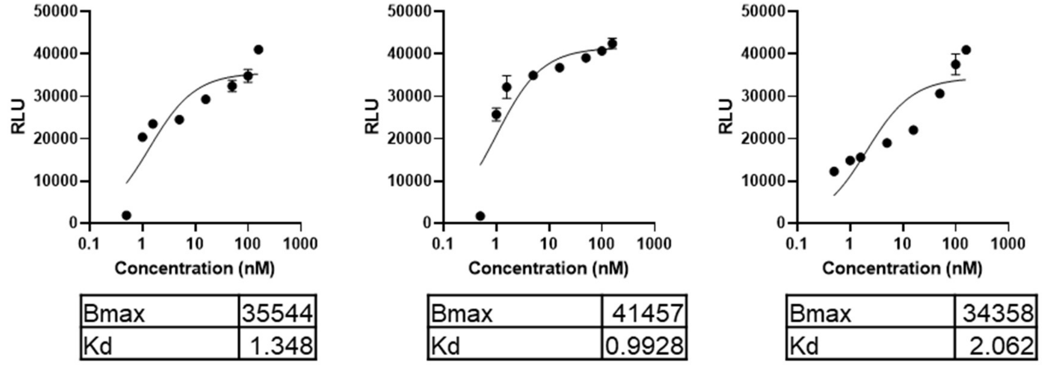

Figure 156. Protein Binding Affinity Assay Graphs of **MinE07B**

MinE07UC-Biotin

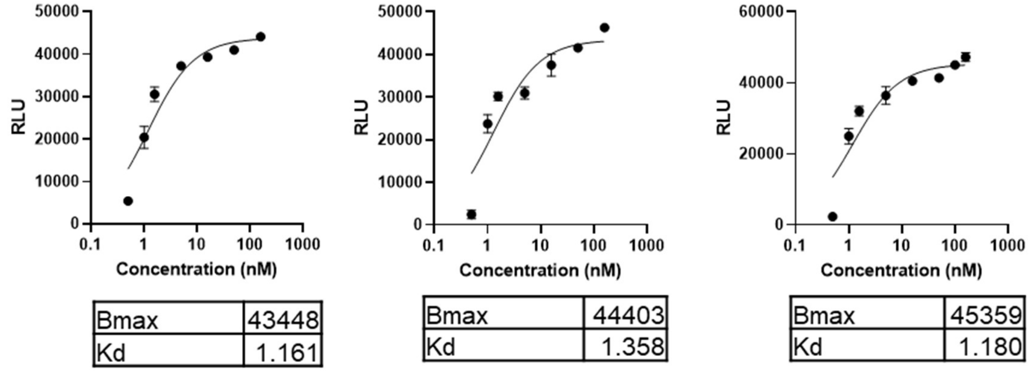

Figure 157. Protein Binding Affinity Assay Graphs of **MinE07C**

MinE07UC’-Biotin

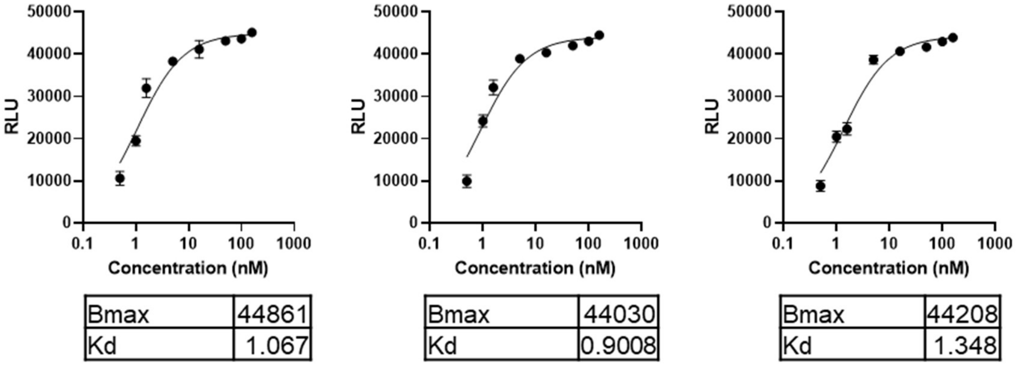

Figure 158. Protein Binding Affinity Assay Graphs of **MinE07C’**

Table 2. Aptamer binding affinity t-test analysis 95% and 99% confidence intervals generated from Prism GraphPad Version 9.0.0 (significance P< 0.01). The t-test was parent MinE07-Biotin vs all other novel aptamers.

**Aptamer**	**95% Confidence intervals**	**99% Confidence intervals**
**MinE07-Biotin**	-	-
**MinE07-U-Ph-Biotin**	-1.734 to 1.520	-2.805 to 2.592
**MinE07-U-Vi-Biotin**	-0.167 to 2.017	-0.886 to 2.736
**MinE07-U-I-Biotin**	-2.439 to -0.851	-2.962 to -0.3282
**MinE07UA-Biotin**	-2.882 to -1.451	-3.353 to -0.980
**MinE07UA'-Biotin**	-2.014 to 1.402	-3.138 to 2.526
**MinE07UB-Biotin**	-3.334 to -1.123	-4.062 to -0.395
**MinE07UC-Biotin**	-3.164 to -1.763	-3.625 to -1.302
**MinE07UC'-Biotin**	-3.360 to -1.822	-3.867 to -1.316

S9. Computational Docking Studies

ViennaRNA 2.0 was used to model a 2D predicted secondary structure of the MinE07 aptamers, using the thermodynamic structure prediction and the RNAfold web server.[9] The dot and bracket sequence was used to generate a PDB file to create 3D structure using RNA composer.[10,11] The PDB file was opened in text editor and all the 2'OH atoms are deleted from uridine and cystine. This PDB can be visualised using the cross platform molecular builder and editor program, Avogadro (avogadro.cc).[12] The geometry was then optimised using the optimise geometry option in Avogadro with force field MMFF94, which is in a vacuum. EGFR PBD file (1IVO) was taken from the Protein Data Bank.[13] For docking studies the following programs were used Autodock Tools (MGLTools 1.5.7) available from: mgltools.scripps.edu/downloads, Autodock Vina 1.1.2[14] run via MPI Vina on a multi-node cluster and PyMOL 2.5.3 Schrödinger L, DeLano W. PyMOL 2020, available from: pymol.org/pymol. Blind docking was first undertaken, parameters: box position (X Y Z, Å) 83.72, 56.20, 50.18, size (Å) 126x126x126. Docking on binding site were then undertaken with the parameters: box position (X Y Z, Å) 93.00, 82.00, 72.00, size (Å) 61.24x45.68x44.70.[15]

```
ATOM    285  C2'   U A   9      -2.379 -17.851 -16.606  1.00  0.00           C
ATOM    286  H2'   U A   9      -2.042 -16.833 -16.402  1.00  0.00           H
ATOM    287  O2'   U A   9      -1.492 -18.832 -16.101  1.00  0.00           O
ATOM    288 HO2'   U A   9      -1.937 -19.252 -15.364  1.00  0.00           H
```

Figure 159. As all control MinE07 aptamers have fluorine at the 2' position of all the uridines and cytidines residues, the PDB file was edited using notepad. All the 2'OH atoms are deleted from uridine and cytidines.PBD File of MinE07 aptamer before editing.

```
ATOM    283  C2' U   A   9      -2.332 -17.991 -16.814  1.00  0.00           C
ATOM    284  H2' U   A   9      -1.836 -17.068 -16.520  1.00  0.00           H
ATOM    285  F2' U   A   9      -1.513 -19.038 -16.468  1.00  0.00           F
```

Figure 160. PBD File of MinE07 aptamer after editing. The O2' lines were then replaced with F2'.

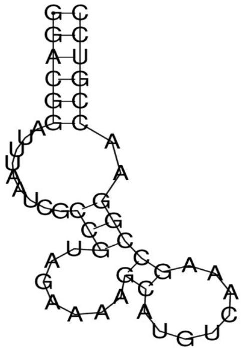

Figure 161. 2D Predicted secondary structure of MinE07 aptamer. The results for the optimal secondary structure gave a minimum free energy of -9.30kcal/mol. ViennaRNA was used to model a 2D predicted secondary structure of the **control MinE07** aptamers (rna.tbi.univie.ac.at/). This was done by using the thermodynamic structure prediction and the RNAfold web server.

((((((.........(((.......((........)))))..))))))

Figure 162. The MinE07 dot and bracket sequence used to generate a PDB file. The dot and bracket sequence was used to generate a PDB file to create 3D structure using RNA composer (rnacomposer.cs.put.poznan.pl/).

S10 References

1 TentaGel® M NH_2 Monosized Amino TentaGel Microspheres, http://www.rapp-polymere.com/index.php?id=891.

2 M. Jora, P. A. Lobue, R. L. Ross, B. Williams and B. Addepalli, *Biochimica et Biophysica Acta (BBA) - Gene Regulatory Mechanisms*, 2019, **1862**, 280–290.

3 P. Thakur, S. Abernathy, P. A. Limbach and B. Addepalli, in *Methods in Enzymology*, 2021, **658**, 1–24.

4 K. Brunner, J. Harder, T. Halbach, J. Willibald, F. Spada, F. Gnerlich, K. Sparrer, A. Beil, L. Möckl, C. Bräuchle, K.-K. Conzelmann and T. Carell, *Angewandte Chemie International Edition*, 2015, **54**, 1946–1949.

5 P. A. Lichtor, Z. Chen, N. H. Elowe, J. C. Chen and D. R. Liu, *Nat Chem Biol*, 2019, **15**, 419–426.

6 G. Vera, B. Diethelm, C. A. Terraza and G. Recabarren-Gajardo, *Molecules*, 2018, **23**, 2051.

7 Z. Chen, P. A. Lichtor, A. P. Berliner, J. C. Chen and D. R. Liu, *Nat Chem*, 2018, **10**, 420–427.

8 P. J. Sample, K. W. Gaston, J. D. Alfonzo and P. A. Limbach, *Nucleic Acids Res*, 2015, **43**, 1–13.

9 R. Lorenz, S. H. Bernhart, C. Höner zu Siederdissen, H. Tafer, C. Flamm, P. F. Stadler and I. L. Hofacker, *Algorithms for Molecular Biology*, 2011, **6**, 26.

10 M. Antczak, M. Popenda, T. Zok, J. Sarzynska, T. Ratajczak, K. Tomczyk, R. W. Adamiak and M. Szachniuk, *Acta Biochim Pol*, 2016, **63**, 737–744.

11 M. Popenda, M. Szachniuk, M. Antczak, K. J. Purzycka, P. Lukasiak, N. Bartol, J. Blazewicz and R. W. Adamiak, *Nucleic Acids Res*, 2012, **40**, 112.

12 M. D. Hanwell, D. E. Curtis, D. C. Lonie, T. Vandermeersch, E. Zurek and G. R. Hutchison, *J Cheminform*, 2012, **4**, 17.

13 H. Ogiso, R. Ishitani, O. Nureki, S. Fukai, M. Yamanaka, J. H. Kim, K. Saito, A. Sakamoto, M. Inoue, M. Shirouzu and S. Yokoyama, *Cell*, 2002, **110**, 775–787.

14 O. Trott and A. J. Olson, *J Comput Chem*, 2009, **31**, 455–461.

15 D. Ghersi and R. Sanchez, *Proteins*, 2009, **74**, 417–24.
